# A catalogue of 1,167 genomes from the human gut archaeome

**DOI:** 10.1038/s41564-021-01020-9

**Published:** 2021-12-30

**Authors:** Cynthia Maria Chibani, Alexander Mahnert, Guillaume Borrel, Alexandre Almeida, Almut Werner, Jean-François Brugère, Simonetta Gribaldo, Robert D. Finn, Ruth A. Schmitz, Christine Moissl-Eichinger

**Affiliations:** 1 Institute for Microbiology, Christian-Albrechts-University Kiel, Kiel, Germany; 2 Diagnostic & Research Institute of Hygiene, Microbiology and Environmental Medicine, Medical University Graz, Graz, Austria; 3 Department of Microbiology, Unit of Evolutionary Biology of the Microbial Cell, Institut Pasteur, Paris, France; 4 European Molecular Biology Laboratory, European Bioinformatics Institute, Cambridge, UK; 5 Wellcome Sanger Institute, Cambridge, UK; 6 Institut Universitaire de Technologie Clermont Auvergne, Université Clermont Auvergne, CNRS, UMR 6023 Laboratoire Microorganismes: Genome et Environnement, Clermont-Ferrand, France; 7 BioTechMed, Graz, Austria

**Keywords:** Archaea, Microbial genetics

## Abstract

The human gut microbiome plays an important role in health, but its archaeal diversity remains largely unexplored. In the present study, we report the analysis of 1,167 nonredundant archaeal genomes (608 high-quality genomes) recovered from human gastrointestinal tract, sampled across 24 countries and rural and urban populations. We identified previously undescribed taxa including 3 genera, 15 species and 52 strains. Based on distinct genomic features, we justify the split of the *Methanobrevibacter smithii* clade into two separate species, with one represented by the previously undescribed ‘*Candidatus* *Methanobrevibacter intestini*’. Patterns derived from 28,581 protein clusters showed significant associations with sociodemographic characteristics such as age groups and lifestyle. We additionally show that archaea are characterized by specific genomic and functional adaptations to the host and carry a complex virome. Our work expands our current understanding of the human archaeome and provides a large genome catalogue for future analyses to decipher its impact on human physiology.

## Abstract (web summary)

Recovery of 1,167 nonredundant archaeal genomes from the human gut microbiomes reveals previously undescribed genera, associations with sociodemographic factors and the presence of an archaeal virome.

## Main

The human microbiome is increasingly recognized as a key player in human health^1^. Although most research has focused on the bacterial component^2^ and its bacteriophages^3,4^, and to some extent unicellular eukaryotes (including fungi) and their viruses, the archaea have been largely overlooked, mainly due to methodological reasons^5–9^.

Archaea are prokaryotes, like bacteria, but are different in cell structure, metabolism and molecular machinery (summarized in ref. ^9^). Archaea linked with the human gut microbiome are mainly methanogenic archaea, of which only a few have been isolated. Methanogenesis is a unique metabolic process, during which C_1_ or C_2_ carbon compounds, such as CO_2_, CO, formate, acetate or methyl compounds serve as substrates for the formation of methane. It is a highly syntrophic metabolism, as end-products of bacterial fermentation are consumed.

The most prevalent archaea in the human gut are Methanobacteriales and Methanomassiliicoccales. Methanobacteriales are mainly represented by *Methanobrevibacter smithii* (prevalence of up to 97.5%) and *Methanosphaera stadtmanae* (prevalence of up to 23%^10–12^). Methanomassiliicoccales have only recently been discovered and identified in the human gut, with *Methanomassiliicoccus luminyensis*^13^, *Candidatus* *Methanomassiliicoccus intestinalis*^14^, *Ca*. *Methanomethylophilus alvus*^15^, and the strains Mx02, Mx03 and Mx06, being most prevalent (up to 80%^16^). Numerous additional archaeal signatures have been retrieved by amplicon- and metagenome-based microbiome analyses, indicating the presence of a complex archaeome in the human gastrointestinal tract (GIT)^8,17,18^.

Some archaea carry adaptive traits for colonization of the human gut environment, such as bile salt hydrolases^19^ and adhesin-like proteins^16,20^. Besides, archaea can degrade deleterious bacterial metabolites such as trimethylamine (TMA)^16,21,22^ and can induce specific host immune responses^7,23,24^. Overall, the role of the human archaeome, particularly in health and disease^6,9^, still needs to be explored, with the most puzzling question, whether archaeal pathogens do exist, as an intrinsically pathogenic capacity of archaea has never been identified.

Based on the recent activities to generate and collect thousands of metagenome-assembled genomes (MAGs) from metagenomic datasets of human GIT^2,25–27^, a treasure of information was produced. In the present study, we present a public catalogue composed of 1,167 archaeal genomes and 28,581 protein clusters derived from the human gastrointestinal archaeal community. Leveraging this comprehensive sequence collection, we gain previously undescribed insights into the abundance, distribution, composition and function of the human archaeome.

## Results

### Over 1,000 unique archaeal genomes recovered from human gastrointestinal samples

**Fig. 1:**
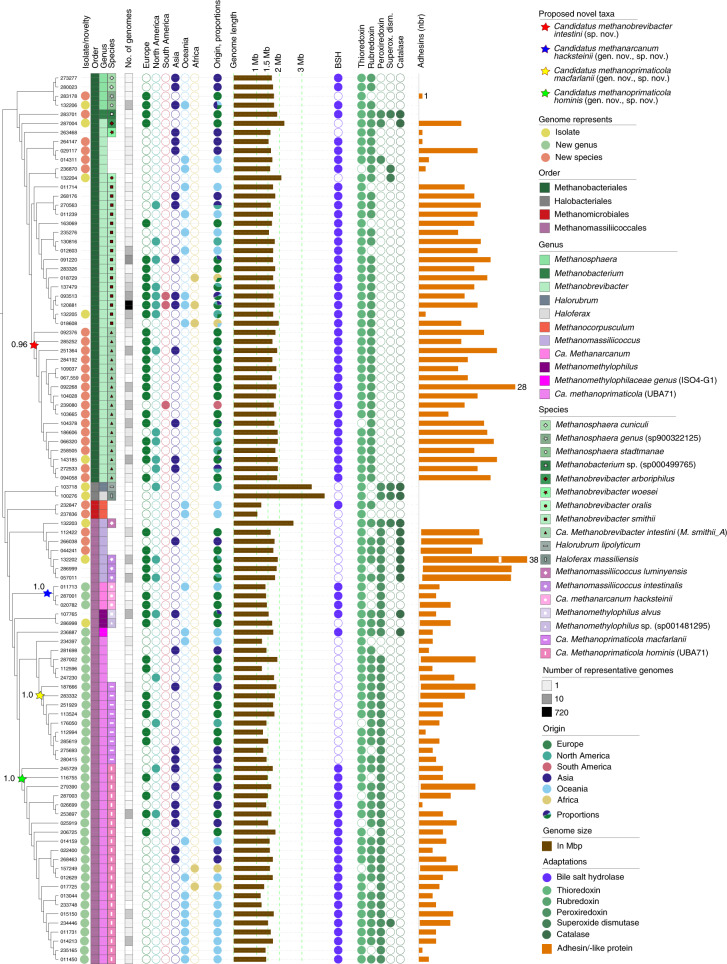
Archaeal genomes (1,167) from the human GIT reveal taxonomic expansion of the archaeome. Phylogenetic tree of genomes clustered at 99% similarity (‘strains’), shown with the following characteristics (from left to right): proposed original taxa (indicated by stars on the branch of the phylogenetic tree), including ultrafast bootstrap values. Species representatives are highlighted by bold genome numbers. Isolates, representatives of unknown genera and species are indicated by a coloured dot next to the genome number. Taxonomic affiliation of representative genomes is shown at order, genus and species level. The number of genomes assigned to the strain-level taxon is shown in the grey histogram. The origin displays the origin of the samples from which this genome and its representatives could be assembled. The pie chart displays the proportion of the origins. The respective genome size of the representative genome is displayed in megabases (Mb; brown bars). There is an overview of the absence and presence of genes involved in host interactions: with bile salt hydrolases (blue; BSH) and oxygen resistance genes (green), and the presence of genomes potentially coding for adhesins/adhesin-like/‘Flg_new’ domain^16^ proteins (orange). Genomes (strain list) were analysed using MaGe Microscope and genes were counted as present when automatic annotation was positive (‘putative’ annotation was counted as positive).

**Extended Data Fig. 1:**
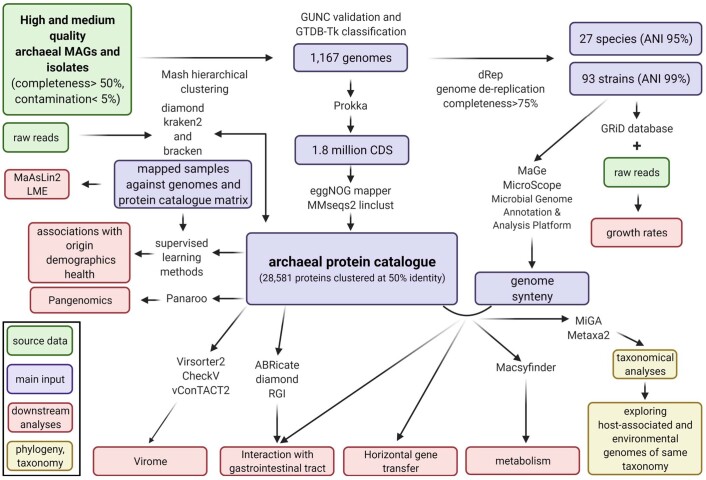
Methodology. Flow chart covering the major analysis steps of the study. Colored boxes show the source data (green), main input for the analysis (magenta), downstream analysis (red) and the taxonomic analysis of the presented data set (yellow). Different steps are connected by arrows highlighting a selection of used bioinformatic tools for each step. For details on the genomes, software and databases used, please refer to Supplementary Table 1 and Supplementary Table 14. Figure created with biorender.com.

**Extended Data Fig. 2:**
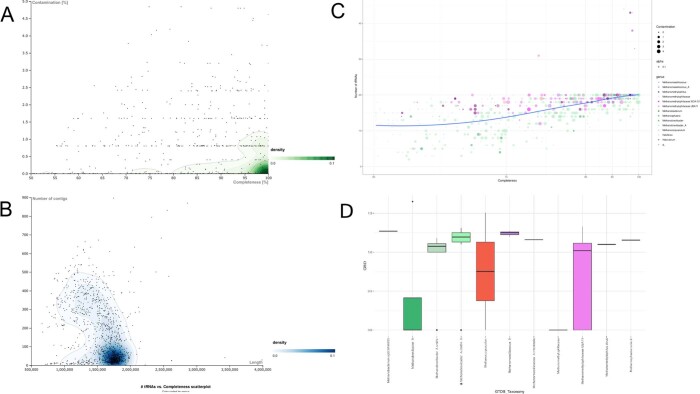
Overview on the quality of 1,167 genomes summarized in Supplementary Table 1a. **a**) Contour plot of genome completeness vs. genome contamination based on CheckM MAGs quality estimates. **b**) Contour plot of genome length vs. number of contigs. **c**) Number of predicted tRNAs vs. completeness for each of the 1,167 genomes represented in a scatter plot color coded by taxonomic assignment at the genus level. The size of each data point is relative to genome contamination estimation. Locally Weighted Least Squares Regression (LOESS) method used for smoothing (blue line). **d**) Growth rate indices (GRiD) of archaeal genomes from the human gut based on 58 GTDB classified genomes. *Candidatus *Methanobrevibacter* *intestini*. n = 58 independent genomes, boxplot specifications: colored box de- fines the interquartile range (lower boundry 25^th^ percentile, median 50^th^ percentile and upper boundry 75^th^ percentile), whiskers repre- sent smallest and largest values within 1.5 times of the interquartile range above the 75^th^ percentile and below the 25^th^ percentile re- spectively. Individual dots represent outside values between 1.5 and 3 times of the interquartile range.

To explore the diversity of archaea in human gastrointestinal samples, we compiled publicly available genomes from recent collections of MAGs and isolates. The retrieved 1,167 nonchimeric and nonredundant genomes (Extended Data Fig. 1) span a wide taxonomic diversity, and include members of the Methanobacteriales (87.15%), Methanomassiliicoccales (12.43%), Methanomicrobiales (0.26%) and Halobacteriales (0.17%; Supplementary Table 1a–f and Fig. 1). Most genomes were taxonomically affiliated with the known genus *Methanobrevibacter* (996 genomes; 85%), in agreement with earlier reports^9^. Other genomes were affiliated to the genera *Methanomethylophilus* (38; 3.3%), *Methanomassiliicoccus* (29; 2.5%), *Methanosphaera* (20; 1.7%) and *Methanocorpusculum* (3; 0.3%). *Methanobacterium, Haloferax* and *Halorubrum* spp. were represented by only one genome each. Of the 1,167 genomes, 10 (0.85%) could not be assigned to any previously described genus and 98 genomes (8.3%) did not match any known species. A large proportion of genomes not matching any known species (*n* = 83) and genera (*n* = 10) were affiliated with the order Methanomassiliicoccales. Read-based community profiling revealed a fraction of 1.22% archaeal reads in representative original datasets (Supplementary Table 2a–f). Based on growth rate index analyses, we received good evidence that the major archaeal species are indeed actively replicating within their habitat (see Supplementary Information and Extended Data Fig. 2). Pan-genome analyses (Supplementary Information) revealed that the gut archaeome still remains largely undersampled.

### Archaeal protein profile correlates with geographic and demographic parameters

**Extended Data Fig. 3:**
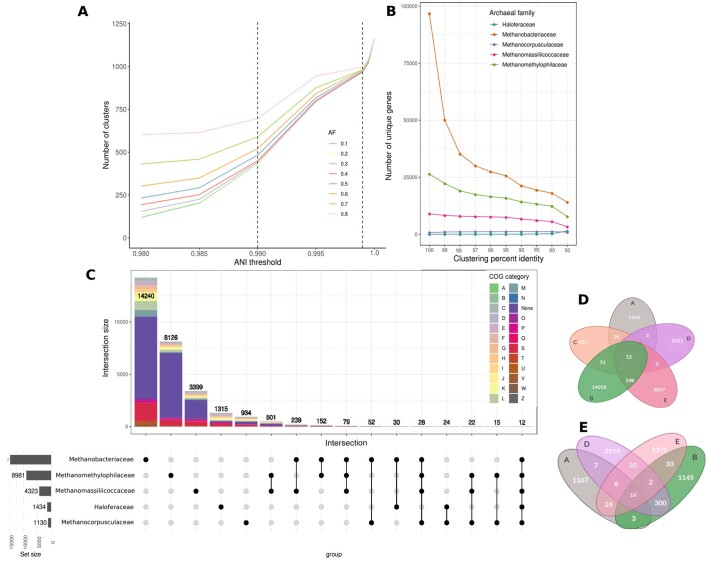
Genome dereplication, protein catalogue and protein functionality. **a**) Benchmarking different genome clustering thresholds. Number of clusters (that is, strains) identified according to the thresholds used by dRep for ANI and aligned fraction (AF). Vertical line indicates the chosen ANI threshold where the number of clusters begins to stabilize. The 99% ANI threshold was selected to sub-group genomes into a ‘strain’-list. **b**) Protein catalogue clustering at different percent identities. Line plots representing the number of unique proteins per archaeal family clustering at different percent identities. Drops are observed at 99-95% and 80-50% identity and 80% coverage. **c**) UpSet plot representing the frequency of COG categories based on the protein catalogue of the unique and shared proteins between the 5 archaeal MAGs taxonomic families (CELLULAR PROCESSES AND SIGNALING: [**d**] Cell cycle control, cell division, chromosome partitioning, [M] Cell wall/membrane/envelope biogenesis, [N] Cell motility, [O] Post-translational modification, protein turnover, and chaperones, [T] Signal transduction mechanisms, [U] Intracellular trafficking, secretion, and vesicular transport, [V] Defense mechanisms. INFORMATION STORAGE AND PROCESSING: [J] Translation, ribosomal structure and biogenesis, [K] Transcription, [L] Replication, recombination, and repair. METABOLISM: [C] Energy production and conversion, [E] Amino acid transport and metabolism, [F] Nucleotide transport and metabolism, [G] Carbohydrate transport and metabolism, [H] Coenzyme transport and metabolism, [I] Lipid transport and metabolism, [P] Inorganic ion transport and metabolism, [Q] Secondary metabolites biosynthesis, transport, and catabolism. POORLY CHARACTERIZED: [S] Function unknown) – Supplementary Material 1. The numbers in the vertical barplot represent the size of the unique (single dots) and shared proteins (connected dots) between the 5 archaeal taxonomic families while the numbers in the horizontal barplots represent the number of genomes per archaeal family. UpSet plot was done using the library UpSet in R. The 2 pairs of families that shared the higher numbers of protein clusters were Methanomethylophilaceae- Methanomassilliicoccaceae and Methanobacteriaceae- Methanomassilliicoccaceae. Shared protein clusters COG categories are Metabolism, Information, storage and processing, Cellular processes and signaling and have unknown functions. Shared proteins between the different archaeal families **d**) for all 1167 genomes **e**) for complete genomes only. Venn diagrams were done by creately (https://app.creately.com).

In total, 1.8 million proteins were identified from the 1,167 genomes, 54% of which were annotated as hypothetical proteins. A protein catalogue of all 1,167 archaeal genomes was generated by clustering the genes predicted across all genomes and excluding singleton clusters, resulting in 28,581 cluster representatives (>50% amino acid identity and >80% coverage) (Extended Data Fig. 3 and Supplementary Material 1). 2,050 proteins (thereof 58% hypothetical proteins) were found to be shared among >50 genomes in our dataset, mirroring the taxonomic distance of the two most abundant orders, Methanomassiliicoccales and Methanobacteriales (Fig. 3a).

**Fig. 2:**
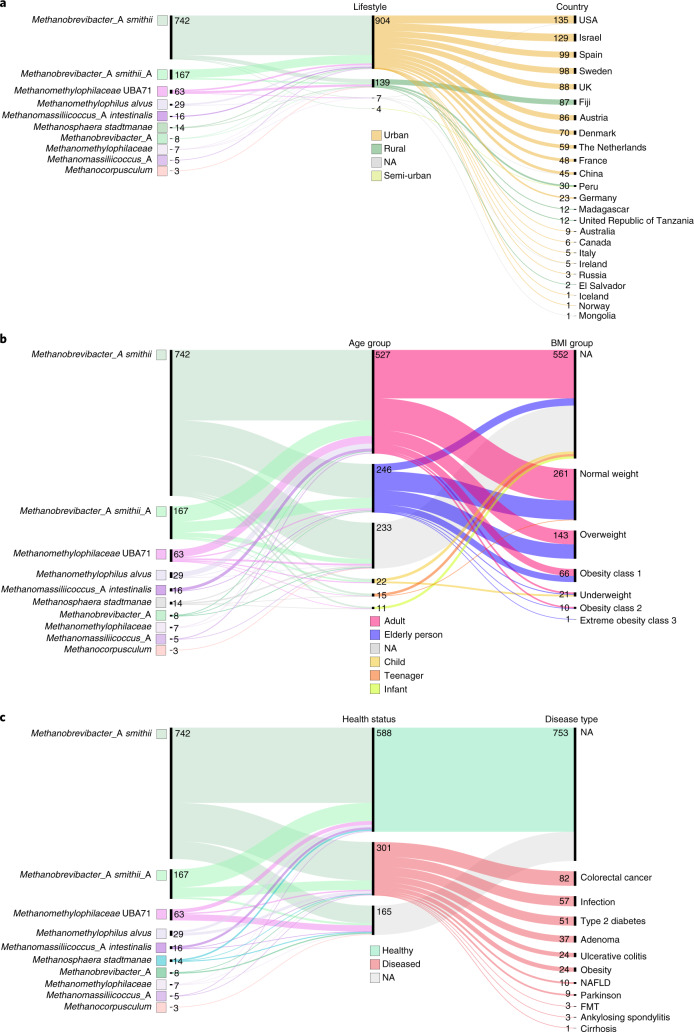
Genome distribution on different metadata categories covering geographic origin, demographics and health aspects. **a**,**b**, Categorical metadata were grouped in three alluvial diagrams referring to geographic origin (**a**, lifestyle and country) and demographics (**b**, age and BMI group). Obesity was defined as BMI > 30 kg m^−2^. Infant: 0–3 years; child: 4–12 years; teenager: 13–18 years; adult: 19–64 years; elderly person: >64 years. **c**, Health aspects (health status and disease type). NA, no data available. For improved visibility only genomes with a minimum of three representatives according to the GTDB classification are shown. Numbers indicate the amount of genomes in each group (1,054 archaeal genomes in total).

**Extended Data Fig. 4:**
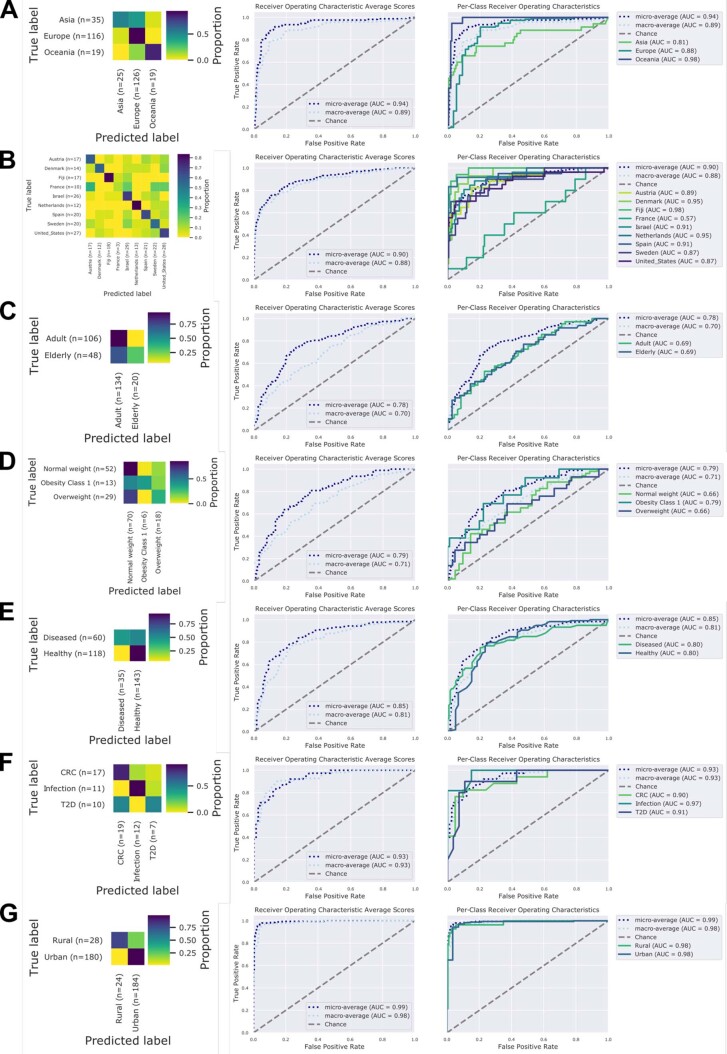
Predicting metadata values as a function of protein composition by supervised learning methods. Heatmaps and Receiver Operating Characteristic (ROC) curves of metadata predictions based on the unified archaeal MAG protein catalogue. AUC (area under the curve). Each tested metadata category was downsampled to a minimum of 50 genomes. Continent (**a**), country (**b**), age group (**c**), BMI group (**d**), health status (**e**), diseases (**f**), lifestyle (**g**).

**Extended Data Fig. 5:**
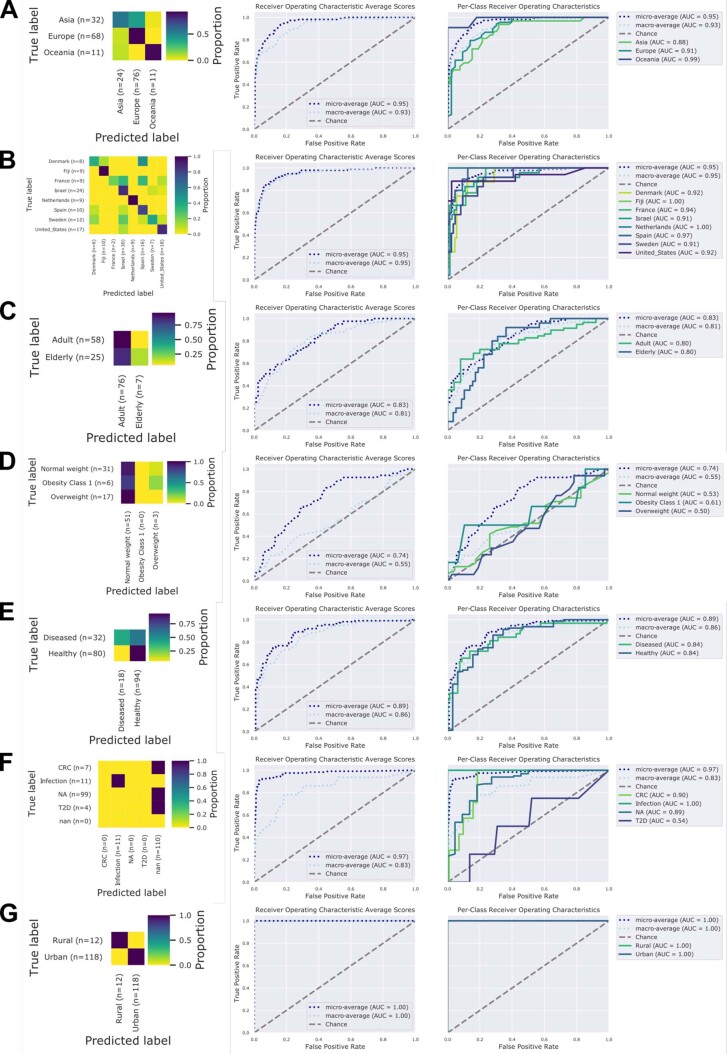
Predicting metadata values as a function of mapped sequences. Heatmaps and Receiver Operating Characteristic (ROC) curves of metadata predictions based on mapped reads against the unified archaeal MAG protein catalogue as a reference. AUC (area under the curve). Each tested metadata category was downsampled to a against the unified protein catalogue by supervised learning methods minimum of 50 genomes. Continent (**a**), country (**b**), age group (**c**), BMI group (**d**), health status (**e**), diseases (**f**), lifestyle (**g**).

The protein catalogue had predictive potential for some metadata categories (Fig. 2, Supplementary Tables 3 and 4, and Extended Data Figs. 4 and 5). Highest prediction accuracies were reached for the lifestyle (urban/rural) of an individual (overall accuracy = 100%). Prediction accuracies >70% were still reached for the continent, country, health status, age group or sex of an individual, whereas the body mass index (BMI) group was less suitable to build supervised learning models (prediction accuracies <70%) and achieved significance only when predictions were based on actual numerical BMI values rather than grouped BMI categories (*R* = 0.4, *P* = 2.9 × 10^−5^). For some metadata categories such as lifestyle, sex and origin per country of an individual, predictions improved if they were based on abundances (mapped protein matrix) rather than presence/absence (unified protein catalogue). Please refer to Supplementary Information for results on combinatory effects of multiple metadata categories, and on the association of hypothetical proteins with various metadata categories (Supplementary Tables 5 and 6).

**Fig. 3:**
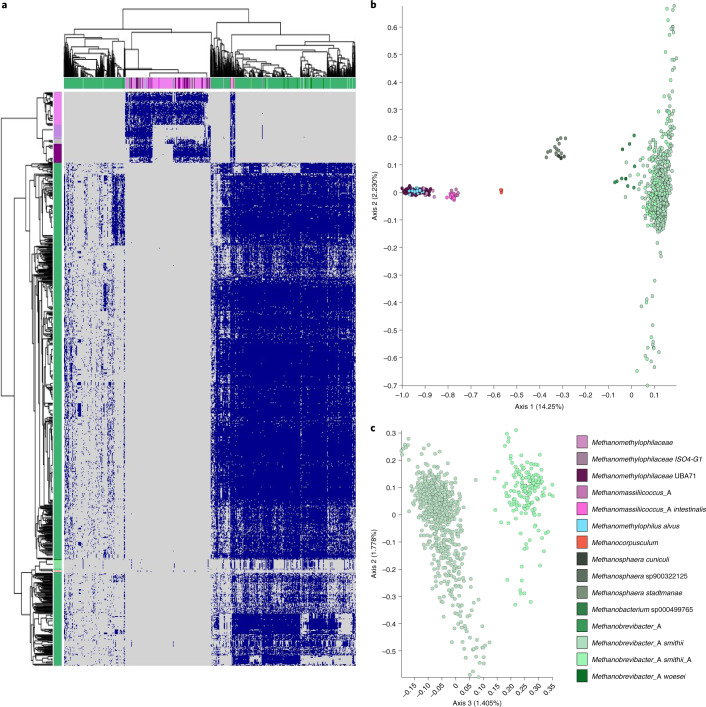
Archaeal genomes from the human gut microbiome distribution and the corresponding unified protein catalogue. **a**, Unified human archaeal protein catalogue based on protein clustering at 50% sequence identity and 80% coverage using MMseqs2 of all 1,167 archaeal genomes. Heatmap depicts the presence of 3,050 proteins (found in >50 genomes; rows) across the 1,167 archaeal genomes (columns). Heatmap visualization was done using the pheatmap library in R. NA, no data available. **b**, The taxonomic distinction of Methanomassiliicoccales, Halobacteriales and Methanobacteriales based on the protein profile (**a**), displayed in a PCoA plot based on Bray–Curtis distances at a depth of 623 archaeal proteins. The PCoA showed five distinct clusters referring to *Methanomethylophilaceae*, *Methanomassiliicoccus*, *Methanocorpusculum*, *Methanosphaera* and *Methanobacteriaceae* spp. **c**, Notably, the clade of *Methanobacteriaceae* sp. was subdivided into *Methanobacterium* sp. and a heterogeneous cluster of *Methanobrevibacter* sp., where *Methanobrevibacter smithii* and *M. smithii*_A (later referred to as *Ca*. *M. intestini,*), form separate clusters.

### The dataset reveals previously undescribed members of the human gastrointestinal archaeome

**Extended Data Fig. 6:**
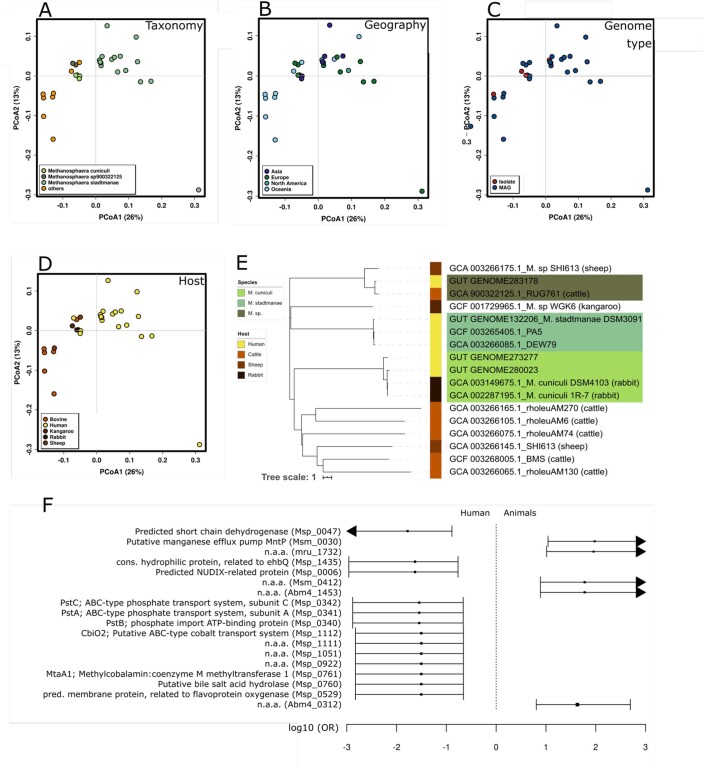
Profiles of human- associated *Methanosphaera* genomes. For comparison, eleven genomes from animal-associated *Methanosphaera* were included. PCoA plots (Bray-Curtis distance) of the genomic profiles according to taxonomy (**a**), geography (**b**), genome type (**c**), and host (**d**) and dendrogram of the genus *Methanosphaera* with human- and animal-associated representatives (**e**). Human-associated species are highlighted in green colors. Colored bar displays the origin: human (yellow) and animals (shades of brown). (**f**): Forest plot showing the outcome of the Wilcoxon rank test comparison of genomes from humans vs. animals (only proteins with FDR < 0.05 are shown), bar displays the odds ratio (OR) (Supplementary Table 7). Arrowheads represent OR that extend beyond the range of the shown X-axis.

We obtained 20 genomes affiliated with *Methanosphaera* sp., including three genomes from isolates. Taxonomically, human-associated *Methanosphaera* genomes were affiliated to three distinct species-level clades (Extended Data Fig. 6 and Supplementary Table 7a–c). Among those, *M. stadtmanae* was the most commonly retrieved, with 17 genomes (14 MAGs). *M. stadtmanae* reads represented a fraction of 0.028% among all microbial reads, with an average fraction of 13.45% among all archaeal reads in reference datasets (for details, see Supplementary Table 2a, and also for other taxa mentioned below). Two MAGs (average nucleotide identity (ANI) 98.5%) clustered within *M. cuniculi*, and were retrieved from healthy Asian subjects living in an urban environment. The *M. cuniculi* type strain was originally isolated from the intestinal tract of a rabbit^28^ and has not been reported thus far in human hosts. One additional MAG belonging to the genus *Methanosphaera* was binned from a gut metagenome of a diseased (colorectal cancer) European male (BMI 21, age 64 years, urban environment). This genome clustered together with *RUG761*, a genome recovered from cattle intestines^29^ (ANI 99.0%; Extended Data Fig. 6).

The dataset of human-associated Methanomassiliicoccales consisted of 145 genomes corresponding to 12 species (Supplementary Table 1a). The genomes were distributed into two families, most of them belonging to ‘host-associated’ Methanomethylophilaceae (116 genomes), the other to Methanomassiliicoccaceae (‘free-living clade’; 29 genomes). Five of the candidate species corresponded to genomes previously found in human samples, comprising 81% of the Methanomassiliicoccales from the present study. These included Methanomassiliicoccales Mx06 sp.^16^ (44 genomes), *Methanomethylophilus alvus*^15^ (37 genomes) and *Methanomassiliicoccus intestinalis*^14^ (20 genomes), being the most prevalent Methanomassiliicoccales representative in human populations^16^. Mx06 representatives were mostly present in young adults (aged 32 years (average), *n* = 34) from rural areas (80%; *n* = 40) in Oceania, Asia and Africa (65%, 13% and 7%, respectively; *n* = 43). Together with its high prevalence (80%) in a population of 7- to 48-year-old uncontacted Amerindians^16,30^, it appears that this species is strongly linked with nonwesternized populations. The young age of people with this species contrasts with previously reported positive correlation between age and methanogen prevalence. Several representatives of this species have the genetic potential to metabolize TMA, a bacterial metabolite involved in trimethylaminuria and suspected in cardiometabolic, cardiovascular and renal diseases. This species is part of a well-supported clade that is separated from other Methanomethylophilaceae genera (*Methanomethylophilus*, *Methanogranum* and *Methanoplasma* spp.) and belongs to the candidate genus ‘UBA71’ following the Genome Taxonomy Database (GTDB) classification (Supplementary Table 1c). We thus suggest that it represents a previously undescribed genus and species, and propose the name of ‘*Candidatus* *Methanoprimaticola hominis*’ *gen. nov., sp. nov*. (Me.tha.no.pri.ma.ti’co.la. N.L. pref. methano- pertaining to methane; N.L. pl. n. Primates a zoological order; L. suff. -cola (from L. masc. or fem. n. incola) an inhabitant, dweller; N.L. fem. n. Methanoprimaticola a methane-forming dweller of primates; ho’mi.nis. L. gen. n. hominis of a human) for representatives of Mx06 (representative MAG: GUT_GENOME268463). *Ca*. *Methanoprimaticola hominis* represented 0.094% of all microbial reads (691 studies), and 0.50–69.22% of all archaeal reads in 48 of 691 analysed studies (Supplementary Table 2a).

In addition to the species previously identified through MAGs or culture approaches, we identified 6 undescribed species of Methanomassiliicoccales, represented by 24 MAGs. One of those gathers 12 MAGs and was more often found among Asian people. We propose naming it ‘*Ca*. *Methanoprimaticola macfarlanii*’ *sp. nov*. (mac.far.la’ne.i. N.L. gen. n. macfarlanei named after George T. Macfarlane; representative MAG: GUT_GENOME251929). This species represented 0.076% of all microbial reads in 691 screened studies (Supplementary Table 2a).

A number of additional archaeal taxa not yet described to be constituents of the human GIT were recovered from the MAG dataset. For details on these and other taxa (*Halorubrum*, *Haloferax*, *Methanocorpuscum* and *Methanobacterium* spp.), please refer to Supplementary Information.

### The *M. smithii* clade splits into two separate species

**Fig. 4:**
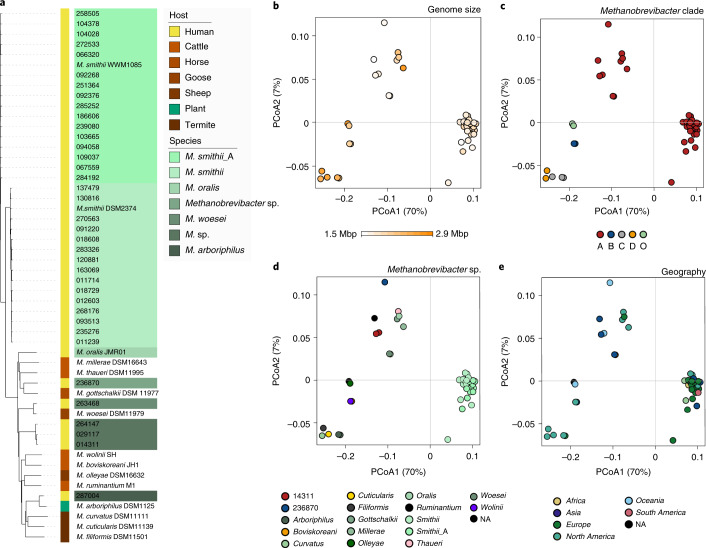
Characteristics of the *Methanobrevibacter* genomes. **a**, Dendrogram of the *Methanobrevibacter* clade based on ANI distance. Twelve representative genomes from sources other than humans were included for comparison (further details are given in Supplementary Table 8). Genomes (strain level) from the human GIT are highlighted in green colours (taxon label). *M. smithii*_A refers to the new species *Ca. M. intestini*. The bar on the left displays the origin: human (yellow bar), animal (shades of red) and plant (green). **b**–**e**, PCoA plots (Bray–Curtis distance) of protein profiles, according to: genome size (**b**), *Methanobrevibacter* clade according to the GTDB (**c**), assigned species (**d**) and geographical origin (**e**). NA, no data available.

An overview on host association, geography, genome size and taxonomic association of known *Methanobrevibacter* spp. and genomes is given in Fig. 4 (for further details on *Methanobrevibacter* genomes besides the *M. smithii* clade, see Supplementary Information).

Based on ANI similarity values, as well as information derived from the protein catalogue, the *M. smithii* group was represented by two species-level clades (tentatively named ‘*smithii*’ and ‘*smithii*_A’ according to the GTDB classification^31^) (Figs. 3c and 4a, and Supplementary Table 8a,b; see also ref. ^25^). *M. smithii*_A was represented in our entire dataset 185 times (16% of the entire dataset), whereas *M. smithii* was detected 797 times (68%), together representing 84% of all genomes in our dataset (Supplementary Table 1a). Based on read mapping, *M. smithii* was found to be responsible for 0.56% of all microbial reads in screened studies, whereas *M. smithii*_A represented 0.13% (Supplementary Table 2a). Together, these two taxa represented 0.69% of all microbial reads (total archaeal reads: 1.21%), confirming their predominance among the gastrointestinal archaea.

The two *M. smithii* groups (sum test, two-sided, genome size corrected by completeness, Supplementary Table 9a) had median genome sizes of 1.7 Mbp for *M. smithii* and 1.8 Mbp for *M. smithii*_A (Supplementary Table 8; genome sizes for isolates: 1.7 Mbp (*M. smithii* DSM2374) and 1.9 Mbp (isolate WWM1085)).

**Extended Data Fig. 7:**
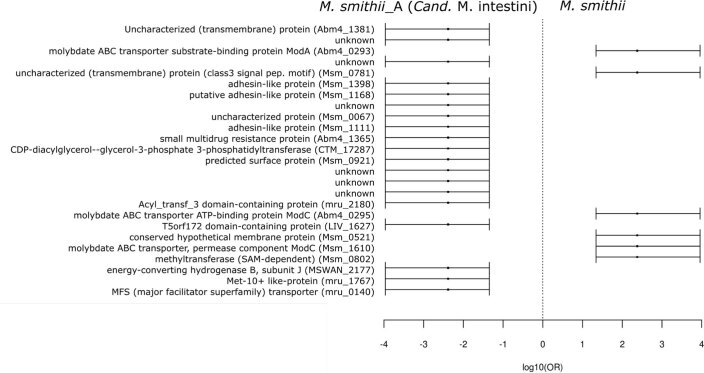
*Methanobrevibacter smithii* Forest plot. Forest plot showing the outcome of the Wilcoxon rank test comparison of the genomic inventory from M. smithii_A (Cand. M. intestini) vs. M. smithii (only TOP 25 proteins are shown; FDR adjusted P<0.000005); bar displays the odds ratio (OR) (see Supplementary Table 9b).

All *M. smithii* strains carried the *modA* gene, which was not detected in any of the *smithii*_A genomes (Supplementary Table 8b). This gene is involved in molybdate transport and responsible for substrate binding^32^. In addition, among the top 25 discriminative proteins (Extended Data Fig. 7 and Supplementary Table 9b), the molybdate ABC transporter permease component, as well as the molybdate ABC transporter ATP-binding protein, were identified in 94% of all *M. smithii* genomes, but in none of the *M. smithii*_A genomes. This indicates a different pathway for molybdate acquisition in the *M. smithii*_A clade. The *M. smithii*_A genomes were further characterized by additional unique membrane/cell-wall-associated proteins, such as adhesin-like proteins, surface proteins and a number of uncharacterized membrane proteins/transporters (Extended Data Fig. 7).

Based on the extent of discriminative features, and an ANI of only 93.95% between the two representative genomes of *M. smithii* and *M. smithii*_A, we propose to rename the *smithii*_A clade, represented by isolate WWM1085 (GUT_GENOME143185 (ref. ^33^)), ‘*Candidatus* *Methanobrevibacter intestini*’ *sp. nov.* (in.tes.ti’ni L. gen. neut. n. *intestini*, of the gut), to further emphasize the presence of two predominant, distinctive *Methanobrevibacter* clades in the GIT. ‘*Ca*. *M. intestini*’ and *M. smithii* cannot be distinguished on 16S ribosomal RNA gene sequences, which is most probably the reason for missing this clade separation previously. However, analysis of the *mcrA* gene revealed a consistent difference between the two clades, with an average of 2.15% difference in amino acid sequence (1.82–2.22%; Supplementary Material 4).

### The human archaeome carries a complex, previously unseen virome

**Extended Data Fig. 8:**
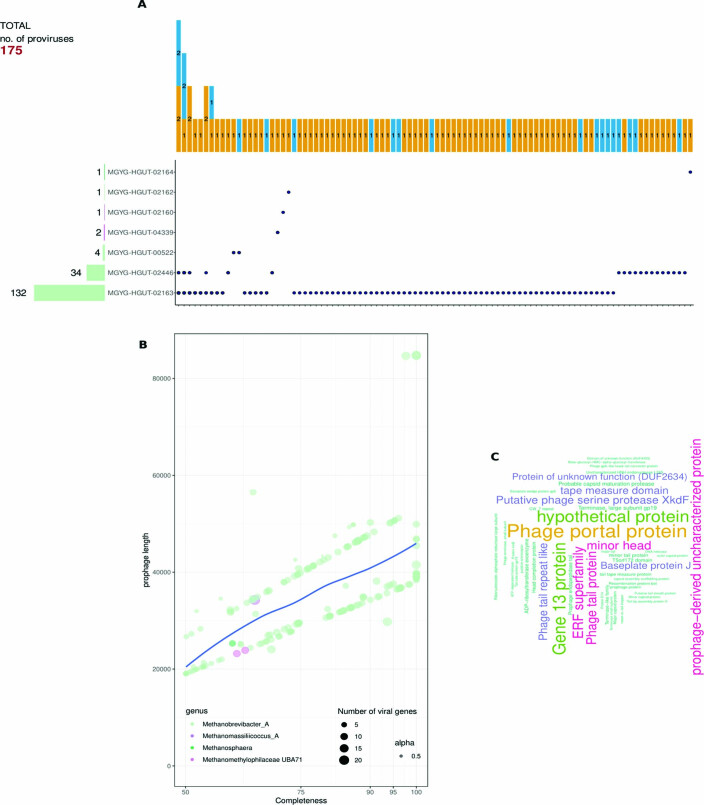
Number of identified MQ and HQ proviruses and confirmed viral genes. **a**) UpSet plot showing the number of viral species color coded by quality (vertical bars, yellow for high-quality; >90% complete, and blue for medium-quality; 50-90% complete), according to the archaeal species where the viral cluster was identified. **b**) Scatter plot representing prophages length vs. estimated completeness color coded by taxonomic assignment at the genus level of host archaeal genome. The size of each data point is relative to the number of identified viral genes per prophage. Locally Weighted Least Squares Regression (LOESS) method used for smoothing. **c**) Word cloud of interesting viral genes identified, where the size of each word is relative to its number of occurrences.

We identified 94 viral populations in our genome datasets (Extended Data Fig. 8 and Supplementary Table 10a–c). Of the identified proviruses, 91 viral species representatives were found to be specific for *Methanobrevibacter A*, and one each for *Methanomassiliicoccus* and *Methanosphaera* spp., and Methanomethylophilaceae UBA71.

Although archaeal viruses in extreme environments were discovered in the early 1970s^34,35^, little is known about nonextremophilic viruses in the highly abundant mesophilic environments, and only a few nonextremophilic archaeal viruses have been isolated so far^36–39^. To the best of our knowledge, no viruses/proviruses have been identified in the past infecting Methanomassiliicoccales and Methanobacteriales members of the human gut.

We explored the uniqueness of these 175 high- and medium-quality proviruses by comparing them with the latest comprehensive human Gut Virome Database (GVD)^3^, and the Viral Refseq Database, using the network-based viral classification tool vConTACT2 (ref. ^40^). However, none of the viruses clustered with any of the sequences in the databases. Due to the lack of similar archaeal viral genomes in the reference databases, the classification and further characterization of discovered archaeal viruses through metagenomic approaches remain challenging.

Taken together, these results reveal that archaeal viruses probably have a currently underestimated diversity and probable ecological importance in the human gut microbiome.

### Human-associated archaea exhibit a lower proportion of bacterial genes than animal-associated archaea

The adaptation of archaea to the GIT may have been favoured by specific acquisition of genes from the resident bacterial community providing additional functions. To assess this possibility, we compared the retrieved *Methanosphaera* and *Methanobrevibacter* genomes with isolates and genomes derived from animal sources (Supplementary Table 11). For this comparison, and to rule out false information from contaminating reads, we used only genomes from isolates and MAGs with 0% contamination.

Human-associated methanogens revealed a significantly lower proportion of genes most probably derived from bacterial origin, irrespective of whether we considered isolates only or both isolates and MAGs. Human-associated *Methanobrevibacter* spp. carried, on average, approximately 2.84% genes annotated as of nonarchaeal origin, which was significantly lower than the proportion of nonarchaeal genes in animal-associated *Methanobrevibacter* sp. (6.09%; Mann–Whitney *U*-test, *P* = 0.00308; genomes from isolates only: 6.36%). This was mainly due to a significantly increased contribution of clostridia-derived genes (specifically from Lachnospiraceae) in genomes from animals (*P* = 0.00116 and *P* < 0.00001, Mann–Whitney *U*-test; Extended Data Fig. 8). Lachnospiraceae representatives are mainly specialized on plant degradation. In particular, *Methanobrevibacter* *smithii*/*smithii*_A (*Ca*. *M. intestini*) representatives revealed a very low contribution of potentially nonarchaeal genes (2.11%; genomes from isolates only: 1.8%).

**Extended Data Fig. 9:**
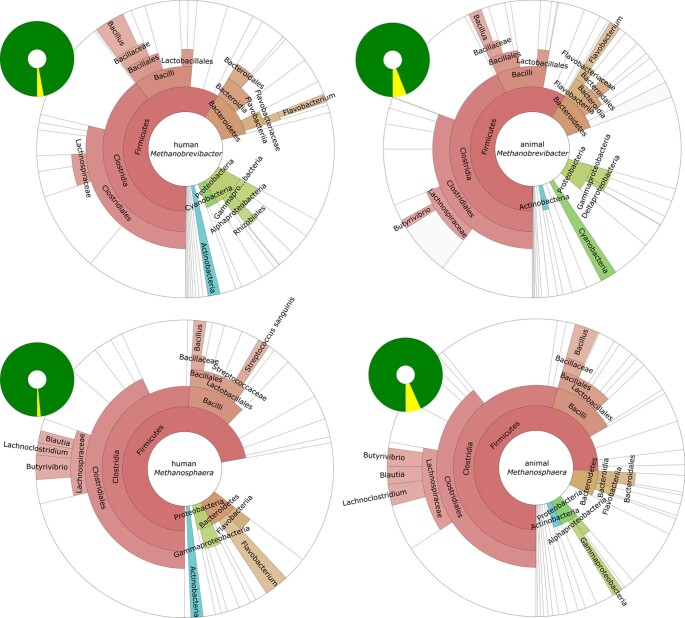
Contribution of bacterial-annotated genes in human- (left) and animal- (right) associated *Methanobrevibacter* and *Methanosphaera* species: Krona chart proportion in percent indicated by the small circles (the yellow wedge refers to proportion of bacterial annota- tion: human *Methanobrevibacter*: 2.84%; animal *Methanobrevibacter*: 6.09%; human *Methanosphaera*: 2.11%; animal *Methanosphaera*: 6.74%) and potential bacterial origin (taxa as displayed in the large circles). Unclassified taxa are whitened out. Only MAGs with 0% contamination and of high quality (taken from ‘strain list’) and genomes from isolates were analyzed (full details are provided in Supplementary Table 11) using eggNOG mapper v2.0.0. Annotated genes were sorted according to their taxonomic affiliation (eggNOG output information: ‘best_tax_level’), and the proportion of archaeal and bacterial genes was calculated.

Human-associated *Methanosphaera* spp. carried on average a proportion of 1.45% of genes of bacterial annotation (genomes from isolates only: 0.68%). Animal-associated *Methanosphaera* spp., however, contained a significantly higher proportion of bacterial genes (6.74%; *P* = 0.00452, Mann–Whitney *U*-test; genomes from animal isolates only: 5.31%). The differences were mainly due to a significantly increased contribution of Bacilli- and Erysipelotrichia-derived genes in genomes from animals (*P* = 0.000441 and 0.000509, respectively; Student’s *t*-test; Extended Data Fig. 9). For information on Methanomassiliicoccales, please refer to Supplementary Information.

Our results indicate that adaptation towards the human host might not necessarily be reflected by a (generally) higher proportion of genes derived from the human gastrointestinal bacteriome.

### Host-associated archaea are distantly related to environmental relatives

We reasoned that host-associated archaea are taxonomically and functionally distant from their environmental relatives due to the characteristics of their individual host environments.

**Fig. 5:**
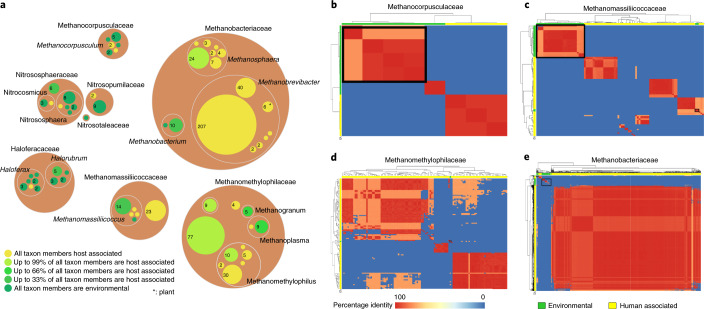
Comparison of host-associated and environmental relatives. **a**, Circle packing plot, displaying the environmental (green) or host-associated (yellow) nature of specific taxa. Analysis was performed on 16S rRNA gene level (Supplementary Table 12). Number of sequences analysed per taxon is indicated by the numbers in the circles and circle size; colours indicate the proportion of host-associated signatures. The largest contribution was observed from *M. smithii* sequences. Note that the yellow colour (‘host associated’) also includes human, animal and plant (*only *M. arboriphilus*)-associated taxa. **b**–**e**, ANI heatmap visualization. ANI analysis based on MinHash sequence mapping was performed using fastANI and visualized using the pheatmap library in R. ANI values represented range from 75% to 80% ANI coloured in light orange, 80–90% ANI in darker orange and over >95% ANI in red. Heatmap for genomes assigned to the taxonomic family of Methanocorpusculaceae (**b**), Methanomassiliicoccaceae (**c**), Methanomethylophilaceae (**d**) and Methanobacteriaceae (**e**). Genomes isolated from the human gut microbiome (labelling on the *x* and *y* axes in yellow) can be separated from the genomes isolated from the environment (labelling on the *x* and *y* axes in green; Supplementary Table 12). Environmental archaeal genome clustering is marked with a black square.

In 16S rRNA gene-based analyses (Supplementary Table 12a,b), we found that members of genera *Methanobrevibacter* and *Methanosphaera*, as well as *Ca*. *Methanomethylophilus* belonged almost exclusively to taxa from host-associated (animal, human, plant) sources, whereas *Methanocorpusculum* and *Nitrososphaeria* spp., and Haloferaceae were more related to environmental strains (Fig. 5a).

ANI-based analyses of the families Methanobacteriaceae, Methanocorpusculaceae, Methanomethylophilaceae and Methanomassiliicoccaceae revealed an overall clear separation between the MAGs of different origins (Fig. 5b–e; additional details in Supplementary Information). Based on the information on their respective biomes, the archaeal strains of the present study can be classified into three groups: (1) exclusively found in the human gut, (2) host (human, animal, plant) associated and (3) widespread in the environment, with the first two groups representing the highest proportion^5,7,9^. Following this classification and based on the current availability of genomes and metadata, *H. massiliensis*, *M. oralis*, *M. smithii*, *M. smithii_A* (*Ca*. *M. intestini*), *M. stadtmanae*, *M. intestinalis* and *M. alvus* can be considered to be affiliated to group (1). Species belonging to group (2) include *M. woesei* and *M. cuniculi*. Species of group (3) are represented by *H. lipolyticum*^41^, *M. arboriphilus*^42,43^ and *M. luminyensis*^13,16^, widespread in various environments.

### Functional and metabolic interaction of the archaeome with the gut environment

We analysed specific features that could indicate the advanced interaction of the human-associated GIT archaea with their gut environment (host and nonarchaeal microbiome; Fig. 1).

Loss of genes involved in dealing with oxidative stress is considered to be a trait of host association, because environmental strains have to face nonpermanently, strict anaerobic conditions, whereas this is not the case for strains inhabiting the GIT. We therefore analysed the presence of genes associated with oxygen resistance (catalase, superoxide dismutase, peroxiredoxin, rubredoxin and thioredoxin^44^). Catalase was detected in some Methanomassiliicoccales (mainly *Methanomassiliicoccus* representatives) and Haloarchaea, and in *Methanobrevibacter arboriphilus* and *Methanobacterium* spp. The presence of a superoxide dismutase was rarely detected, namely in members of *Haloferax* and *Halorubrum* spp. None of the *Methanobrevibacter* representatives, except *M. arboriphilus*, carried the peroxiredoxin gene. In contrast, thioredoxin and rubredoxin were detected in most of the genomes (Fig. 1).

**Extended Data Fig. 10:**
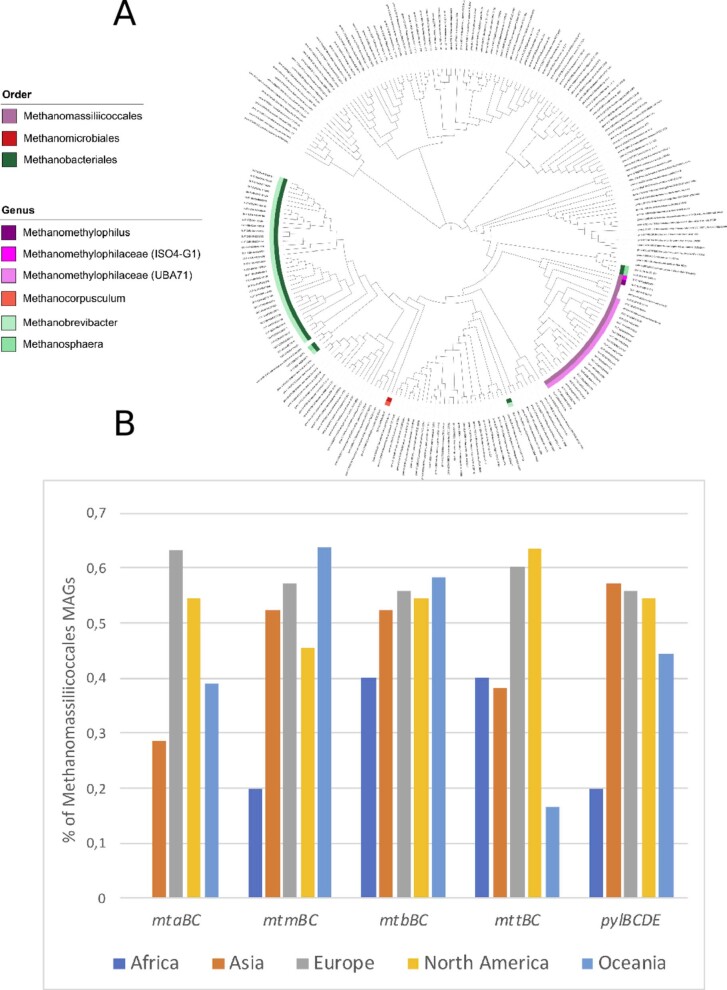
Functional and metabolic interaction of the archaeome with the gut environment. **a**) Archaeal bile salt hydrolase genes (this study) integrated in the bacterial tree of BSHs^18^. Archaeal genes are highlighted by the colored ring, indicating the respective taxonomic affiliation. **b**) Geographic distribution of methyl-compound utilization capacity by Methanomasiliicoccales representatives. The presence of mtaBC, mtmBC, mtbBC and mttBC genes needed for methanol, monomethylamine, dimethylamine and, trimethylamine utilization, respectively, as well as pylBCDE genes responsible for the biosynthesis of pyrrolysine (an aminoacid specifically present in methylamine methyltransferases) was searched in all Methanomassiliicoccales. MAGs. Methanomassiliicoccales were separated according to the geographic location (continents) of their host, and the percentage of them having the above mentioned genes is displayed. Average Methanomassiliicoccales MAGs completeness are Africa, 70.1%; Asia, 72.6%; Europe, 79.9%; Oceania, 87.2%.

Additional functions of interest are adhesins and bile salt hydrolases (that is, choloylglycine hydrolase (CGH)). Adhesins or adhesin-like proteins were widely observed (Fig. 1). CGH homologues were detected in 11 of 27 of the archaeal species, including the 5 most prevalent ones (*M. smithii*, *‘Ca*. *M. intestini*’, *M. stadtmanae*, *M. alvus* and ‘*Ca*. *M. hominis*’). CGH genes were not detected in any of the *Methanomassiliicoccus* genomes and in the Haloferaceae, indicating their importance for specialization towards the human gut. It should be noted that the CGH genes detected in Methanomassiliicoccales, Methanomicrobiales and Methanobacteriales formed separate clusters within the bacterial bile salt hydrolases gene tree (Extended Data Fig. 10), indicating their potential acquisition from different events of horizontal gene transfers (HGTs).

**Fig. 6:**
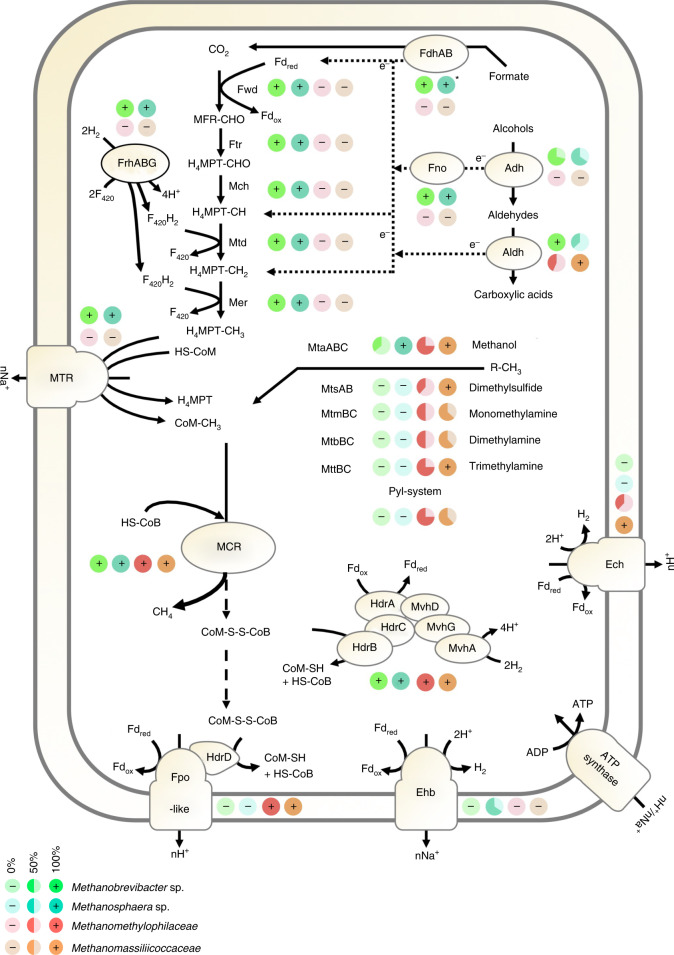
Methanogenic pathways in 23 human gut-associated Methanobacteriales and Methanomassiliicoccales. The proportion of species with a given protein or protein complex is indicated by pie charts for *Methanobrevibacter* sp. (*n* = 7), *Methanosphaera* sp. (*n* = 3), Methanomethylophilaceae (*n* = 8) and Methanomassiliicoccus (*n* = 5). For clarity, the nature of the electron transporter and some intermediate steps in the electron transfers are not displayed for formate and alcohol utilization. R-CH_3_ corresponds to methanol, dimethylsulfide, monomethylamine, dimethylamine or TMA. Alcohol could be ethanol or secondary alcohols. The absence of certain enzymes may be due to incompleteness of MAGs. MFR, methanofuran; H4MPT, tetrahydromethanopterin; -CHO, formyl group; -CH, methenyl group; -CH_2_, methylene group; Fd_ox_/Fd_red_ oxidized/reduced ferredoxin; HS-CoM, coenzyme M; HS-CoB, coenzyme B; CoM-S-S-CoB, heterodisulfide; e^-^, electrons (without mentioning the transporter).

Additional adaptations were observed at the metabolism level. Apart from key components of methanogenesis, methyl-coenzyme M reductase (MCR) and heterodisulfide reductase/[NiFe] hydrogenase (Hdr/Mvh) complexes, the main gut methanogens (Methanobacteriales and Methanomassiliicoccales) possess very distinct methanogenesis pathways (Fig. 6 and Supplementary Table 13). For example, different from all Methanomassiliicoccales, all human gut *Methanobrevibacter* spp. have the genetic potential for formate and H_2_/CO_2_ utilization. However, 83% of all methanogenic MAGs (including Methanobacteriales and Methanomassiliicoccales) have the *mtaABC* genes, providing the genetic potential to use methanol. The two dominant *Methanobrevibacter* spp. carry *mtaABC* genes, whereas four species that are rarely present do not carry these genes, strongly suggesting that methanol utilization might provide a selective advantage in the human gut. However, the condition under which *Methanobrevibacter* sp. uses methanol and whether it is a methanogenic substrate or enters an anabolic pathway remains to be elucidated.

The two dominant *Methanobrevibacter* sp. also display the genetic potential to use alcohols (probably secondary alcohols and ethanol) as electron donors for methanogenesis. One of the *Methanosphaera* spp. may also have the genetic capacity to reduce methanol with ethanol for methanogenesis as described earlier^45^, but this species was encountered only once in our analyses, and *M. stadtmanae* cannot perform this pathway.

The majority (11/13) of the GIT-associated species of Methanomassiliicoccales code for the MttBC methyltransferase and corrinoid protein needed for methanogenesis from TMA. This capacity would allow them to decrease the concentrations of this molecule produced by gut microbiota and involved in cardiovascular diseases^16,21^. The presence of the *mttBC* genes was detected in a larger proportion of the Methanomassiliicoccales MAGs originating from Europe and North America (~60%) with respect to Africa and Asia (~40%) or Oceania (17%) (Extended Data Fig. 10). These variations may reflect different TMA-production capacity by bacteria in the microbiota across these populations and diet habits. One of the two species of Methanomethylophilaceae lacking TMA-utilization capacity (*Ca*. *Methanoprimatia macfarlanii*) also lacks MtbBC and MtmBC methyltransferases and corrinoid proteins for dimethylamine and monomethylamine utilization, respectively. However, several strains of this species have the genes encoding the synthesis of pyrrolysine (*pylSBCD*), a proteinogenic amino acid (UAG codon encoded) quite exclusive to methylamine-specific methyltransferases^46,47^. The absence of detection of the methylamine-specific methyltransferases in these MAGs, including MttBC for TMA utilization, is thus probably due to genome incompleteness. The other species lacking methylamine methyltransferase, corresponding to Methanomassiliicoccales Mx02 (ref. ^16^), also lack any other genes known to be involved in methyl-compound utilization or in any alternative methanogenesis pathways (Supplementary Table 13). The absence of these methanogenesis genes in all the MAGs of Methanomassiliicoccales Mx02 and in previously obtained related MAGs, support assumptions^16,48^ on the presence of unknown methanogenesis pathways probably based on unknown methyltransferases, or another metabolic route in the Methanomassiliicoccales. Thus, we propose the name ‘*Candidatus* *Methanarcanum hacksteinii*’ Mx02 *gen. nov*., *sp. nov.* (Me.than.ar.ca’num. N.L. neut. n. methanum methane; L. masc. adj. arcanus silent, secret; N.L. neut. n. Methanarcanum; an archaeon-forming methane in a puzzling way; hack.stei’ni.i. N.L. gen. n. hacksteinii named after Johannes H. P. Hackstein; representative MAG: GUT_GENOME287001).

## Discussion

Our work adds original information on the biology of the GIT archaeome, by characterizing a collection of 1,167 nonredundant archaeal genomes. We were able to make initial associations between the diversity of gut-associated archaea with several demographic and geographic patterns. However, many geographic locations remain undersampled to date.

As our genome collection is based on public datasets processed for the analysis of the bacterial component of the microbiome, a large number of archaeal species requiring specialized methods for cell lysis and DNA extraction^6^ may be missing. Moreover, sequencing stool samples is not necessarily representative of the complete diversity of species in the intestines, because some archaea have been shown to form biofilms and stick to the epithelium^49^. Besides, as several taxa in our collection are represented by only single genome representatives, additional conspecific strains will be needed to allow profound analyses. Thus, we are far from capturing the entire diversity of the GIT archaeome.

The overall observed percentage of archaea present in the human gut microbiomes (~1.2%, Supplementary Table 2a) is in agreement with recently reported average percentages based on 16S rRNA gene and shotgun metagenomic information^18^. The abundance of methanogenic archaea in the human gut is highly variable and represented by two physiological types of humans, namely methane emitters (>5 p.p.m. methane in breath, ~20% of the western population; 2% archaeal signatures in overall microbial GIT community) and nonemitters (0.002% archaeal signatures), exhaling negligible amounts of this gas. The effects of these striking differences of high- and low-methane emitters on host physiology are largely unclear to date, but are considered to be relevant to health and disease^18^.

The presented genome collection and the catalogue of 1.8 million putative proteins can now serve as a unique source to generate hypotheses to be addressed in future studies. This includes aspects on: (1) the archaeal physiology and metabolism; (2) the detailed comparison and differentiation of free-living, animal- and human host-associated archaea (see also ref. ^50,51^), including the aspect of HGT; (3) the interaction with the bacterial microbiome and the virome; and (4) the type of archaeal cross-talk with the human host. Moreover, considering that only 9 of 27 archaeal species detected in the human gut metagenomes had a cultured representative, the provided resource can serve as a starting point for targeted cultivation of previously uncultivated members of the archaeome and their virome.

Due to missing metadata and limited statistical power, it is challenging to establish significant associations between the archaeal genomic diversity and human lifestyles or diseases herein. Thus, experimentally driven, well-designed studies will ultimately elucidate the impact of archaea on human health^9^. Moreover, incorporating both transcriptomics and proteomics data will further reinforce the genomic predictions and improve our understanding of the regulation of archaeal physiology and host adaptation. Future efforts should also seek to extend the dataset beyond the gastrointestinal environment, to other human body sites and hosts.

Overall, our work contributes substantially to the understanding of the microbiome of the human GIT as a complex multi-domain bacterial, archaeal, fungal and viral network^52–56^. All microbial puzzle pieces have co-evolved and adapted together within the gut ecosystem, so study of these dynamic multi-kingdom interactions holistically will provide crucial insights into the role of the gut microbiome in health.

## Methods

A resource summary is provided in Supplementary Table 14.

### Dataset description

To explore the diversity of archaea in human gastrointestinal samples, we compiled publicly available genomes from four recent collections of MAGs^2,25–27,57^. Briefly, the Unified Human Gastrointestinal Genome (UHGG) collection (data access June 2020, https://www.ebi.ac.uk/metagenomics/genomes) holds published, nonredundant MAGs and isolates, collected from public repositories and associated metadata information (see ref. ^2^ for more details). No statistical methods were used to predetermine sample sizes. We additionally included published genomes from cultured archaea available in the National Center for Biotechnology Information (NCBI)^58^, Pathosystems Resource Integration Center (PATRIC)^59^ and Integrated Microbial Genomes and Microbiomes (IMG/M)^60^ repositories.

Genomes were compared using Mash v.2.1 (ref. ^61^) and, for genomes that were estimated to be identical and had a Mash distance of 0, only one was selected. In addition, we included genomes of ‘*Ca*. *Methanomethylophilus alvus’*^15^ and ‘*Ca*. *Methanomassiliicoccus intestinalis’*^14^, as well as human gut-derived MAGs of Methanomassiliicoccales Mx02, Mx03 and Mx06, and additional ‘*Ca. M. intestinalis*’^16^, and the human isolate *Methanobrevibacter arboriphilus* ANOR1 (ref. ^42^) to complete the dataset. Those genomes were assigned a genome accession no. (GUT_GENOME286998, GUT_GENOME287001, GUT_GENOME287002, GUT_GENOME287004), as given in Supplementary Table 1a. This brought the total number of genomes used for the analysis in the present study to 1,167. Data collection and analysis were not performed blind to the conditions of the experiments.

### Genome quality and taxonomic classification

The completeness of the nonredundant 1,167 genomes was evaluated by CheckM v.1.0.11 (ref. ^62^) and only genomes that were >50% complete and had <5% contamination were selected (following the protocol from ref. ^2^; Extended Data Figs. 1 and 2a–c). This procedure yielded 1,167 nonchimeric^63^ (clade separation score (CSS) = 0; Supplementary Table 1a) and nonredundant archaeal genomes (Mash distance threshold of 0.001, 99.9% ANI^61^; Supplementary Table 1a) which were further subgrouped into individual strains (<99% ANI similarity, >75% genome completeness; Supplementary Table 1b; 98 genomes; Fig. 1), and species (<95% ANI similarity, >75% genome completeness; Supplementary Table 1c; 27 genomes). For this, the best quality genome (genome completeness, minimal contamination, strain heterogeneity and assembly continuity based on the N50 value) from each cluster was selected as representative or, whenever an isolate was available, it was preferred and used for further analysis.

Read mapping was performed with Bowtie2 (ref. ^64^) for the genomes that had original raw reads available and were post-processed using samtools^65^. Strain heterogeneity within each MAG was computed using the script ‘polymut.py’ from the CMseq tool (https://github.com/SegataLab/cmseq). Alignment files were used together with the parameters --minqual 30 and --cov 10, following the method description in refs. ^2,25^. A threshold of ≤0.5% indicates heterogeneity of assembly and the higher likelihood of one strain present per assembly. GUNC^63^ was used to detect chimerism in all 1,167 genomes and resulted in a CSS of 0 for all genomes (Supplementary Table 1e). A CSS closer to a value of 0 indicates that a genome is free of contamination and all genes are assigned to the same taxonomy, whereas a CSS score closer to 1 indicates chimerism. The CSS, taken together with the contamination thresholds from CheckM, demonstrated that our 1,167 genomes were not chimeric in nature.

DRep v.2.0.0 (ref. ^66^) was used to dereplicate the complete dataset at 95% and 99% ANI values. The 95% ANI values were selected to separate between species boundaries (*n* = 27)^67^. A cut-off of 99% was selected for strain delineation, provided that a stable number of clusters for MAGs >75% complete had <5% contamination (*n* = 98; Extended Data Fig. 3a). Lower thresholds did not affect the number of strains recovered. The resulting strain and species representatives are given in Supplementary Table 1a-c.

All genomes were taxonomically annotated following the procedure given in ref. ^2^. The taxonomic assignment was performed using the GTDB Toolkit v.0.3.1 (database release 04-RS89)^68^ and default parameters that utilize a set of 122 marker genes to identify archaeal MAGs. Previously undescribed species and genera were defined when no taxonomic information was assigned for all members of a species cluster and their species representatives based on the GTDB database. The methodology is detailed in Supplementary Fig. 1.

### Genome annotation and protein catalogue

Protein-coding sequences (CDSs) were predicted and annotated with Prokka v.1.14.5 (ref. ^69^) using the parameters ‘--kingdom Archaea’ to include nonfragmented archaea-curated proteins from the UniProtKB database and ‘--rfam’ to scan for noncoding RNAs. CDSs were further characterized using eggNOG-mapper v.2.0.0 (ref. ^70^) and the eggNOG database v.5.0 (ref. ^71^), which includes the latest release of all archaeal clusters of orthologous groups and their proteins^72^.

The protein catalogue was generated by combining all predicted CDSs (total number 1,790,493) derived from the 1,167 nonredundant archaeal genomes. MMseqs2 linclust^73^ was used to cluster the concatenated proteins dataset using the options ‘--cov-mode 1 -c 0.8’ (minimum coverage threshold of 80% the length of the shortest sequence) and ‘--kmer-per-seq 80’. Proteins were clustered at different percentage identities and the number of unique proteins resulting per clustering for each taxonomic family was computed and visualized (Extended Data Fig. 3b). To reduce the risk of contaminants, the proteins were filtered to remove all nonclustered proteins. This gave a total of 28,581 proteins clustering at 50% identity (Supplementary Material 1) visualized using the library pheatmap^74^ in R. MMseqs2 using the ‘easy-search’ was additionally used for aligning the 28,581 proteins to UniRef 50 (ref. ^75^) (date of download January 2021) to verify predicted proteins that resulted in 13,254 (46.37%) proteins with a hit.

In addition to the protein catalogue, the various species and strain subsets of the total 1,167 archaeal genomes (Supplementary Table 1b,c) were submitted to MaGe MicroScope (Microbial Genome Annotation & Analysis Platform^76^), for detailed analyses of genomic synteny, and the detection of bile salt hydrolases, oxygen resistance genes and adhesins, following the automated annotation of MaGe (Supplementary Table 1f).

### Relative abundance of archaea in human metagenomes

Raw read datasets (691) were obtained from studies of the human gut microbiome, out of which 691 (of 1,167) medium- or high-quality archaeal MAGs were assembled. The remainder was not made public by their original submitters (Supplementary Table 1a).

We mapped raw reads to the 27 reference archaeal species representatives using Bowtie2-align^64^ and post-processed using samtools^65^. The generated sorted mapping files were used to calculate the breadth of coverage. Breadth of coverage was calculated by dividing the total number of bases covered (using samtools mpileup) by the length of the reference genome. To get the percentage coverage breadth we multiplied the resulting number by 100.

For measuring the relative abundance of the 27 archaeal species in the different metagenomics datasets we used CoverM (https://github.com/wwood/CoverM) and the relative_abundance calculation method (Supplementary Table 2f).

Reads were additionally mapped using Kraken v.2.1.2 (ref. ^77^) (with default settings) against (1) a custom database of the UHGG catalogue available from the MGnify FTP site (http://ftp.ebi.ac.uk/pub/databases/metagenomics/mgnify_genomes/human-gut/v1.0/uhgg_kraken2-db) and (2) a customized database of the 27 archaeal species representatives in our dataset because we supplemented the initial resource with additional isolates (Data description). Results were processed using Bracken v.2.5.3 (ref. ^78^) using both read lengths 100 and 250 to estimate the relative abundance of domain-, family- and species-level taxa (Supplementary Table 2b–d). We did not observe differences in the output values of the analysis between read lengths 100 and 250.

### Protein abundance estimation

To avoid estimations based on potential false negatives derived from sample processing or genome binning, all raw reads were aligned on the unified archaeal protein catalogue using DIAMOND BLASTx^79^. The hits were counted and the result was transformed into a matrix of the number of hits for each protein per study using the pandas library^80^. This resulted in a mapped protein matrix used for further statistical analysis to minimize the risk for sample or batch effects in our dataset (Supplementary Material 2).

Besides genomic information (genome length, number of contigs, N50, GC content, genome completeness, genome contamination, and number of rRNAs and transfer RNAs), 11 metadata categories (numerical 2, categorical 9) could be considered for the dataset. Information about the geographic origin was available for 1,063 genomes (91% of the dataset covered countries from maximum to minimum: the USA, Israel, Spain, Sweden, Fiji, UK, Austria, Denmark, the Netherlands, France, China, Peru, Germany, Madagascar, United Republic of Tanzania, Australia, Canada, Ireland, Italy, Russia, El Salvador, Iceland, Mongolia, Norway, on five continents; Supplementary Table 1d and Fig. 2a).

Information on lifestyle was available for 1,054 genomes (90%, max.–min.: urban, rural, semi-urban), health state (healthy, diseased) for 894 genomes (77%), age group (adult, elderly person, child, teenager, infant) for 825 genomes (71%), gender (female, male) for 620 genomes (53%), BMI group (normal weight, overweight, obesity class 1, underweight, obesity class 2, extreme obesity class 3) for 505 genomes (43%) and name of disease (colorectal cancer, infection, type 2 diabetes, adenoma, obesity, ulcerative colitis, nonalcoholic fatty liver disease (NAFLD), Parkinson’s disease, ankylosing spondylitis—arthritis, faecal microbiota transplantation (FMT), cirrhosis) for 303 genomes (26%) and treatment (antibiotics) for 241 genomes (21%). However, most genomes (third quartile, 75% of all values) were obtained from healthy women of normal weight, living in urban areas of Europe (Fig. 2).

To overcome biases introduced by potential residual MAGs contamination issues, we focused our analyses on patterns observed in two or more genomes, unless stated otherwise. In addition, we explored protein diversity patterns and their functional characterization among isolated genomes to corroborate those observed in MAGs. Finally, to avoid estimations based on potential false negatives derived from sample processing or genome binning, raw reads were mapped on the unified archaeal protein catalogue (Supplementary Material 1) as a reference to generate a mapped protein matrix (Supplementary Material 2), which minimized the risk for sample or batch effects in our dataset.

Supervised classification and regressions with RandomForest were applied to predict respective metadata categories from the unified archaeal protein catalogue and the mapped protein matrix with the q2-sample-classifier plugin^81^. To reduce the risk of overfitting, the matrices were downsampled to a minimum of 50 genomes for each tested metadata category, as recommended by scikit-learn 0.24.1 (ref. ^82^). First subsets of each metadata category were created from the entire protein matrix and randomly split into a training set and a test set with the proportions 80%:20%. By using K-fold cross-validation, the training set served as a learning model to predict class probabilities with settings for optimized feature selection and parameter tuning. In the end, model accuracy was determined by comparing the predicted values between the training and test datasets.

### Pan-genome analysis

Pan-genome analysis was performed using Panaroo^83^ in ‘strict’ mode because it accounts for potential annotation errors, fragment assemblies and contaminated genomes to recover an accurate pan-genome. Pan-genome analysis was performed for archaeal genomes of the same families and the same genus. We used Heaps’ law (η = κ × *n* − α) to estimate whether we had an open or a closed pan-genome^84^, This analysis was carried out in the R package ‘micropan’^85^ using a default permutation value of 100, where η is the predicted number of genes for a particular number of genomes (*n*), and κ (intercept parameter) and α (decay parameter) are the constants used to fit the curve after the genomes have been ordered in a random way. An open pan-genome is indicated by α < 1 whereas a closed pan-genome is indicated by α > 1.

### Estimation of growth rates

Growth rates were estimated using GRiD^86^ in the multiplex mode (minimum coverage = 1 and reassignment of ambiguous reads) by a customized GRiD database based on the created subset of high-quality archaeal genomes on species level. As the original raw reads were not available for each representative genome and the remaining read sets were not made publically available, growth rate estimates covered 131 metagenomic read sets (70% of all archaeal genomes grouped at strain level).

### In-depth taxonomic and clustering analyses of the various genera

ANI distances and tree matrices were calculated using the online resources of the enveomics platform^87^, MaGe^76^, as well as Microbial Genomes Atlas (MiGA)^88^. Dendrograms, built on the ANI tree matrix, were annotated using the iTOL tool (Interactive Tree Of Life)^89^, and processed using InkScape. For specific considerations involving additional genomes from animals, a subselection of the archaeal genomes was reanalysed together with the additional genomes following the same settings as described for the protein catalogue procedures above (respective datasets are given in the Supplementary Table 12).

*McrA* genes were extracted via MaGe, hosting all strain-level genomes (Supplementary Material 4). *McrA* genes were aligned using MegaX^90^, and a maximum likelihood tree was calculated (default settings).

Bacterial and archaeal *BSH* genes were derived from ref. ^91^ and supplemented with *BSH* genes from genomes in the present study. Sequences were cropped and a tree was calculated using the MEGA-X Maximum Likelihood Phylogeny Reconstruction. The tree was annotated using the iTOL tool^89^.

### Initial HGT analysis

Representative genomes from isolates and MAGs with 0% contamination according to CheckM results were selected for these analyses (*Methanosphaera* spp.: 8 from humans, 7 from animals; *Methanobrevibacter* spp.: 30 from humans, 11 from animals). A list with full details is provided in Supplementary Table 11. Genomes from animals were obtained from NCBI (ncbi.nlm.nih.go/genome), representing all available high-quality genomes (isolates, MAGs) of the respective genus at the time point of analysis (2020; Supplementary Table 11). The selected genomes were further characterized as previously mentioned using eggNOG-mapper v.2.0.0 (ref. ^70^) and the previously mentioned databases (Genome annotation and protein catalogue). Annotated genes were sorted according to their taxonomic affiliation (eggNOG output information: ‘best_tax_level’), and the proportion of archaeal and bacterial genes was calculated for all genomes and genera. Data were visualized using Krona^92^.

### Detection of virulence and resistance genes

To predict potential virulence genes in all 1,167 archaeal genomes, ABRicate v.0.5 (https://github.com/tseemann/abricate) was used to profile the following databases: CARD^93^, Resfinder^94^, PlasmidFinder^95^, ARG-ANNOT^96^, EcOH^97^ and MEGARes 2.0 (ref. ^98^), as well as NCBI AMRFinderPlus^99^. As ABRicate is solely based on DNA sequences, blastX searches using DIAMOND^79^ was used to complement results from ABRicate on the level of protein sequences in the virulence factor database (VFDB v.20191122)^100,101^ and CARD together with the Resistance Gene Identifier^102^.

Specific groups of proteins and genes involved in human interaction were investigated according to available annotations from MaGe^76^ and eggNOG-mapper^70^.

### Viral identification, quality estimation and comparisons to viral databases

To assess the presence of prophages, VirSorter2 (ref. ^103^) was used to scan all MAGs. CheckV^104^ was used to estimate completeness and assess the quality of VirSorter2-predicted viruses. To ensure that we overcame possible contamination issues that could potentially result from the binning process, we selected proviruses flanked within archaeal contigs for this analysis. VirSorter2 tends to overestimate provirus boundaries (https://github.com/jiarong/VirSorter2), therefore CheckV is recommended to apply a quality control check and remove false positives. CheckV looks for host–virus boundaries based on differences in GC content and gene annotation in a sliding window approach. Proviruses (detected by VirSorter2 followed by CheckV and CheckV on a separate run) that had a quality assignment of medium quality (50–90% completeness) of high quality (>90% completeness), or were complete, were considered for further analysis. Quality assignments by CheckV are based on Minimum Information about an Uncultivated Virus (MIUViG) standards^105^. It is worth mentioning that proviruses detected by Virsorter2 followed by CheckV were detected by running CheckV independently. The selected proviruses were subsequently clustered with MMseqs2 using the ‘linclust’ function with the same parameters previously specified and MMseqs function ‘result2repseq’ to select a viral cluster representative.

We identified 94 viral populations in our genome datasets. This number is the result of clustering 45 high-quality (>90% completeness) and 130 medium-quality (50–90% completeness) archaeal proviruses, flanked within archaeal contigs, at 95% identity and 80% coverage, where one to a maximum of two proviruses were identified per host. The selected cut-off is commonly used for viral species^3,105–110^ definition (Extended Data Fig. 8 and Supplementary Table 10a–c).

Open reading frames of viral populations with the previously specified MIUViG quality were used as input for vConTACT2 (ref. ^40^) including Viral RefSeq genome (v.97). VconTACT2 is used to affiliate a family or a genus rank group to viral populations and thus to determine taxonomic diversity.

A recent study was published by Gregory et al.^3^ where a human GVD harbours 33,242 viral populations, including 0.1% archaeal viruses resulting from 2,697 gut metagenomes in 32 studies. This dataset was used as a reference database to scan the identified viral scaffolds using MMseqs2 ‘easy-linclust’ function at 50, 80, 90 and 95% identity.

### Comparison to environmental archaea

For considerations based on 16S rRNA genes, 16S rRNA genes of representative genomes were extracted using Metaxa2 (ref. ^111^) (*n* = 314; not all 16S rRNA genes could be recovered). This dataset was supplemented with data from amplicon sequencing studies and clone sequences from archaeal signatures from human gastrointestinal samples (dataset described in ref. ^9^; *n* = 381 in total). These sequences were aligned and classified using the SILVA rRNA database^112^. More specifically, the retrieved 16S rRNA genes were subjected to the ACT tool (alignment, classification and tree service)^113^, using the following parameters: basic alignment parameters: ‘removed’; search and classify, minimum identity with query sequence: ‘0.95’; number of neighbours per query sequence: ‘10’; compute tree; workflow: ‘Denovo including neighbours’ and default parameters; and advanced tree computation parameters, positional variability filter: ‘none’, domain: ‘archaea’. Unclassified sequences were removed from the dataset. Via SILVA SINA, ten next neighbours were selected, and information on their isolation source was gathered through NCBI (Supplementary Material 3 and Supplementary Table 12a; the final dataset contained 566 sequences). Grouping was performed at the genus/species level, and information on the percentage of host-associated archaea in all groups was displayed as a circle packing plot (RawGraphs online tool, https://app.rawgraphs.io).

For genome-based analyses, a set of 623 archaeal MAGs identified from environmental and gastrointestinal samples (for example, rumen, guinea-pigs and baboon faeces) was used as a reference dataset for comparison to the set of archaea isolated from the human gut microbiome^2,114^. All environmental genomes used were >50% complete, and also up to 90% complete, with <5% contamination as well. To estimate the pairwise ANI distance between environmental archaeal genome dataset (Supplementary Table 12) and the archaeal genomes from the human gut microbiome, we used fastANI^67^, a tool that effectively discriminated intra- and interspecies boundaries for >90,000 prokaryotic genomes.

### Metabolic interaction of the archaeome with the gastrointestinal environment

Proteins involved in methanogenesis were searched in all genomes using customized Hidden Markov Model profiles (threshold e-value 10^−5^) implemented in Macsyfinder^115^. This allowed us to determine the presence of enzymatic complexes on the basis of the presence of all or most subunits. The presence in the 26 methanogenic species was first evaluated based on the representative genome (which are the most complete/less contaminated). If most of the MAGs in a species have an enzyme, then this enzyme was considered to be present in the species, even if absent from the best representative genome.

### Functional interaction of the archaeome with the gastrointestinal environment

Specific functions were searched for (‘search by keywords’-function) in MaGe^76^. Presence and absence information was used for tree annotation through iTOL^89^. The backbone tree was based on ANI similarity as described above.

### Tools used for data visualization

Principal coordinate analyses (PCoAs) and other graphic displays based on the unified archaeal protein matrix were calculated and visualized in Qiime2 (ref. ^116^) and Calypso^117^. Venn diagrams were created with creately (https://creately.com). Alluvial plots, circle packing plots and contour plots were generated with RAWGraphs (https://app.rawgraphs.io). Strip charts were created with Calypso. Dendrograms, based on the ANI tree matrix, were annotated using the iTOL tool. All figure panels were created using InkScape.

### Quantification and statistical analysis

All statistical analyses were conducted using R, Qiime2^116^, Calypso^117^ and MaAsLin2 (ref. ^118^). Where applicable, data distribution was tested using Shapiro–Wilk normality tests. Statistical significance was determined by nonparametric tests including Spearman’s rank correlations, PERMANOVA and Wilcoxon’s rank-sum tests for pairwise analysis, Mann–Whitney *U*-tests for unpaired data and Kruskal–Wallis tests if the significance had to be determined for all groups. Significance was considered at an α < 0.05 after 999 permutations. *P* values were corrected for multi-hypothesis testing using the false discovery rate. To control for potential batch effects resulting from different isolation methods, DNA extraction protocols, assembly methods and/or sampling depth, etc., the study accession was set as a random effect in MaAsLin2 analysis. In addition, linear mixed effect models^81^ were calculated to test whether Bray–Curtis distances and α diversity (Shannon’s diversity index) of the mapped archaeal protein matrix changed over age, BMI, genome completeness or growth rate (GRiD), and in response to the use of antibiotics, geography (continent or country), disease, sex, health status or lifestyle in the dataset.

### Reporting Summary

Further information on research design is available in the Nature Research Reporting Summary linked to this article.

## Supplementary information

Supplementary Information Supplementary Results, Tables and Material.

Reporting Summary

Peer Review Information

Supplementary Data 1 Unified human archaeal protein catalogue based on clustering at 50% identity of all genome CDSs, with associated lineage and genome information, and a summary of the number of genes shared per archaeal family (basis for Fig. 2a–c).

Supplementary Data 2 DIAMOND BLASTx output of reads alignment on the unified human archaeal protein catalogue. This mapped protein matrix was the basis for linear mixed effect models, MaAsLin2 analysis and metadata predictions shown in Extended Data Fig. 5.

Supplementary Data 3 Alignment of the *mcrA* genes of *M. smithii* and *M. smithii*_A, fasta file.

Supplementary Data 4 Environmental and human archaea 16S rRNA genes, fasta file (basis for Fig. 5a).

Supplementary Tables Supplementary Tables 1–14.

## Data Availability

All the recovered genomes are available for bulk download in an archived folder ‘archaea_gut-genomes.tar.gz’ in generic feature format at http://ftp.ebi.ac.uk/pub/databases/metagenomics/genome_sets. All used genomes and metagenomes in the present study are publicly available on NCBI and MGnify resource. Accession no. details and paper references of used genomes and metagenomes are summarized in Supplementary Table 1a–f.

## Extended data

are available for this paper at 10.1038/s41564-021-01020-9.

## Code availability

The present study did not generate code, and mentioned tools used for the data analysis were applied with default parameters unless specified otherwise.
