# Publisher Correction: A catalogue of 1,167 genomes from the human gut archaeome

**DOI:** 10.1038/s41564-022-01061-8

**Published:** 2022-01-10

**Authors:** Cynthia Maria Chibani, Alexander Mahnert, Guillaume Borrel, Alexandre Almeida, Almut Werner, Jean-François Brugère, Simonetta Gribaldo, Robert D. Finn, Ruth A. Schmitz, Christine Moissl-Eichinger

**Affiliations:** 1 Institute for Microbiology, Christian-Albrechts-University Kiel, Kiel, Germany; 2 Diagnostic & Research Institute of Hygiene, Microbiology and Environmental Medicine, Medical University Graz, Graz, Austria; 3 Department of Microbiology, Unit of Evolutionary Biology of the Microbial Cell, Institut Pasteur, Paris, France; 4 European Molecular Biology Laboratory, European Bioinformatics Institute, Cambridge, UK; 5 Wellcome Sanger Institute, Cambridge, UK; 6 Institut Universitaire de Technologie Clermont Auvergne, Université Clermont Auvergne, CNRS, UMR 6023 Laboratoire Microorganismes: Genome et Environnement, Clermont-Ferrand, France; 7 BioTechMed, Graz, Austria

**Keywords:** Archaea, Microbial genetics

Correction to: *Nature Microbiology* 10.1038/s41564-021-01020-9, published online 30 December.

In the version of this article initially published, the images for Figs. 2 and 3 were in the wrong order, although figure callouts, titles and captions were correct. The images have now been reordered in the online version the article.

