# Supplementary material for: A catalogue of 1,167 genomes from the human gut archaeome
Source: Nat Microbiol. 2021 Dec 30;7(1):48–61. doi: 10.1038/s41564-021-01020-9 (PMC8727293; doi:10.1038/s41564-021-01020-9)
Supplement: Supplementary file 1 — Supplementary Results, Tables and Material. [file 41564_2021_1020_MOESM1_ESM.pdf]

nature portfolio

https://doi.org/10.1038/s41564-021-01020-9

Supplementary information

A catalogue of 1,167 genomes from the human gut archaeome

In the format provided by the authors and unedited

Supporting Information

A catalogue of 1,167 genomes from the human gut archaeome

Cynthia Maria Chibani[1,8], Alexander Mahnert[2,8], Guillaume Borrel[3], Alexandre Almeida[4,5], Almut Werner[1], Jean-François Brugère[6], Simonetta Gribaldo[3], Robert D. Finn[4], Ruth A. Schmitz[1,9], Christine Moissl-Eichinger[2,7,9]

[1] Institute for Microbiology, Christian-Albrechts-University Kiel, Kiel, Germany

[2] Diagnostic & Research Institute of Hygiene, Microbiology and Environmental Medicine, Medical University Graz, Neue Stiftingtalstraße 6, 8010 Graz, Austria

[3] Department of Microbiology, Unit Evolutionary Biology of the Microbial Cell, Institut Pasteur, Paris, France

[4] European Molecular Biology Laboratory, European Bioinformatics Institute (EMBL-EBI), Wellcome Genome Campus, Hinxton, Cambridge, United Kingdom.

[5] Wellcome Sanger Institute, Wellcome Genome Campus, Hinxton, Cambridge, United Kingdom.

[6] Institut Universitaire de Technologie Clermont Auvergne, Université Clermont Auvergne, CNRS, UMR 6023 Laboratoire Microorganismes: Genome et Environnement, Clermont-Ferrand, France

[7] BioTechMed, Graz, Austria

[8] These authors contributed equally

[9] Lead contact

[*] Correspondence: christine.moissl-eichinger@medunigraz.at, rschmitz@ifam.uni-kiel.de

This file contains:

- Supplementary Results
- Description of Supplementary Tables and Material

Supplementary Results

The human archaeome is actively replicating

In order to address the question on whether the human gastrointestinal archaea are actively replicating, we measured the growth rate of each archaeal genome using the Growth Rate Index (GRiD)[1]. Based on GRiD analysis from 131 metagenomic data resources, 35% of the dataset was shown to be actively replicating at the time point of sampling with a mean growth rate index of 1.2+/-0.1. Growth rate indices differed between *Methanomassiliicoccus_A, Methanobrevibacter smithii_A* (*Ca.* M. intestini)*, M. smithii,* Methanomethylophilaceae UBA71 clade, and *Methanocorpusculum.* The highest growth rate was observed for *Methanomassiliicoccus_A*, the lowest was observed for the *Methanocorpusculum* representatives. Growth rates were significantly different between *Methanobrevibacter smithii* and. *Methanobrevibacter smithii_A* (*Cand.* M. intestini) (pairwise Wilcoxon rank sum test; p=0.009). Furthermore, predicted growth rates were also significantly associated with a subject's health status or lifestyle (see below for analysis of linear mixed effect models on metadata categories) (Table S6). Nevertheless, growth rate index estimates could be biased towards fast growing organisms or multi-fork replications[2]. Hence, direct comparisons might be misleading and assumptions on relative proportions might be less error prone.

The genetic diversity of the gut archaeome still remains undersampled

To understand the level of sampling completeness of the human archaeome, we computed the genetic diversity for genera and families that had over ten genome members (details in Table S1a). We found that more genomes are needed to have a saturated overview of the genetic diversity of the genera *Methanobrevibacter* (995 genomes, α=0.72), and *Methanomassiliicoccus* (27 genomes, α=0.96) (indicated by an α <1 which means each added genome contributed new genes). However, additional species of the genera Methanomethylophilaceae UBA71 clade (66 genomes, α=1.1), *Methanosphaera* (29 genomes, α=1.31), and *Methanomethylophilus* (38 genomes, α=1.54) would not contribute new genes[3]. Members of the *Haloferax*, *Halorubrum*, *Methanobacterium*, *Methanocorpusculum,* Methanomethylophilaceae ISO4-G1 and Methanomethylophilaceae unassigned genera do not have enough members yet and therefore remain undersampled.

Observations were further confirmed by analysis at family level. The three archaeal families Methanomassiliicoccaceae (α=0.61), Methanobacteriaceae (α=0.66) and Methanomethylophilacea (α=0.54) have an open pan genome indicated by an α <1. Members of the Haloferacaceae and Methanocorpusculaceae families remain undersampled due to low members.

Overall, pan-genome analysis revealed that the gut archaeome remains undersampled, especially since there are not enough members of some archaeal genera and families to assess diversity.

However, due to the nature of MAGs, it is important to sequence more genomes of the species that are found scarcely in the dataset in order to assess the true genetic diversity in the gastrointestinal tract (GIT).

Archaeal protein profile correlates with geographic and demographic parameters: additional results

According to MaAsLin2 analysis, numerous hypothetical proteins showed significant associations with various metadata categories. For a few proteins with functional annotation, significant associations of e.g. adenylosuccinate synthetase, NADPH dehydrogenase, vitamin B12 import ATP-binding protein BtuD, Na(+)-translocating NADH-quinone reductase subunit F, or L-rhamnose 1-dehydrogenase (NADP(+)) were observed for a healthy phenotype (Table S5).

Combinatory effects of multiple metadata categories were tested with linear mixed effect models run on the mapped protein matrix to elucidate significant changes of genome distance (Bray-Curtis distance along PCoA axis 1) and diversity (Shannon entropy H') along subject's age, BMI, or genome growth indices (Table S6). This analysis revealed significant changes (negative proportional) for genome distance and diversity along a subject's BMI who did not receive antibiotics or in response to an urban lifestyle. Furthermore, genome distances showed significant changes along a subject's age or genome growth indices in response to a healthy phenotype and for the latter as well for an urban lifestyle.

The dataset reveals previously undescribed members of the human gastrointestinal archaeome particularly in Asian and Oceanian populations: additional results

We identified one MAG affiliated to *Halorubrum lipolyticum* (Halobacteriaceae, GUT_GENOME103718, Fig. 1), which showed 100% ANI similarity to the type strain originally isolated from a Chinese lake[5] . Together with isolate *Haloferax massiliensis*, which was retrieved from human feces in 2018 [6], they represent the only two genomes affiliated to halophilic archaea available from the human GIT. Although a high prevalence of haloarchaea was previously reported in Asian cohorts[7], none of the two genomes in our study showed abundance based on read mappings of 691 metagenomes datasets (Table S2a). In addition, none of the two genomes revealed obvious adaptations towards the human gut ecosystem, such as bile salt hydrolases. We thus conclude that haloarchaea might be transient residents of the human GIT.

Two genomes affiliated to Methanocorpusculaceae (Methanomicrobiales) (Fig. 1; ANI 98.6%), representing two strains and one species, were retrieved from subjects from the Fiji islands (Fig. 1; Fig. 2). Closest relatives were *M. parvum* (Strain XII, type strain; ANI 70.32%) and *M. bavaricum*

(DSM4179; 70.25%), which were originally isolated from digester and wastewater environments, respectively. We were able to classify 0.158% of archaeal reads as *Methanocorpusculaceae* species GUT_GENOME23783 and to show that its relative abundance ranged between 2.8 and 24% in 4 out of 691 studies (Table S2a).

Signatures of the genus *Methanobacterium* have been frequently detected in amplicon-based archaeome studies using small intestine biopsies or samples from oral cavities, but have neither been isolated nor detected by genome-centric analyses[8,910]. The presence of *Methanobacterium* species in the human GIT have been confirmed in our study, as one Methanobacterium MAG (GUT_GENOME283701) was obtained from a European male (age 65; colorectal cancer), with a genome size of 1.9 Mbp (98.13% completeness; 0% contamination). Based on ANI values, the closest described relative was *Methanobacterium formicicum* DSM3637 (87.75%), a well-known, formate consuming methanogen in ruminants[11]. Formate consuming genetic capacity was as well confirmed for the *Methanobacterium* MAG identified in our study. Methanobacterium was found not to be very abundant in screened studies, as only 0.002% of all microbial reads were assigned to it. However, based on read recruitment, 0.2% of archaeal reads were classified as *Methanobacterium sp000499765*, and relative abundance based on coverage calculation was 72.92% in the same 1 out of 691 studies with the accession ERR479173 out of which this genome was initially assembled (Table S2a). We can conclude that *Methanobacterium sp000499765* might potentially be transiently associated with the human GIT.

The *Methanobrevibacter smithii* clade splits into two separate species: additional results

Several previously undescribed genomes were detected within the *Methanobrevibacter* clade, including the cluster containing GUT_GENOME014311 (Fig. 1, Fig. 4A). This cluster was represented by seven genomes (95% ANI cut-off), clustering into two distinctive strains and one species. All seven genomes were originally binned from samples obtained from Asia (China) and Oceania (Fiji) only; genomes were obtained from healthy and diseased, rural and urban subjects. The closest relatives identified were *Methanobrevibacter woesei* GCA 003111605T (71.54% ANI) and *Methanobrevibacter smithii* ATCC 35061 NC 009515T. Additional *Methanobrevibacter* genomes were recovered with larger distance to known isolates, such as GUT_GENOME236870 and GUT_GENOME237437 (99.67% ANI; both isolated from a Fiji cohort; Fig. 1), with a genome similarity of 84.13% to *M. gottschalkii* DSM11977 (ANI), or GUT_GENOME237054 (also from a Fiji cohort), showing a similarity of 78.11% to *M. gottschalkii* (ANI).

Interestingly, we did not recover any MAGs belonging to *Methanobrevibacter oralis*. Although this species is a member of the human oral microbiome, it has been isolated at least once from human stool [12]. For further details on the relative abundance on the different *Methanobrevibacter* species please refer to Table S2a.

Human-associated archaea exhibit a lower proportion of bacterial genes than animal-associated archaea: additional results

An extraordinary high contribution of bacterial genes were observed for Methanomassiliicoccales, revealing a proportion of archaeal-annotated genes of 74.96% only (mean of 9 MAGs (0% contamination, high quality genomes; strain list) and 2 isolates). The proportion ranged from 65.72-96.88% in the MAGs, the genomes from isolates revealed an archaeal-gene annotation of 67.35 and 69.65%. This might reflect however an incomplete annotation of the Methanomassiliicoccales genomes based on the low number of available genomes and isolates.

In all cases (Methanobrevibacter, Methanosphaera, Methanomassiliicoccales), the largest contribution was observed from Firmicutes (Clostridia and bacilli; example bile salt hydrolases see main text) with a lower contribution of Bacteroidetes, and Proteobacteria. Consistently those clades represent the most abundant bacterial microbiome components, increasing the probability for HGT towards members of the archaeome[13] .

Host-associated archaea are distantly related from environmental relatives: additional results

ANI-based analyses of the families Methanobacteriaceae, Methanocorpusculaceae, Methanomethylophilaceae and Methanomassiliicoccaceae revealed an overall clear separation between the MAGs of different origins (Fig. 5, B-E). More specifically, for Methanomassiliicoccaceae (Fig. 5C), we observed that the two gut genomes classified as *M. luminyensis* (GUT_GENOME132203, GUT_GENOME140888) cluster within an environmental archaeal MAGs clade whose members are taxonomically classified as uncultured *Methanomassiliicoccus*; however, both human-derived genomes share a higher ANI value compared to the genomes of the sister clade. Concerning Methanobacteriaceae (Fig. 5E), GCA_002509095.1 (Methanobacteriaceae archaeon UBA254), and GUT_GENOME283178 (*Methanosphaera* sp900322125) shared an ANI value of 96.09% while every other pairwise comparison between human and environmental archaeal MAG had an ANI value less than 88.09%. Based on taxonomic classification resolved at species level, most species identified in the human gut are indeed host-associated (reviewed in [15–17]).

It should be noted that for certain clades only a limited number of genomes is currently available, and thus their host/environmental tropism remains to be precisely determined (e.g. Methanomethylophilus sp001481295, *Methanosphaera* sp900322125, *Methanobacterium* sp000499765, *H. massiliensis*, *Methanomassiliicoccus)*. Notably, in both types of analyses, the genus *Methanobrevibacter* was overwhelmingly affiliated with the human and other hosts. This is a unique trait amongst all analyzed archaeal genera (Fig. 5A).

Description of Supplementary Tables and Material

Table S1. Detailed description of all MAGs and isolates, which were analyzed in this study. This table includes the list of all 1167 archaeal genomes, a strain list (99% ANI distance), a species list (95% ANI distance) and all available metadata. Table S1 includes all quality metrics, raw reads accession numbers and corresponding references. These data sets were the basis for Fig. 1, Fig. 2 and Fig. S2. Table S1a: Detailed description of all MAGs and isolates. Table S1b: Detailed description of strain list. Table S1c: Detailed description of species list. Table S1d: Associated metadata with links to genome and raw read data. Metadata. Table S1e: GUNC output. Table S1f: Genome identifiers in MaGe.

Table S2. Prevalence and abundance of archaeal lineages in the human GIT. Read-based community profiler output based on UHGG catalog and the 27 archaeal species representatives of the human gut. The relative abundance of archaea (Table S2a) was estimated at the domain (Table S2b), family (Table S2c) and species-level taxa (Table S2c). Relative abundance of each species representative in all studies where raw data was available based on read mapping and breadth of coverage computation (Table S2e,f).

Table S3. Predictive accuracy of metadata categories based on the unified protein catalogue. Basis for Fig. S4.

Table S4. Predictive accuracy of metadata categories based on the mapped protein matrix. Basis for Fig. S5.

Table S5. Multivariate Association with Linear Models (MaAsLin2) of the mapped protein matrix.

Table S6. Linear mixed effect models (LME) based on Shannon entropy and Bray-Curtis distances of the mapped protein matrix.

Table S7. Metadata information on the human and animal *Methanosphaera* analysis dataset (Table S7a), protein catalogue information for those genomes (Table S7b), and Wilcoxon rank test comparison (Table S7c). Basis for Fig. S6.

Table S8. Metadata information on the human and animal *Methanobrevibacter* analysis dataset (Table S8a), and protein catalogue information for those genomes (Table S8b) (cut-off: presence in at least 10 genomes). Basis for Fig. 4.

Table S9. Comparison of *M. smithii* vs. *M. smithii*_A, based on genome size (Table S9a) and discriminative proteins (Table S9b; Wilcoxon top 25). Basis for information provided in chapter "The *Methanobrevibacter smithii* clade splits into two separate species".

Table S10. Identified proviruses (Table S10a) and their viral genes (Table S10b) clustering into viral populations including quality summaries (Table S10c) and host where they were identified from. Basis for Fig. S8.

Table S11. Proportions of non-archaeal proteins for Methanosphaera, Methanobrevibacter and Methanomassiliicoccales, organized for human and animal-derived genomes, and isolates. Basis for Fig. S9.

Table S12. Comparison to environmental archaea. Origin of 16S rRNA genes. Basis for Fig. 6A (Table S11a). Subset of the environmental archaeal genomes identified by Parks et al. 2017. Basis for Fig. 5 B,C,D and E (Table S11b).

Table S13. Presence/absence of enzymatic complexes and pathways in the 27 species reported in this study. Basis for Fig. 6.

Table S14. Resource summary table on genome data, raw reads, software and databases used in this research.

Supplementary Material 1. Unified human archaeal protein catalogue based on clustering at 50% identity of all genome CDS, with associated lineage and genome information and a summary of the number of genes shared per archaeal family. Basis for Fig. 3A, C, D and E.

Supplementary Material 2. DIAMOND BLASTx output of reads alignment on the Unified human archaeal protein catalogue. This mapped protein matrix was the basis for linear mixed effect models, MaAsLin2 analysis and metadata predictions shown in Fig. S5.

Supplementary Material 3. Alignment of the mcrA genes of M. smithii and M. smithii_A.

Supplementary Material 4. Environmental and human archaea 16S rRNA genes, basis for Fig. 5A.

References for Supplementary Information

1. Emiola, A. & Oh, J. High throughput in situ metagenomic measurement of bacterial replication at ultra-low sequencing coverage. *Nat. Commun.* **9**, 1–8 (2018).
2. Long, A. M., Hou, S., Ignacio-Espinoza, J. C. & Fuhrman, J. A. Benchmarking microbial growth rate predictions from metagenomes. *ISME J.* **15**, 183–195 (2021).
3. Tettelin, H., Riley, D., Cattuto, C. & Medini, D. Comparative genomics: the bacterial pan-genome. *Curr. Opin. Microbiol.* **11**, 472–477 (2008).
4. Lex, A., Gehlenborg, N., Strobelt, H., Vuillemot, R. & Pfister, H. UpSet: visualization of intersecting sets. *IEEE Trans. Vis. Comput. Graph.* **20**, 1983–1992 (2014).
5. Cui, H.-L., Tohty, D., Zhou, P.-J. & Liu, S.-J. Halorubrum lipolyticum sp. nov. and Halorubrum aidingense sp. nov., isolated from two salt lakes in Xin-Jiang, China. *Int. J. Syst. Evol. Microbiol.* **56**, 1631–1634 (2006).
6. Khelaifia, S. *et al.* Genome sequence and description of Haloferax massiliense sp. nov., a new halophilic archaeon isolated from the human gut. *Extremophiles* **22**, 485–498 (2018).
7. Kim, J. Y. *et al.* The human gut archaeome: identification of diverse haloarchaea in Korean subjects. *Microbiome* **8**, 1–17 (2020).
8. Matarazzo, F., Ribeiro, A. C., Feres, M., Faveri, M. & Mayer, M. P. A. Diversity and quantitative analysis of Archaea in aggressive periodontitis and periodontally healthy subjects. *J. Clin. Periodontol.* **38**, 621–627 (2011).
9. Koskinen, K. *et al.* First insights into the diverse human archaeome: Specific detection of Archaea in the gastrointestinal tract, lung, and nose and on skin. *MBio* **8**, (2017).
10. Faveri, M. *et al.* Prevalence and microbiological diversity of Archaea in peri-implantitis subjects by 16S ribosomal RNA clonal analysis. *J. Periodontal Res.* **46**, 338–344 (2011).
11. Chellapandi, P., Bharathi, M., Sangavai, C. & Prathiviraj, R. Methanobacterium formicicum as a target rumen methanogen for the development of new methane mitigation interventions: A review. *Vet. Anim. Sci.* **6**, 86–94 (2018).
12. Khelaifia, S., Garibal, M., Robert, C., Raoult, D. & Drancourt, M. Draft genome sequencing of Methanobrevibacter oralis strain JMR01, isolated from the human intestinal microbiota. *Genome Announc.* **2**, e00073-14 (2014).
13. Lurie-Weinberger, M. N., Peeri, M., Tuller, T. & Gophna, U. Extensive inter-domain lateral gene transfer in the evolution of the human commensal Methanosphaera stadtmanae. *Front. Genet.* **3**, 182 (2012).
14. Huerta-Cepas, J. *et al.* Fast genome-wide functional annotation through orthology assignment by eggNOG-mapper. *Mol. Biol. Evol.* **34**, 2115–2122 (2017).
15. Moissl-Eichinger, C. *et al.* Archaea Are Interactive Components of Complex Microbiomes. *Trends Microbiol.* **26**, (2018).
16. Bang, C. & Schmitz, R. A. Archaea: forgotten players in the microbiome. *Emerg. Top. Life Sci.* ETLS20180035 (2018).
17. Borrel, G., Brugère, J. F., Gribaldo, S., Schmitz, R. A. & Moissl-Eichinger, C. The host-associated archaeome. *Nature Reviews Microbiology* **18**, 622–636 (2020).
18. Song, Z. *et al.* Taxonomic profiling and populational patterns of bacterial bile salt hydrolase (BSH) genes based on worldwide human gut microbiome. *Microbiome* **7**, 1–16 (2019).

References for Supplementary Tables

- Asnicar, Francesco, Serena Manara, Moreno Zolfo, Duy Tin Truong, Matthias Scholz, Federica Armanini, Pamela Ferretti, et al. 2017. "Studying Vertical Microbiome Transmission from Mothers to Infants by Strain-Level Metagenomic Profiling." MSystems 2 (1). https://doi.org/10.1128/mSystems.00164-16.
- Bäckhed, Fredrik, Josefine Roswall, Yangqing Peng, Qiang Feng, Huijue Jia, Petia Kovatcheva-Datchary, Yin Li, et al. 2015. "Dynamics and Stabilization of the Human Gut Microbiome during the First Year of Life." Cell Host & Microbe 17 (5): 690–703. https://doi.org/10.1016/j.chom.2015.04.004.
- Bedarf, J. R., F. Hildebrand, L. P. Coelho, S. Sunagawa, M. Bahram, F. Goeser, P. Bork, and U. Wüllner. 2017. "Functional Implications of Microbial and Viral Gut Metagenome Changes in Early Stage L-DOPA-Naïve Parkinson's Disease Patients." *Genome Medicine* 9 (1): 39. https://doi.org/10.1186/s13073-017-0428-y.
- Bengtsson-Palme, Johan, Martin Angelin, Mikael Huss, Sanela Kjellqvist, Erik Kristiansson, Helena Palmgren, D. G. Joakim Larsson, and Anders Johansson. 2015. "The Human Gut Microbiome as a Transporter of Antibiotic Resistance Genes between Continents." *Antimicrobial Agents and Chemotherapy* 59 (10): 6551–60. https://doi.org/10.1128/AAC.00933-15.
- Cleary, Brian, Ilana Lauren Brito, Katherine Huang, Dirk Gevers, Terrance Shea, Sarah Young, and Eric J. Alm. 2015. "Detection of Low-Abundance Bacterial Strains in Metagenomic Datasets by Eigengenome Partitioning." *Nature Biotechnology* 33 (10): 1053–60. https://doi.org/10.1038/nbt.3329.
- Costea, Paul I, Luis Pedro Coelho, Shinichi Sunagawa, Robin Munch, Jaime Huerta-Cepas, Kristoffer Forslund, Falk Hildebrand, Almagul Kushugulova, Georg Zeller, and Peer Bork. 2017. "Subspecies in the Global Human Gut Microbiome." Molecular Systems Biology 13 (12): 960. https://doi.org/10.15252/msb.20177589.
- Feng, Qiang, Suisha Liang, Huijue Jia, Andreas Stadlmayr, Longqing Tang, Zhou Lan, Dongya Zhang, et al. 2015. "Gut Microbiome Development along the Colorectal Adenoma–Carcinoma Sequence." Nature Communications 6 (1): 6528. https://doi.org/10.1038/ncomms7528.
- Franzosa, Eric A., Xochitl C. Morgan, Nicola Segata, Levi Waldron, Joshua Reyes, Ashlee M. Earl, Georgia Giannoukos, et al. 2014. "Relating the Metatranscriptome and Metagenome of the Human Gut." Proceedings of the National Academy of Sciences 111 (22): E2329–38. https://doi.org/10.1073/pnas.1319284111.
- Gu, Yanyun, Xiaokai Wang, Junhua Li, Yifei Zhang, Huanzi Zhong, Ruixin Liu, Dongya Zhang, et al. 2017. "Analyses of Gut Microbiota and Plasma Bile Acids Enable Stratification of Patients for Antidiabetic Treatment." Nature Communications 8 (1): 1785. https://doi.org/10.1038/s41467-017-01682-2.
- He, Qing, Yuan Gao, Zhuye Jie, Xinlei Yu, Janne Marie Laursen, Liang Xiao, Ying Li, et al. 2017. "Two Distinct Metacommunities Characterize the Gut Microbiota in Crohn's Disease Patients." GigaScience 6 (7). https://doi.org/10.1093/gigascience/gix050.
- Karlsson, Fredrik H., Frida Fåk, Intawat Nookaew, Valentina Tremaroli, Björn Fagerberg, Dina Petranovic, Fredrik Bäckhed, and Jens Nielsen. 2012. "Symptomatic Atherosclerosis Is Associated with an Altered Gut Metagenome." Nature Communications 3 (1): 1245. https://doi.org/10.1038/ncomms2266.
- Li, Junhua, Huijue Jia, Xianghang Cai, Huanzi Zhong, Qiang Feng, Shinichi Sunagawa, Manimozhiyan Arumugam, et al. 2014. "An Integrated Catalog of Reference Genes in the Human Gut Microbiome." Nature Biotechnology 32 (8): 834–41. https://doi.org/10.1038/nbt.2942.
- Liu, Wenjun, Jiachao Zhang, Chunyan Wu, Shunfeng Cai, Weiqiang Huang, Jing Chen, Xiaoxia Xi, et al. 2016. "Unique Features of Ethnic Mongolian Gut Microbiome Revealed by Metagenomic Analysis." Scientific Reports 6 (1): 34826. https://doi.org/10.1038/srep34826.
- Lloyd-Price, Jason, Anup Mahurkar, Gholamali Rahnavard, Jonathan Crabtree, Joshua Orvis, A. Brantley Hall, Arthur Brady, et al. 2017. "Strains, Functions and Dynamics in the Expanded Human Microbiome Project." Nature 550 (7674): 61–66. https://doi.org/10.1038/nature23889.
- Loomba, Rohit, Victor Seguritan, Weizhong Li, Tao Long, Niels Klitgord, Archana Bhatt, Parambir Singh Dulai, et al. 2017. "Gut Microbiome-Based Metagenomic Signature for Non-Invasive Detection of Advanced Fibrosis in Human Nonalcoholic Fatty Liver Disease." Cell Metabolism 25 (5): 1054-1062.e5. https://doi.org/10.1016/j.cmet.2017.04.001.
- Mehta, Raaj S., Galeb S. Abu-Ali, David A. Drew, Jason Lloyd-Price, Ayshwarya Subramanian, Paul Lochhead, Amit D. Joshi, et al. 2018. "Stability of the Human Faecal Microbiome in a Cohort of Adult Men." Nature Microbiology 3 (3): 347–55. https://doi.org/10.1038/s41564-017-0096-0.
- Nielsen, H. Bjørn, Mathieu Almeida, Agnieszka Sierakowska Juncker, Simon Rasmussen, Junhua Li, Shinichi Sunagawa, Damian R. Plichta, et al. 2014. "Identification and Assembly of Genomes and Genetic Elements in Complex Metagenomic Samples without Using Reference Genomes." Nature Biotechnology 32 (8): 822–28. https://doi.org/10.1038/nbt.2939.
- Obregon-Tito, Alexandra J., Raul Y. Tito, Jessica Metcalf, Krithivasan Sankaranarayanan, Jose C. Clemente, Luke K. Ursell, Zhenjiang Zech Xu, et al. 2015. "Subsistence Strategies in Traditional Societies Distinguish Gut Microbiomes." Nature Communications 6 (1): 6505. https://doi.org/10.1038/ncomms7505.
- Palleja, Albert, Alireza Kashani, Kristine H. Allin, Trine Nielsen, Chenchen Zhang, Yin Li, Thorsten Brach, et al. 2016. "Roux-En-Y Gastric Bypass Surgery of Morbidly Obese Patients Induces Swift and Persistent Changes of the Individual Gut Microbiota." Genome Medicine 8 (1): 67. https://doi.org/10.1186/s13073-016-0312-1.

- Pehrsson, Erica C., Pablo Tsukayama, Sanket Patel, Melissa Mejía-Bautista, Giordano Sosa-Soto, Karla M. Navarrete, Maritza Calderon, et al. 2016. “Interconnected Microbiomes and Resistomes in Low-Income Human Habitats.” Nature 533 (7602): 212–16. https://doi.org/10.1038/nature17672.
- Petersen, Lauren M., Eddy J. Bautista, Hoan Nguyen, Blake M. Hanson, Lei Chen, Sai H. Lek, Erica Sodergren, and George M. Weinstock. 2017. “Community Characteristics of the Gut Microbiomes of Competitive Cyclists.” Microbiome 5 (1): 98. https://doi.org/10.1186/s40168-017-0320-4.
- Qin, Junjie, Ruiqiang Li, Jeroen Raes, Manimozhiyan Arumugam, Kristoffer Solvsten Burgdorf, Chaysavanh Manichanh, Trine Nielsen, et al. 2010. “A Human Gut Microbial Gene Catalogue Established by Metagenomic Sequencing.” Nature 464 (7285): 59–65. https://doi.org/10.1038/nature08821.
- Qin, Junjie, Yingrui Li, Zhiming Cai, Shenghui Li, Jianfeng Zhu, Fan Zhang, Suisha Liang, et al. 2012. “A Metagenome-Wide Association Study of Gut Microbiota in Type 2 Diabetes.” Nature 490 (7418): 55–60. https://doi.org/10.1038/nature11450.
- Rampelli, Simone, Stephanie L. Schnorr, Clarissa Consolandi, Silvia Turroni, Marco Severgnini, Clelia Peano, Patrizia Brigidi, Alyssa N. Crittenden, Amanda G. Henry, and Marco Candela. 2015. “Metagenome Sequencing of the Hadza Hunter-Gatherer Gut Microbiota.” Current Biology 25 (13): 1682–93. https://doi.org/10.1016/j.cub.2015.04.055.
- Sankaranarayanan, Krithivasan, Andrew T. Ozga, Christina Warinner, Raul Y. Tito, Alexandra J. Obregon-Tito, Jiawu Xu, Patrick M. Gaffney, et al. 2015. “Gut Microbiome Diversity among Cheyenne and Arapaho Individuals from Western Oklahoma.” Current Biology 25 (24): 3161–69. https://doi.org/10.1016/j.cub.2015.10.060.
- Schirmer, Melanie, Sanne P. Smeekens, Hera Vlamakis, Martin Jaeger, Marije Oosting, Eric A. Franzosa, Rob ter Horst, et al. 2016. “Linking the Human Gut Microbiome to Inflammatory Cytokine Production Capacity.” Cell 167 (4): 1125-1136.e8. https://doi.org/10.1016/j.cell.2016.10.020.
- Smits, Samuel A., Jeff Leach, Erica D. Sonnenburg, Carlos G. Gonzalez, Joshua S. Lichtman, Gregor Reid, Rob Knight, et al. 2017. “Seasonal Cycling in the Gut Microbiome of the Hadza Hunter-Gatherers of Tanzania.” Science 357 (6353): 802–6. https://doi.org/10.1126/science.aan4834.
- Vatanen, Tommi, Aleksandar D. Kostic, Eva d’Hennezel, Heli Siljander, Eric A. Franzosa, Moran Yassour, Raivo Kolde, et al. 2016. “Variation in Microbiome LPS Immunogenicity Contributes to Autoimmunity in Humans.” Cell 165 (4): 842–53. https://doi.org/10.1016/j.cell.2016.04.007.
- Vincent, Caroline, Mark A. Miller, Thaddeus J. Edens, Sudeep Mehrotra, Ken Dewar, and Amee R. Manges. 2016. “Bloom and Bust: Intestinal Microbiota Dynamics in Response to Hospital Exposures and Clostridium Difficile Colonization or Infection.” Microbiome 4 (1): 12. https://doi.org/10.1186/s40168-016-0156-3.
- Vogtmann, Emily, Xing Hua, Georg Zeller, Shinichi Sunagawa, Anita Y. Voigt, Rajna Hercog, James J. Goedert, Jianxin Shi, Peer Bork, and Rashmi Sinha. 2016. “Colorectal Cancer and the Human Gut Microbiome: Reproducibility with Whole-Genome Shotgun Sequencing.” PLOS ONE 11 (5): e0155362. https://doi.org/10.1371/journal.pone.0155362.
- Vrieze, Anne, Els Van Nood, Frits Holleman, Jarkko Salojärvi, Ruud S. Kootte, Joep F. W. M. Bartelsman, Geesje M. Dallinga–Thie, et al. 2012. “Transfer of Intestinal Microbiota From Lean Donors Increases Insulin Sensitivity in Individuals With Metabolic Syndrome.” Gastroenterology 143 (4): 913-916.e7. https://doi.org/10.1053/j.gastro.2012.06.031.
- Wen, Chengping, Zhijun Zheng, Tiejuan Shao, Lin Liu, Zhijun Xie, Emmanuelle Le Chatelier, Zhixing He, et al. 2017. “Quantitative Metagenomics Reveals Unique Gut Microbiome Biomarkers in Ankylosing Spondylitis.” Genome Biology 18 (1): 142. https://doi.org/10.1186/s13059-017-1271-6.
- Wu, Hao, Eduardo Esteve, Valentina Tremaroli, Muhammad Tanweer Khan, Robert Caesar, Louise Mannerås-Holm, Marcus Ståhlman, et al. 2017. “Metformin Alters the Gut Microbiome of Individuals with Treatment-Naive Type 2 Diabetes, Contributing to the Therapeutic Effects of the Drug.” Nature Medicine 23 (7): 850–58. https://doi.org/10.1038/nm.4345.
- Xie, Hailiang, Ruijin Guo, Huanzi Zhong, Qiang Feng, Zhou Lan, Bingcai Qin, Kirsten J. Ward, et al. 2016. “Shotgun Metagenomics of 250 Adult Twins Reveals Genetic and Environmental Impacts on the Gut Microbiome.” Cell Systems 3 (6): 572-584.e3. https://doi.org/10.1016/j.cels.2016.10.004.
- Ye, Zi, Ni Zhang, Chunyan Wu, Xinyuan Zhang, Qingfeng Wang, Xinyue Huang, Liping Du, et al. 2018. “A Metagenomic Study of the Gut Microbiome in Behcet’s Disease.” Microbiome 6 (1): 135. https://doi.org/10.1186/s40168-018-0520-6.
- Youngster, Ilan, Jenny Sauk, Christina Pindar, Robin G. Wilson, Jess L. Kaplan, Mark B. Smith, Eric J. Alm, Dirk Gevers, George H. Russell, and Elizabeth L. Hohmann. 2014. “Fecal Microbiota Transplant for Relapsing Clostridium Difficile Infection Using a Frozen Inoculum From Unrelated Donors: A Randomized, Open-Label, Controlled Pilot Study.” Clinical Infectious Diseases 58 (11): 1515–22. https://doi.org/10.1093/cid/ciu135.
- Yu, Jun, Qiang Feng, Sunny Hei Wong, Dongya Zhang, Qiao yi Liang, Youwen Qin, Longqing Tang, et al. 2017. “Metagenomic Analysis of Faecal Microbiome as a Tool towards Targeted Non-Invasive Biomarkers for Colorectal Cancer.” Gut 66 (1): 70–78. https://doi.org/10.1136/gutjnl-2015-309800.
- Zeevi, David, Tal Korem, Niv Zmora, David Israeli, Daphna Rothschild, Adina Weinberger, Orly Ben-Yacov, et al. 2015. “Personalized Nutrition by Prediction of Glycemic Responses.” Cell 163 (5): 1079–94. https://doi.org/10.1016/j.cell.2015.11.001.

- Zeller, Georg, Julien Tap, Anita Y Voigt, Shinichi Sunagawa, Jens Roat Kultima, Paul I Costea, Aurélien Amiot, et al. 2014. “Potential of Fecal Microbiota for Early-Stage Detection of Colorectal Cancer.” Molecular Systems Biology 10 (11): 766. https://doi.org/10.15252/msb.20145645.
- Zhang, Xuan, Dongya Zhang, Huijue Jia, Qiang Feng, Donghui Wang, Di Liang, Xiangni Wu, et al. 2015. “The Oral and Gut Microbiomes Are Perturbed in Rheumatoid Arthritis and Partly Normalized after Treatment.” Nature Medicine 21 (8): 895–905. https://doi.org/10.1038/nm.3914.
