# Supplementary material for: A catalogue of 1,167 genomes from the human gut archaeome
Source: Nat Microbiol. 2021 Dec 30;7(1):48–61. doi: 10.1038/s41564-021-01020-9 (PMC8727293; doi:10.1038/s41564-021-01020-9)
Supplement: Supplementary file 3 — Peer Review Information [file 41564_2021_1020_MOESM3_ESM.pdf]

natureresearch

Peer Review Information

Journal: Nature Microbiology
Manuscript Title: A catalogue of 1,167 genomes from the human gut archaeome
Corresponding author name(s): Christine Moissl-Eichinger

Reviewer Comments & Decisions:

Decision Letter, initial version:

Dear Christine,

Thank you for your patience while your manuscript "A comprehensive analysis of the global human gut archaeome from a thousand genome catalogue" was under peer-review at Nature Microbiology. It has now been seen by 3 referees, whose expertise and comments you will find at the end of this email. Although they find your work of some potential interest, they have raised a number of concerns that will need to be addressed before we can consider publication of the work in Nature Microbiology.

In particular, referees #1 and #2 suggest looking at relative abundances of these MAGs in human metagenomes using read mapping, and referee #2 suggests using a read mapper profiler tool, such as Kraken, to look at the composition of the archaeal gut microbiome. Referee #3 has concerns as to whether the thresholds for analyses (e.g. for the MAGs, gene catalog, CheckV etc.) are stringent enough, and asks for better comparison to metadata, which referee #2 also suggests e.g. links between archaea and disease. The figures also need improvement as concerns were raised by several referees.

Should further experimental data allow you to address these criticisms, we would be happy to look at a revised manuscript.

We are committed to providing a fair and constructive peer-review process. Please do not hesitate to contact us if there are specific requests from the reviewers that you believe are technically impossible or unlikely to yield a meaningful outcome.

We strongly support public availability of data. Please place the data used in your paper into a public data repository, if one exists, or alternatively, present the data as Source Data or Supplementary Information. If data can only be shared on request, please explain why in your Data Availability Statement, and also in the correspondence with your editor. For some data types, deposition in a public repository is mandatory - more information on our data deposition policies and available repositories can

be found at https://www.nature.com/nature-research/editorial-policies/reporting-standards#availability-of-data.

Please include a data availability statement as a separate section after Methods but before references, under the heading "Data Availability". This section should inform readers about the availability of the data used to support the conclusions of your study. This information includes accession codes to public repositories (data banks for protein, DNA or RNA sequences, microarray, proteomics data etc…), references to source data published alongside the paper, unique identifiers such as URLs to data repository entries, or data set DOIs, and any other statement about data availability. At a minimum, you should include the following statement: "The data that support the findings of this study are available from the corresponding author upon request", mentioning any restrictions on availability. If DOIs are provided, we also strongly encourage including these in the Reference list (authors, title, publisher (repository name), identifier, year). For more guidance on how to write this section please see: http://www.nature.com/authors/policies/data/data-availability-statements-data-citations.pdf

If revising your manuscript:

* Include a "Response to referees" document detailing, point-by-point, how you addressed each referee comment. If no action was taken to address a point, you must provide a compelling argument. This response will be sent back to the referees along with the revised manuscript.

* If you have not done so already we suggest that you begin to revise your manuscript so that it conforms to our Article format instructions at http://www.nature.com/nmicrobiol/info/final-submission. Refer also to any guidelines provided in this letter.

* Include a revised version of any required reporting checklist. It will be available to referees (and, potentially, statisticians) to aid in their evaluation if the manuscript goes back for peer review. A revised checklist is essential for re-review of the paper.

When submitting the revised version of your manuscript, please pay close attention to our href="https://www.nature.com/nature-research/editorial-policies/image-integrity">Digital Image Integrity Guidelines. and to the following points below:

-- that unprocessed scans are clearly labelled and match the gels and western blots presented in figures.
-- that control panels for gels and western blots are appropriately described as loading on sample processing controls
-- all images in the paper are checked for duplication of panels and for splicing of gel lanes.

Finally, please ensure that you retain unprocessed data and metadata files after publication, ideally archiving data in perpetuity, as these may be requested during the peer review and production process or after publication if any issues arise.

Please use the link below to submit a revised paper:

{redacted}

Note: This url links to your confidential homepage and associated information about manuscripts you may have submitted or be reviewing for us. If you wish to forward this e-mail to co-authors, please delete this link to your homepage first.

Nature Microbiology is committed to improving transparency in authorship. As part of our efforts in this direction, we are now requesting that all authors identified as 'corresponding author' on published papers create and link their Open Researcher and Contributor Identifier (ORCID) with their account on the Manuscript Tracking System (MTS), prior to acceptance. This applies to primary research papers only. ORCID helps the scientific community achieve unambiguous attribution of all scholarly contributions. You can create and link your ORCID from the home page of the MTS by clicking on 'Modify my Springer Nature account'. For more information please visit please visit www.springernature.com/orcid.

If you wish to submit a suitably revised manuscript we would hope to receive it within 6 months. If you cannot send it within this time, please let us know. We will be happy to consider your revision, even if a similar study has been accepted for publication at Nature Microbiology or published elsewhere (up to a maximum of 6 months).

In the meantime we hope that you find our referees' comments helpful.

With best wishes,

{redacted}

****
Reviewer Expertise:

Referee #1: archaea, genomics, ecology and evolution
Referee #2: archaea, metagenomics, bioinformatics
Referee #3: human gut microbiome, metagenomics, bioinformatics

Reviewer Comments:

Reviewer #1 (Remarks to the Author):

Review report NMICROBIOL-21051134-T "A comprehensive analysis of the global human gut archaeome from a thousand genome catalogue"

The paper by Moissl-Eichinger and co-workers focuses on human gut 'archaeome', describing over 1000 archaeal genomes recovered from a wide variety of human GI tract microbiomes – including several new genera and species. Moissl-Eichinger et al infer a number of correlations between the presence of archaeal protein clusters and host age and lifestyles. Furthermore, the paper reports that these are active constituents of GI tract communities, and display specific adaptations to their hosts. Finally, a diverse set of pro-viral sequences was identified that affiliate with the archaeal gut microbiome.

Major comments
While the manuscript describes an enormous wealth of data and results, and will certainly be informative for the human gut research community, it remains very descriptive. As an outsider from the human metagenome field, and having seen a multitude of papers presenting similar large scale analyses (just not on archaea), I wonder how relevant/significant the presented results are for the community. Of the >1100 archaeal MAGs, the vast majority (85%) belongs to a single genus, and only very few seem to really constitute 'new' components of the human gut microbiome. Surely, the current paper adds a lot of detail and resolution to the overlooked human gut archaeome, but does this lead to new insights? That the Methanobrevibacter smithii clade represents two separate species? That the human gut archaeome is actively replicating? The results on the archaeal gut virome analysis is novel and potentially interesting, but again very descriptive since there is not a lot of data to compare it to.

What I miss for example is information of the relative abundances of these archaeal MAGs in human subjects based on e.g. read mapping. Such analyses would provide useful insights into the potential relevance of these in the archaea in the human GI tract.

Generally, the (supplementary) figures are very information dense, and the content is not always clearly linked to the content of the manuscript. The small font size (and sometimes low resolution) makes the figure text hard to read.

The ‘discussion’ is very superficial, and is rather a perspective.

Other comments

Line 84: Define TMA here, not in line 93-94.

Line 99: Define MAG here, not in line 114.

Line 116 & Methods: The authors have used data from data repositories such as NCBI and IMG/M. Can the authors confirm that no data was used that was still under embargo by the primary generators of the data?

Line 123-133: The authors should elaborate on the choice of used the mentioned ANI cut-offs for species and strain delineation (or at least cite a reference for this). Without wanting to start a semantic discussion on what constitutes a species or strain, such ANI definitions are rather arbitrary.

Line 142: “Most… representatives”. Please be more specific, and refer to a figure or table that shows this.

Figure 1: Due to the small font, the text in the figure is hardly readable. Also, the Figure is extremely information dense, and much of the displayed information is seemingly not explicitly discussed in the main text. Should (parts of) this figure be converted to Suppl. Figure?

Line 158-161: To what does “genomes” (line 159) refer to? Are these the genomes or metagenomes of the subjects, the genomes of the archaea?

Line 161-163: This is confusing. Two-thirds of the subjects showed a diseased phenotype, but did also two-thirds of the subjects receive antibiotics? How is this linked (e.g. patients with colorectal cancer or diabetes should not need antibiotics)?

Line 163-164: This is an interesting statement; is this the result of a sampling bias? Can the authors give an explanation for this? Why are more archaeal genomes identified in ‘healthy female adults living in urban areas of Europe’? Large parts of Africa and South America (non-western regions) remain unsampled.

Line 166: Please provide context; proteins from where? And what is the definition of ‘lacking any functional annotation’? Please clarify.

Line 180-199: It is unclear how this rather technical section connects to the central focus of the paper (archaeal genomes in the human gut). This should be made more clear.

Line 167: Clustering was done based on protein sequences, not genes.

Figure 2: As for Figure 1, due to the small font, the text in the figure is hardly readable, and the figure is rather information dense. Should parts of this figure should be converted to Suppl. Figures.

Line 226: What does ‘singletons’ refer to here (genomes or proteins)? Singleton proteins could also be suspect of contaminants in MAGs.

Line 227-239: All these observations are based on the current dataset, which is subject to sampling bias (e.g. see 2A). As such, the statements made here about saturation of pangenomes are meaningless.

Line 292-300: The haloarchaeal MAG obtained seems very similar to the environmental strain from a lake. To what extent are the authors certain that this is an integrative constituent of the human gut archaeome?

Line 432-467: The observation that human-associated methanogens would experience less HGT compared to animals is interesting. It is however not properly explained in the methods (lines 809-814) what criteria have been used to determine which genes are the result of HGT. When is a gene considered ‘bacterial’ or ‘non-archaeal’? Are such HGT genes allowed to have homologs in other closely related archaea (pointing to more ‘ancient’ HGT events)? Such analyses could be easily affected by sampling biases of the selected genomes. Also this section is again very descriptive. Could the authors speculate on why human-associated archaea seem to experience lower HGT than animal-associated archaea?

Line 477: What do the authors mean with the term ‘diverge’? Please rephrase, this term is usually not used in this context.

Reviewer #2 (Remarks to the Author):

The study reports the analysis of 1,167 public archaeal genomes from human gastrointestinal tract samples covering five continents. The authors identify 15 novel species, 52 novel archaeal strains, a diverse set of archaeal proviruses, and propose splitting the most abundant archaeal species, Methanobrevibacter smithii, into two species. A total of 28,581 protein clusters derived from these genomes showed correlations with social-demographic characteristics of the human donors.

This is a very well executed analysis and a well-written manuscript. The study investigates a wide range of gut metagenomes from 24 countries and therefore contributes considerably to our understanding of the archaeal gut microbiome. The detection of archaeal taxa not previously reported for the human gastrointestinal tract is remarkable, although some of these taxa may only be transient residents as the authors acknowledge. The sections on archaeal proviruses, HGT, and metabolic inferences further expands the large scope and significance of this ms.

My only concern is that this ms compares prevalence based on genome presence/absence, using mostly MAGs, and does not attempt to analyse archaeal relative abundances in the human gastrointestinal tract samples. Reporting the number of MAGs recovered per sample, country, etc. is a worthwhile exercise, however, MAG recovery is usually dependent on the ability to assemble and bin genomes of certain taxa. This in turn depends mainly on sequencing depth and sample diversity, and in particular, on strain heterogeneity. Therefore, the success or failure of recovering a MAG should not be used to evaluate or discuss the presence/absence of a taxon in a given sample. Doing so can lead to unsupported conclusions.
For example, in line 298 the authors report that only two genomes affiliated with halophilic archaea are available for the human GIT and that this observation is in contrast to previous reports of high prevalence of Haloarchaea in Asian cohorts. This is not an equal comparison since it compares the ability of recovering MAGs, and obtaining cultures, against results from a 16S rRNA gene survey (Kim et al. 2020). Or in line 319, where the authors report that Methanobrevibacter oralis MAGs were not recovered from their dataset, although this species has been isolated from human stool previously (Khelaifia et al. 2014a). It could very well be that many M. oralis strains are present in the authors' dataset, but that this

strain heterogeneity impedes the binning algorithm, preventing MAG recovery.
A robust approach to analyse the prevalence and abundance of archaeal lineages such as halophiles and M. oralis will be to use one of the many published read-based community profiler tools (e.g. Kraken, Ganon, Bracken, MetaCache, etc.).

Determining the archaeal species composition this way will allow the authors to provide a more accurate picture of the gut archaeome and will make this manuscript a reference for years to come.
Using these results together with the rich metadata accompanying this dataset, the authors could explore the association of Archaea with disease, e.g. dysbiosis or infections that could be associated with higher prevalence and abundance of methanogenic archaea.

Other than that, I have only several minor comments:

L32: "…. showed substantial correlation"
What is the definition of a substantial correlation? Was the correlation significant or not?

L126: I assume this should say 99.9% average nucleotide identity (ANI)?

Fig. 1 A, B,C while these figures are beautifully crafted, the colours are impossible to differentiate. For example, in 1A it would be helpful to indicate which coloured bars represent which rank – I assume its order, genus, species? 1B and 1C might need additional labels or the colour scheme needs to be reduced, e.g. to show only higher ranks.
Also, I am not sure if the number of t-RNAs is such a useful measure, since most standard annotation pipelines are not very good in identifying t-RNAs in some archaeal groups. Showing the genome completeness instead, which is nearly impossible to decipher from 2C due to overlapping bubbles, might be more helpful to convey the quality of the genome dataset.

L182 "the protein catalogue was down-sampled to a minimum of 50 genomes for each tested metadata category" Please add a sentence to explain how this was done, how were the 50 genomes selected?

L183: The biological relevance of some of these findings, e.g. the high correlation for the lifestyle of an individual with genome completeness, seems rather questionable.

L235: While excluding singletons might be helpful to avoid false positives from contaminated MAGs, a data set with all singleton genes excluded likely underestimates the true diversity. I appreciate that the authors acknowledge this limitation. However, the opposing results obtained from the rarefaction analysis with and without singletons leave the reader wondering what to make of it.

286: The authors introduce a new genus and propose the name "Candidatus Methanoprimatia hominis" for representatives of Mx-06. However, this is a species name.

376: This reviewer has no experience with GRiD, but the GitHub page describes Growth Rate Index (GRiD) as a tool to measure bacterial growth rates from reference genomes. Can one assume that this tool is suitable for Archaea as well?

398 "91 viral species representatives were found to be specific for Methanobrevibacter ". But it seems Fig. 5 shows that most viruses were detected in Methanobrevibacter_A

Fig. 5. It would be helpful to add a sentence explaining what a checkV "high" or "medium" quality classification means.

Reviewer #3 (Remarks to the Author):

In this study, Chibani et al investigate the better-quality subset of 1,167 archaeal genome bin clusters drawn from a collection of existing human gut metagenome assemblies (plus isolates). Some of the authors involved in the work have recently been pursuing several investigations into other aspects of these large-scale metagenome assemblies, and this study represents a focus specifically on the subset that make up putative archaea. Major claims from the work include 1) sequences detected from a wide range of previously uncultured taxa, 2) associations of archaeal gene content with host covariates, particularly geography, 3) archaeally-associated viruses, and 4) confirmation at scale of previously reported Methanobrevibacter smithii subclades.

The manuscript continues a recent trend of exploring metagenomic assemblies and microbial comparative genomics from metagenomes, and its focus on archaea is appreciated and interesting. While the methodology is essentially a rinse-and-repeat of previous papers on bacteria, viruses, and other data resources, it has not yet been deeply applied to archaea. As a result, the basic framework of the analysis and its resulting data are phenomenal and have great potential. However, their presentation in the manuscript as written is frequently quite problematic, in all three of biological content, methods, and format (particularly figures). Suggestions for improving the study include:

Major comments:

* It is very surprising that from the Abstract on, the authors make explicit claims of new species discovery - and naming - solely from metagenomic assemblies (and not particularly highly-curated ones at that). In the absence of a cultured isolate, this is still not standard practice. References to “species-like clades” or defining a term to mean roughly “metagenomic assembly bins containing approximately the same genetic diversity commonly accepted as a species” would be more appropriate.

* No Results text is provided at all on the definition of “archaeal” used to select MAGs from the previous studies, or whether steps were required to harmonize their contents. The Methods themselves only state “We selected archaeal genomes...” This is so fundamental to the manuscript’s analysis that it requires at least a description, if not also further QC and quantitative analysis (since to my knowledge, none of the previous studies explicitly QCed the archaeal subset of their MAGs).

* The thresholds used for putative archaeal MAG inclusion are fairly lenient; 50% completeness and 5% contamination are quite generous when comparing MAGs to high-quality isolate genomes. Even among the previous studies included here, higher thresholds were used for most analyses (e.g. completeness >90% in 30661755). At the least, an investigation of the effects of QC on the final set of selected representatives should be included (since it’s possible these thresholds rarely matter if all ~1,100 selected centroids are themselves high-quality).

* Similarly - but to a likely greater degree - the clustering criteria for the protein catalog are fairly lenient for some of the purposes that they’re used for. 50% amino acid identity is fine for catalog construction and sample classification based on machine learning, but it is substantially below reliable functional homology (for which 80% is a more reasonable ballpark). This is thus unlikely to affect the covariate analysis, but it makes many of the functional claims beginning on line 531 (“Functional and metabolic interaction...”) difficult to substantiate (especially since the entire high-throughput annotation catalog is being transferred only via homology in the first place).

* Somewhat relatedly, the quality control statistics provided for the gene catalog are extremely suspect. This is perhaps most visible in Figure 3B, which implies that archaeal species have pan-genomes on the order of several hundred thousand protein families each. Since even Escherichia, known for having a very open pan-genome, has a pan-genome of at most a couple hundred thousand families (31068915), and 30661755 typically found bacterial pan-genomes on the order of tens of thousands, these numbers

are not especially plausible.

Some possible reasons for this, which would then also influence other aspects of the study:

-- The generous contamination rate of the MAGs (5%), which could by itself include ~a few hundred incorrect ORFs per genome.

-- Handling of fragmentary, short, or partial ORFs (see 33290720 for a thorough QC pipeline regarding these; alternatively, at scale, they can simply be omitted as a minority pan-genome component). These can *greatly* inflate diversity estimates in communities, since many short, similar proteins can achieve high coverage rates but still not be complete.

-- Handling of singletons, which is not clear from the current text. They were explicitly excluded from the 27 representative genomes (line 226), but apparently included for some (but not all) subsequent analyses (e.g. contrast line 236 with line 168). This point overall would benefit from revision for clarity/accuracy.

-- Failure to exclude low-level sequence or algorithmic noise during clustering. This is exactly the same problem that plagued early amplicon-based diversity estimates, e.g. contrast PMID 16848637 vs 27190214).

* The discussion of an archaeal "virome" (e.g. line 388) is extremely confusing, since no explicitly viral sequencing at all is included. Instead, the authors analyze proviruses as identified by CheckV (again, at fairly lenient quality thresholds), and the analysis consists mainly of finding that they are not substantively homologous to previously identified environmental archaeal phages. Of course, the latter would also arise from the inclusion of noise in either the assemblies or viral classifications. I would strongly suggest this section be rewritten to strengthen the QC of putative viral sequence, to present the data resource without additional speculation, and to focus on only validatable, positive results rather than those that would be difficult to differentiate from noise without further information.

* Similarly, the analysis of human-associated archaeal HGTs - and particularly its comparison to those of animal-associated archaea - is extremely speculative. Most notably, almost all animal-associated archaeal genomes analyzed are from isolates; almost all human-associated genomes are from MAGs. The generally-lower quality of the latter can easily lead to HGT-like sequences due to contamination or chimeric assemblies, and almost no description of the HGT analysis was provided in Methods (line 809).

* Relatedly, on lines 437-438, it is not clear how the animal-originating genomes were chosen for the analysis except for the information listed in Table S11. As the selection of animal strain genomes would directly impact the comparison results, details should be included on what criteria are those genomes being involved, and why those animal-originated genomes were of different species/clades (e.g. none of them were type A M. smithii). If the selected archaea are simply phylogenetically distinct from those in MAGs, this is more a comparison of HGT in one part of the archaeal phylogeny vs. another than human vs. non-human archaea (there's also a huge difference between "animal" defined as mammal vs. "animal" defined as tube worm, for example).

* The data sharing for such an important resource is somewhat thin. The authors point to a single tarball for all data, which contains only GFF files (zero documentation, as far as I can tell). There is no clear information on how to link these back to 1) the representative assemblies from which they were called, 2) their containing MAG bin clusters, 3) the originating metagenomes, 4) gene family clusters in which individual ORFs are contained, or 5) non-Prodigal annotations used during this study (e.g. putative viral loci). Particularly since the authors centralized many of these resources in a previous publication (32690973), this information should not be difficult to include so that readers can take better advantage of this study's data products.

* This would not normally be a major comment, but the figures provided with the manuscript are of consistently very low quality. There are substantially more than would be typical of a well-organized Nature-family paper, they do not always contain critical information, they are often illegibly or inconsistently labeled, and they are not well-linked to corresponding text or analyses. In addition to simply moving less-relevant information to the supplement and focusing main-text figures on the most important information, some suggestions include:

-- Figure 1. Almost none of the information presented here is actionable as shown. Genome labels in 1A are not meaningful and they are too small to be readable; the 99% ANI strain ring is redundant and can be removed; B and C are QC information that would fit better in supplementary figures; D-F could be visualized better (the alluvial plot does not help the reader visualize the distribution of data, see https://doi.org/10.1016/j.cell.2019.01.001 for effective presentation of categorical metadata). A legend for radius size is missing from B&C, and taxon level here has not been established.

-- Figure 2. Little of the geographic information in panels A-C is relevant (particularly as shown graphically in A), and these are redundant with 1D's summary. If geography is an important covariate, it should be shown phylogenetically as per previous publications. Legends, labels, and often panel contents are blurry throughout. 2B is clearly a plot rotated 90 degrees, without the axis title fixed or any information about the data size (y axis). D: Unclear why the dimensions of the heatmap don't reflect the size of clustered data: 3,050 proteins (columns) x 1,167 archaeal genomes (rows).

-- Figure 3. This is a very inefficient representation of the pan-genome diversity information, which could be better-summarized as simple Chao1, ACE, or other richness estimates. However, as described above, its contents are currently somewhat suspect regardless.

-- Figure 4. Again, labels and axes are too small, and genome IDs are not meaningful. We appreciate 4A but found the 4B-G were not referenced sufficiently in the text for the reader to understand the point to be taken from this figure. We suggest moving the M. smithii taxa/M. smithii protein ordination from Figure 2 to this figure, thus focusing on M. smithii results here. Some of 4B-G can be removed if they are not needed to directly support any conclusions from the text.

-- Figure 5 is confusing - and, as above, does not clearly contain accurate data. Labels of each viral cluster are too small to be meaningful. It is not clear what the 255 total number of objects referred to when none of the numbers mentioned in the manuscript was indicated in the figure (line 389-393: identified 94 viral populations by clustering 175 high or low quality archaeal proviruses, where one to a maximum of two proviruses were identified per host).

-- Figure 6 and Figure 7 are impossible to interpret without quantitative labeling. No information is given as to the percentages or colors referenced or the sizes of bubbles. To a large degree, all Figure 7 conveys is that "MAGs from the current study are different from previously assembled isolates," which is not surprising by any measure.

-- Figure 8A, same problem with the labels of genomes and the pathways. Genome labels are not informative here and can be removed. CAS systems are never even mentioned in the manuscript.

Moderate comments:

* Line 252 (Novel members of the human gastrointestinal archaeome, particularly in Asian and Oceanian populations): We appreciate this section but feel the organization and analysis could be strengthened. It's currently very anecdotal, comprising a laundry-list of disconnected statements made with no follow-up (e.g. M. cuniculi, Methanosphaera, halophilic archaea, fiji islands, Methanobacterium, Mx-06). Many of these could remain combined under the theme of MAGs/genomes not previously observed in the GIT as a

clear focus for this paper, and/or given their own richer analyses in a separate follow-up manuscript.

-- As described below for strains of Methanobrevibacter being included here, they might be cleaner to move this into the M. smithii section, which could broadly focus on Methanobrevibacter and M. smithii/A, being the most heavily represented species.

* Relatedly, in Figure 4 (and the text itself), the different clades of M. smithii are interesting, but have been reported previously. We feel this is a strong starting point for the paper, but not a sufficient conclusion. Similarly to the comments above, if it was to be a unique focus of either this or a separate paper, we would recommend further work to describe the genes/functions in each subclade and do more analysis on their potential functions (as per previous work, e.g. 31607556).

* While the text (particularly toward the beginning) makes strong claims regarding the association of archaeal taxa and gene families with host properties, only geography is analyzed in any depth.

Minor comments:

* Generally define acronyms throughout when introduced (e.g. line 99 MAG, line 127 ANI, line 216 PCoA, line 241 GIT, etc.)

Author Rebuttal to Initial comments

Reviewer Comments:

Reviewer #1 (Remarks to the Author):

Review report NMICROBIOL-21051134-T "A comprehensive analysis of the global human gut archaeome from a thousand genome catalogue"

The paper by Moissl-Eichinger and co-workers focuses on human gut 'archaeome', describing over 1000 archaeal genomes recovered from a wide variety of human GI tract microbiomes – including several new genera and species. Moissl-Eichinger et al infer a number of correlations between the presence of archaeal protein clusters and host age and lifestyles. Furthermore, the paper reports that these are active constituents of GI tract communities, and display specific adaptations to their hosts. Finally, a diverse set of pro-viral sequences was identified that affiliate with the archaeal gut microbiome.

Major comments

→ While the manuscript describes an enormous wealth of data and results, and will certainly be informative for the human gut research community, it remains very descriptive. As an outsider from the human metagenome field, and having seen a multitude of papers presenting similar large scale analyses (just not on archaea), I wonder how relevant/significant the presented results are for the community.

Authors reply: *We aimed to create an archaea reference catalogue and set a basis for the knowledge accumulated so far derived from sequence data. In recent large-scale efforts mentioned by reviewer 1 the information and analysis on archaea is scarce or missing completely - though the archaeome is an important component of the human microbiome. Thus our study is unique and novel. Besides, we refer to the statement of referee #2: "Determining the archaeal species composition this way will allow the authors to provide a more accurate picture of the gut archaeome and will make this manuscript a reference for years to come. " And referee #3: "its focus on archaea is appreciated andinteresting".*

→ Of the >1100 archaeal MAGs, the vast majority (85%) belongs to a single genus, and only very few seem to really constitute 'new' components of the human gut microbiome. Surely, the current paper adds a lot of detail and resolution to the overlooked human gut archaeome, but does this lead to new insights? That the Methanobrevibacter smithii clade represents two separate species? That the human gut archaeome is actively replicating? The results on the archaeal gut virome analysis is novel and potentially interesting, but again very descriptive since there is not a lot of data to compare it to.

Authors reply: *While we do agree many results remain descriptive at this point, and in some cases it is just not possible to compare our information to a lot of data as is possible for bacteria, we aimed to set a basis for future archaea research as more information becomes available.*

Additionally and most important for future microbiome studies, we aimed to present several new species (and proviruses) identified to be part of the human microbiome, which were not previously described or known, and to our knowledge, the assessment of the active replication of the human gut archaeome is indeed a novel approach.

→ What I miss for example is information of the relative abundances of these archaeal MAGs in

human subjects based on e.g. read mapping. Such analyses would provide useful insights into the potential relevance of these in the archaea in the human GI tract.

Authors reply: *We agree that this is definitely a valid point, and would indeed add great value to our research. Therefore we added additional analyses and the following text:*

Information added in Material and Methods in a new section:

Relative abundances of archaea in human metagenomes (lines 752 ff)

"Raw reads were obtained from studies of the human gut microbiome out of which 691 (691 of 1,167) medium- or high-quality archaeal MAGs were assembled. The rest was not made public by their original submitters (Table S1).
We mapped the raw metagenomic reads to the 27 reference archaeal species representatives using Bowtie2-align (Langmead and Salzberg 2012) and post-processed using samtools (Li et al. 2009). The sorted mapping files were used to calculate the depth of coverage (Table S15).
Reads were additionally mapped using Kraken v2.1.2 (Wood et al. 2019) (with default settings) against 1) a custom database of the UHGG catalog available from the MGnify FTP site (http://ftp.ebi.ac.uk/pub/databases/metagenomics/mgnify_genomes/human-gut/v1.0/uhgg_kraken2-db/) and 2) a custom database of the 27 archaeal species representatives in our dataset as we supplemented the initial resource with additional isolates (see Data description section). Results were processed using Bracken v2.5.3 (Lu et al. 2017) and a read length of 100 to estimate the relative abundance of domain, family and species-level taxa (Table S15)."

Results added:

Lines 291ff: "Although a high prevalence of haloarchaea was previously reported in Asian cohorts (Kim et al. 2020), none of the two genomes in our study reached a breadth coverage over 2% when performing read mappings of 691 datasets (Table S15). In addition, none of the two genomes revealed obvious adaptations towards the human gut ecosystem, such as bile salt hydrolases."

Lines 306ff: "Based on read recruitment, Methanobacterium sp000499765 is found in 1 (ERR479173) out of 691 studies with an average breadth of coverage of 99.76 % (Table S15)."

Lines 327ff: "However, based on read mapping, M.oralis is found in 68 out of 691 studies with an average breadth of coverage ranging between 30 and 70%, and ~100% in two studies (ERR479327 and ERR479417) (Table S15)."

Supplementary Table added:

Table S15. Prevalence and abundance of archaeal lineages in the human GIT. Read-based community profiler output based on UHGG catalog and the 27 archaeal species representatives of the human gut. The relative abundance of archaea was estimated at the domain, family and species-level taxa. Relative abundance of each species representative in all studies where raw data was available based on read mapping and breadth of coverage computation.

→ Generally, the (supplementary) figures are very information dense, and the content is not always clearly linked to the content of the manuscript. The small font size (and sometimes low

resolution) makes the figure text hard to read.

Authors reply: *We agree, the Figures have been revised based on reviewers comments. Concerning Supp Figures, we have made sure that every supp. Figure is referenced in the text and we added additional explanation in the supplementary Figure legend, wherever possible and necessary.*

→ The ‘discussion’ is very superficial, and is rather a perspective.
Authors reply: *We agree. The discussion was rewritten and re-organized, and additional information was included.*

Other comments

→ Line 84: Define TMA here, not in line 93-94.
Authors reply: *Corrected*.

→ Line 99: Define MAG here, not in line 114.
Authors reply: *Corrected*.

→ Line 116 & Methods: The authors have used data from data repositories such as NCBI and IMG/M. Can the authors confirm that no data was used that was still under embargo by the primary generators of the data?
Authors reply: *We confirm that no unpublished data were used. All Genomes collected from NCBI and IMG/M were already published. Details of each associated publication is summarized in Table S1 (sheet 4). **Lines 101 ff.***

→ Line 123-133: The authors should elaborate on the choice of used the mentioned ANI cut-offs for species and strain delineation (or at least cite a reference for this). Without wanting to start a semantic discussion on what constitutes a species or strain, such ANI definitions are rather arbitrary.
Authors reply: *The choice of ANI cut-offs including citation (Jain et al. 2018 for species delineation) are detailed in Materials and Methods under section Genome quality and taxonomic classification.*

L717 ff: “DRep v2.0.0 (Olm et al. 2017) was used to dereplicate the complete dataset at 95% and 99% ANI values. 95% ANI values were selected to separate between species boundaries (n=27) (Jain et al. 2018). A cutoff of 99% was selected for strain delineation provided a stable number of clusters for MAGs over 75% complete and had less than 5% contamination (n=98; Fig. S3). Lower thresholds did not affect the number of strains recovered.”

Line 142: “Most… representatives”. Please be more specific, and refer to a figure or table that shows this.
Authors reply: *We apologize for not being precise. We updated the statement for clarity. It now reads “A large proportion of genomes not matching any known species(n=83) and genera (n=10) are affiliated to the order Methanomassiliicoccales” (Line 128 f).*

→ Figure 1: Due to the small font, the text in the figure is hardly readable. Also, the Figure is extremely information dense, and much of the displayed information is seemingly not explicitly discussed in the main text. Should (parts of) this figure be converted to Suppl. Figure?
Authors reply: *We agree and modified the tree accordingly to increase text and to optimize readability.*

→ Line 158-161: To what does “genomes” (line 159) refer to? Are these the genomes or metagenomes of the subjects, the genomes of the archaea?
Authors reply: *“Genomes” refers to genome sequences from both isolates and MAGs. Please see:*

Line 108F: “Henceforth, unless otherwise stated, “genomes” refers to genome sequences from both isolates and MAGs.”

→ Line 161-163: This is confusing. Two-thirds of the subjects showed a diseased phenotype, but did also two-thirds of the subjects receive antibiotics? How is this linked (e.g. patients with colorectal cancer or diabetes should not need antibiotics)?
Authors reply: *We apologize for the confusion. These are separate observations. We updated the statement in the text and included exact numbers.*

Lines 158 ff: “The subset of the genomes from which metadata was available includes samples from rural and urban populations of 24 countries (five continents; Table S1; Fig. 2A) and includes more than 800 archaeal genomes from infants, children, teenagers, adults and elderly subjects spanning all Body Mass Index (BMI) groups (underweight up to extreme obesity class 3; details on available metadata are given in Methods). Almost three-hundred subjects (n=302) showed a diseased phenotype (e.g. colorectal cancer, type 2 diabetes, infection), out of which subjects receiving antibiotic treatment (n=19), had an infection (n=9) or were obese (n=2). Eight subjects were administered antibiotics nevertheless no data was available about disease. However, most genomes (3rd quartile, 75% of all values) were obtained from healthy female adults of normal weight, living in urban areas of Europe (Fig. 2).”

→ Line 163-164: This is an interesting statement; is this the result of a sampling bias? Can the authors give an explanation for this? Why are more archaeal genomes identified in ‘healthy female adults living in urban areas of Europe’? Large parts of Africa and South America (non-western regions) remain unsampled.
Authors reply: *Indeed we cannot rule out sampling bias, thus clear answers cannot be given at this stage of analyses. We rephrased respective locations in the manuscript and added a statement in the discussion:*

Lines 602ff: “Using this comprehensive reference, we were able to make initial associations between the diversity of gut-associated archaea with several demographic and geographic patterns. However, many geographic locations remain undersampled to date.”

→ Line 166: Please provide context; proteins from where? And what is the definition of ‘lacking any functional annotation’? Please clarify.
Authors reply: All the proteins are identified from the 1,167 genomes. By lacking any functional annotation we mean they are annotated as hypothetical proteins. We now updated this in the manuscript.
Lines 167ff: “In total, 1.8 million proteins were identified from the 1,167 genomes, 54% of which were annotated as hypothetical proteins. A protein catalogue of all 1,167 archaeal genomes was generated by clustering the genes predicted across all genomes and excluding singletons clusters, resulting in 28,581 cluster representatives (>50% amino acid identity and >80% coverage) (Fig. S4; Table S2). 3,050 proteins (thereof 58% hypothetical proteins) were found to be shared among more than 50 genomes in our dataset, mirroring the taxonomic distance of the two most abundant orders, Methanomassiliicoccales and Methanobacteriales (Fig. 3A).”

→ Line 180-199: It is unclear how this rather technical section connects to the central focus of the paper (archaeal genomes in the human gut). This should be made more clear.
Authors reply: *We believe these results highlight the strong association between the archaeal composition with geographic and demographic patterns. This opens the possibility to investigate*

candidate proteins/functions further in the future. Given we compiled a total of 1.8 million proteins in our archaea catalog, we now have a smaller set of candidate features to prioritize in future research. Nevertheless, the entire paragraph was rephrased (Line 181 ff).

→ Line 167: Clustering was done based on protein sequences, not genes.
Authors reply: *Corrected*.

→ Figure 2: As for Figure 1, due to the small font, the text in the figure is hardly readable, and the figure is rather information dense. Should parts of this figure should be converted to Suppl. Figures.
Authors reply: *Figure 2 has been updated, redundant information was excluded.*

→ Line 226: What does 'singletons' refer to here (genomes or proteins)? Singleton proteins could also be suspect of contaminants in MAGs.
Authors reply: *We apologize for not being precise - we mean proteins, we now corrected in the manuscript accordingly:*

Line 168 ff: "A protein catalogue of all 1,167 archaeal genomes was generated by clustering the genes predicted across all genomes and excluding singletons clusters, resulting in 28,581 cluster representatives (>50% amino acid identity and >80% coverage) (Fig. S4; Table S2)."

→ Line 227-239: All these observations are based on the current dataset, which is subject to sampling bias (e.g. see 2A). As such, the statements made here about saturation of pangenomes are meaningless.
Authors reply: *We agree with the reviewer and revised this section, redid the analysis with Panaroo as it is better in handling annotation errors, fragment assemblies and contaminated genomes. We updated this section according to the new results (see reply to reviewer 2 and 3 for details).*

→ Line 292-300: The haloarchaeal MAG obtained seems very similar to the environmental strain from a lake. To what extent are the authors certain that this is an integrative constituent of the human gut archaeome?
Authors reply: We extended the information as follows:

Lines 291ff: "Although a high prevalence of haloarchaea was previously reported in Asian cohorts (Kim et al. 2020), none of the two genomes in our study reached a breadth coverage over 2% when performing read mappings of 691 datasets (Table S15). In addition, none of the two genomes revealed obvious adaptations towards the human gut ecosystem, such as bile salt hydrolases. We thus conclude that haloarchaea might be transient residents of the human GIT."

→ Line 432-467: The observation that human-associated methanogens would experience less HGT compared to animals is interesting. It is however not properly explained in the methods (lines 809-814) what criteria have been used to determine which genes are the result of HGT. When is a gene considered 'bacterial' or 'non-archaeal'? Are such HGT genes allowed to have homologs in other closely related archaea (pointing to more 'ancient' HGT events)? Such analyses could be easily affected by sampling biases of the selected genomes. Also this section is again very descriptive. Could the authors speculate on why human-associated archaea seem to experience lower HGT than animal-associated archaea?
Authors reply: *A more detailed explanation is now added in the Methods section:*

Lines 837ff: “Initial horizontal gene transfer analysis
Representative genomes from isolates and MAGs with 0% contamination according to CheckM results were selected for these analyses (*Methanosphaera*: 8 human, 7 animal;

Methanobrevibacter: 30 from human, 11 from animal). A list with full details is provided in Table S11. Genomes from animals were obtained from NCBI (ncbi.nlm.nih.go/genome), representing all available high-quality genomes (isolates, MAGs) of the respective genus at the time point of analysis (2020; Table S11). The selected genomes were further characterized as previously mentioned using eggNOG mapper v2.0.0 (Huerta-Cepas et al. 2017) and the previously mentioned databases (see section Genome annotation and protein catalogue). Annotated genes were sorted according to their taxonomic affiliation (eggNOG output information: "best_tax_level"), and the proportion of archaeal and bacterial genes was calculated for all genomes and genera. Data were visualized using Krona (Ondov, Bergman, and Phillippy 2011)."

We did not analyze the homologs yet, but this will be an important focus for upcoming analyses. We agree on the sampling bias. This was partially ruled out by concentrating on genomes from isolates AND MAGs with 0% contamination. We assume that human-associated archaea seem to experience lower HGT due to a more stringent selection process, driven by e.g. urbanization and thus developing a specialized microbiome, but at this point it is too early to include this speculation in the manuscript.

→ Line 477: What do the authors mean with the term 'diverge'? Please rephrase, this term is usually not used in this context.
Authors reply: *We mean distantly related, now corrected in the revised manuscript (Line 468).*

Reviewer #2 (Remarks to the Author):

The study reports the analysis of 1,167 public archaeal genomes from human gastrointestinal tract samples covering five continents. The authors identify 15 novel species, 52 novel archaeal strains, a diverse set of archaeal proviruses, and propose splitting the most abundant archaeal species, Methanobrevibacter smithii, into two species. A total of 28,581 protein clusters derived from these genomes showed correlations with social-demographic characteristics of the human donors.

This is a very well executed analysis and a well-written manuscript. The study investigates a wide range of gut metagenomes from 24 countries and therefore contributes considerably to our understanding of the archaeal gut microbiome. The detection of archaeal taxa not previously reported for the human gastrointestinal tract is remarkable, although some of these taxa may only be transient residents as the authors acknowledge. The sections on archaeal proviruses, HGT, and metabolic inferences further expands the large scope and significance of this ms.

→ My only concern is that this ms compares prevalence based on genome presence/absence, using mostly MAGs, and does not attempt to analyse archaeal relative abundances in the human gastrointestinal tract samples.

Authors reply: *That is definitely a valid concern, and was also raised by reviewer 1. To address this concern we added a section on "Relative abundances of archaea MAGs in human metagenomes" in the manuscript (**Lines 754 ff**). Please refer to the Author's reply to reviewer 1 on the matter and the section in the manuscript for details.*

→ Reporting the number of MAGs recovered per sample, country, etc. is a worthwhile exercise, however, MAG recovery is usually dependent on the ability to assemble and bin genomes of certain taxa. This in turn depends mainly on sequencing depth and sample diversity, and in particular, on strain heterogeneity. Therefore, the success or failure of recovering a MAG should not be used to evaluate or discuss the presence/absence of a taxon in a given sample. Doing so can lead to unsupported conclusions.

For example, in line 298 the authors report that only two genomes affiliated with halophilic archaea are available for the human GIT and that this observation is in contrast to previous reports of high prevalence of Haloarchaea in Asian cohorts. This is not an equal comparison since it compares the ability of recovering MAGs, and obtaining cultures, against results from a 16S rRNA gene survey (Kim et al. 2020). Or in line 319, where the authors report that Methanobrevibacter oralis MAGs were not recovered from their dataset, although this species has been isolated from human stool previously (Khelaifia et al. 2014a).

Authors reply: *We agree with the reviewer in that MAG recovery is not necessarily a measure of species presence/absence. Therefore, as mentioned above, we have added additional information on the abundance and coverage calculated through metagenomic read mapping. Regarding M. oralis, based on read mapping, is found in 68 out of 691 studies with an average breadth of coverage ranging between 30 and 70%, and ~100% in two studies (ERR479327 and ERR479417) (Table S15). So since we recover metagenomic reads mapping to the M. oralis genome in our catalog, we assume it likely represents a potential resident species:*

Lines 327: "Although this species is a normal member of the human oral microbiome, it has been isolated at least once from human stool (Khelaifia et al. 2014a). However, based on read mapping, M. oralis was detected in 68 out of 691 study datasets with an average breadth of coverage ranging between 30 and 70%, and reaching even ~100% in two studies (Table S15). Thus, a potential niche of M. oralis also in the human GIT cannot be excluded."

*See also Lines **291 ff** for the haloarchaea, including:* "In addition, none of the two genomes revealed obvious adaptations towards the human gut ecosystem, such as bile salt hydrolases. We thus conclude that haloarchaea might be transient residents of the human GIT."

→ It could very well be that many M. oralis strains are present in the authors' dataset, but that this strain heterogeneity impedes the binning algorithm, preventing MAG recovery.
Authors reply: *See our response to your previous comment.*

→ A robust approach to analyse the prevalence and abundance of archaeal lineages such as halophiles and M. oralis will be to use one of the many published read-based community profiler tools (e.g. Kraken, Ganon, Bracken, MetaCache, etc.).
Authors reply: *We agree and now implemented Kraken2 and Bracken and run against the human gut database (http://ftp.ebi.ac.uk/pub/databases/metagenomics/mgnify_genomes/human-gut/v1.0/uhgg_kraken2-db/). The result adds great value to our research. Therefore we added the text below in the manuscript:*

Information added in Material and Methods in a new section:

Lines 754ff: Relative abundances of archaea in human metagenomes

Raw reads were obtained from studies of the human gut microbiome out of which 691 (691 of 1,167) medium- or high-quality archaeal MAGs were assembled. The rest was not made public by their original submitters (Table S1). We mapped raw reads to the 27 reference archaeal species representatives using Bowtie2-align (Langmead and Salzberg 2012) and post-processed using samtools (Li et al. 2009). The generated sorted mapping files are used to calculate the breadth of coverage (Table S15). Reads were additionally mapped using Kraken v2.1.2 (Wood et al. 2019) (with default settings) against 1) a custom database of the UHGG catalog available from the MGnify FTP site (http://ftp.ebi.ac.uk/pub/databases/metagenomics/mgnify_genomes/human-gut/v1.0/uhgg_kraken2-db/) and 2) a custom database of the 27 archaeal species representatives in our dataset as we supplemented the initial resource with additional isolates (see Data description section). Results were processed using Bracken v2.5.3 (Lu et al. 2017) and a read length of 100 and 250, which did not lead to differences, to estimate the relative abundance of domain, family and species-level taxa (Table S15).

Information added in Results section:

Lines 242ff: The dataset reveals novel members of the human gastrointestinal archaeome particularly in Asian and Oceanian populations
For species Halorubrum lipolyticum and Haloferax massiliensis: **Line 292**
For species Methanobacterium sp000499765: **Line 306**
And for species Methanobrevibacter oralis: **Line 328**

In Discussion section (Lines 619ff):

"The overall observed percentage of archaea present in the human gut microbiomes (approx. 1.1 %, Supplementary Table S15) is in agreement with a recently reported average percentage based on 16S rRNA marker gene and shotgun metagenomic information"

Supplementary Table added:

Table S15. Prevalence and abundance of archaeal lineages in the human GIT. Read-based community profiler output based on UHGG catalog and the 27 archaeal species representatives of the human gut. The relative abundance of archaea was estimated at the domain, family and

species-level taxa. Relative abundance of each species representative in all studies where raw data was available based on read mapping and breadth of coverage computation.

→ Determining the archaeal species composition this way will allow the authors to provide a more accurate picture of the gut archaeome and will make this manuscript a reference for years to come.
Using these results together with the rich metadata accompanying this dataset, the authors could explore the association of Archaea with disease, e.g. dysbiosis or infections that could be associated with higher prevalence and abundance of methanogenic archaea.
Authors reply: *We revealed significant associations of archaeal proteins with a healthy phenotype with MaAsLin2 and identified most predictive proteins for the lifestyle of an individual (**Lines 197ff**):*
"According to MaAsLin2 analysis, numerous hypothetical proteins showed significant associations with various metadata categories. For a few proteins with functional annotation, significant associations of e.g. adenylosuccinate synthetase, NADPH dehydrogenase, vitamin B12 import ATP-binding protein BtuD, Na(+)-translocating NADH- quinone reductase subunit F, or L-rhamnose 1-dehydrogenase (NADP(+)) could be observed for a healthy phenotype ((Table S7)."

As most features were only hypothetical proteins, we did not elaborate on these details in this manuscript. However, we tested all available metadata categories even in linear mixed effect models to screen for significant associations and summarized the main findings in the manuscript. We recognize that the provided datasets could be analyzed and mined in further detail, but we believe this would be beyond the scope of the current manuscript.

Other than that, I have only several minor comments:

→ L32: "…. showed substantial correlation"
What is the definition of a substantial correlation? Was the correlation significant or not?
Authors reply: *We used the term substantial since many proteins showed significant associations with diverse metadata categories. As we agree that the term "substantial" is misleading we revised this part and now talk about significant associations. (**Line 32**)*

→ L126: I assume this should say 99.9% average nucleotide identity (ANI)?
Authors reply: *Corrected*.

→ Fig. 1 A, B,C while these figures are beautifully crafted, the colours are impossible to differentiate. For example, in 1A it would be helpful to indicate which coloured bars represent which rank – I assume its order, genus, species? 1B and 1C might need additional labels or the colour scheme needs to be reduced, e.g. to show only higher ranks.
Also, I am not sure if the number of t-RNAs is such a useful measure, since most standard annotation pipelines are not very good in identifying t-RNAs in some archaeal groups. Showing the genome completeness instead, which is nearly impossible to decipher from 2C due to overlapping bubbles, might be more helpful to convey the quality of the genome dataset.

Authors reply: *All Figures were substantially re-worked based on Reviewers' comments.*

It shall be mentioned that we sticked to a color code already used previously in Borrel et al., 2020. For consistency, we do not change the palettes per genus or family however we sharpened the colors for a better visualization and differentiation.

We have now included contour plots in supplementary Fig. 2 to improve the display. Supplementary Fig. 2 also includes the information of tRNAs vs MAG completeness.

→ L182 "the protein catalogue was down-sampled to a minimum of 50 genomes for each tested metadata category" Please add a sentence to explain how this was done, how were the 50 genomes selected?
Authors reply: *Genomes were not selected. All metadata categories which were represented by less than 50 genomes were simply not included in the analysis.*

→ L183: The biological relevance of some of these findings, e.g. the high correlation for the lifestyle of an individual with genome completeness, seems rather questionable.
Authors reply*: These were separate observations. We agree: We now removed the information on genome completeness correlations from the manuscript.*

→ L235: While excluding singletons might be helpful to avoid false positives from contaminated MAGs, a data set with all singleton genes excluded likely underestimates the true diversity. I appreciate that the authors acknowledge this limitation. However, the opposing results obtained from the rarefaction analysis with and without singletons leave the reader wondering what to make of it.
Authors reply: *We agree with the reviewer and revised this section* ***(Lines 221 ff),*** *redid the analysis with Panaroo as it is better in handling annotation errors, fragment assemblies and contaminated genomes. We now modified and updated this section based on the new results. We now state that the genera Methanobrevibacter (995 genomes, α=0.72), and of the Methanomassiliicoccus (27 genomes, α=0.96), have an open pangenome. While for the genera Methanomethylophilaceae UBA71 clade (66 genomes, α=1.1), Methanosphaera (29 genomes, α=1.31), and Methanomethylophilus (38 genomes, α=1.54) have a closed pangenome.*

Members of the Haloferax, Halorubrum, Methanobacterium, Methanocorpusculum, Methanomethylophilaceae ISO4-G1 and Methanomethylophilaceae unassigned genera do not have enough members and therefore remain undersampled.

Members of the Haloferacaceae and Methanocorpusculaceae families have not enough members and therefore remain undersampled.

→ 286: The authors introduce a new genus and propose the name "Candidatus Methanoprimatia hominis" for representatives of Mx-06. However, this is a species name.
Authors reply: *Corrected*.

→ 376: This reviewer has no experience with GRiD, but the GitHub page describes Growth Rate Index (GRiD) as a tool to measure bacterial growth rates from reference genomes. Can one assume that this tool is suitable for Archaea as well?
Authors reply: *For the following reasons we are convinced that GRiD and also other tools based on peak to trough (PTR) ratios like iRep or DEMIC are suitable to predict growth rates from archaeal reference genomes as well. We assume that Archaea were simply neglected from the validation assays of these tools and were thus not explicitly mentioned:*

The PTR method was designed "to work as an in situ measure of the actual growth rate of any prokaryote in any sample where genomic or metagenomic data are available" and "this method is based on the observation that prokaryotes generally have circular genomes that are replicated bidirectionally from a fixed origin to a fixed terminus" (https://doi.org/10.1038/s41396- 020-00773-1). Hence, more rapidly growing and replicating populations are expected "to have more reads recruited to and near the origin of replication ("peak") compared to the terminus

("trough")". We consider this trait as a rather universal phenomenon in the prokaryotic world that should be valid for Archaea as well.

We could also detect essential features for archaeal replication like Orc1/Cdc6, MCM and PCNA (PMID: 17158702) in our methanogenic archaea. This suggests a common archaeal architecture for the replication machinery of our MAGs.

Nevertheless we are aware that the original PTR method was designed for closed and complete genomes (doi: 10.1126/science.aac4812). Therefore we used methods like iRep (doi:10.1038/nbt.3704) and GRiD (https://doi.org/10.1038/s41467-018-07240-8) that are capable to infer replication or growth rates even from fragmented incomplete MAGs. Due to its better performance for genomes with an extremely low coverage and a mean mappable read fraction of 0.012% we based our interpretations on the output from GRiD (https://doi.org/10.1038/s41467-018-07240-8).

Still, we want to give cause for concern that growth rate index estimates might be biased towards fast growing organisms and do not reflect actual growth rates in the habitat as benchmarked in a recent publication by Long and co-authors (https://doi.org/10.1038/s41396- 020-00773-1). Since direct comparisons might be misleading we based our analysis only on relative proportions.

398 "91 viral species representatives were found to be specific for Methanobrevibacter ". But it seems Fig. 5 shows that most viruses were detected in Methanobrevibacter_A
Authors reply: *We apologize for this mistake. We have corrected the figure in the new version of the manuscript.*

→ Fig. 5. It would be helpful to add a sentence explaining what a checkV "high" or "medium" quality classification means.
Authors reply: The explanation of the high and medium quality classification is mentioned in the Materials and Methods section **Line 872**, as well as in **Line 386** of the Results. We have now also added the explanation to the legend of Fig. 5 for additional clarity.

Reviewer #3 (Remarks to the Author):

In this study, Chibani et al investigate the better-quality subset of 1,167 archaeal genome bin clusters drawn from a collection of existing human gut metagenome assemblies (plus isolates). Some of the authors involved in the work have recently been pursuing several investigations into other aspects of these large-scale metagenome assemblies, and this study represents a focus specifically on the subset that make up putative archaea. Major claims from the work include 1) sequences detected from a wide range of previously uncultured taxa, 2) associations of archaeal gene content with host covariates, particularly geography, 3) archaeally-associated viruses, and 4) confirmation at scale of previously reported Methanobrevibacter smithii subclades.

→ The manuscript continues a recent trend of exploring metagenomic assemblies and microbial comparative genomics from metagenomes, and its focus on archaea is appreciated and interesting. While the methodology is essentially a rinse-and-repeat of previous papers on bacteria, viruses, and other data resources, it has not yet been deeply applied to archaea. As a result, the basic framework of the analysis and its resulting data are phenomenal and have great potential. However, their presentation in the manuscript as written is frequently quite problematic, in all three of biological content, methods, and format (particularly figures). Suggestions for improving the study include:
Authors reply: *We thank the reviewer evaluating ' resulting data are phenomenal and have great potential. And essentially yes, we did follow previous manuscripts' methodologies while focusing on archaea. We addressed all the below mentioned comments for biological content, methods and we improved all figures.*

It is worth mentioning that we now added a section on ***Relative abundances of archaea in human metagenomes*** *based on read recruitment and profiling and read coverage depth (See reply to reviewer 1).*

Major comments:

* It is very surprising thatfrom the Abstract on, the authors make explicit claims of new species discovery - and naming - solely from metagenomic assemblies (and not particularly highly- curated ones at that). In the absence of a cultured isolate, this is still not standardpractice.
References to "species-like clades" or defining a term to mean roughly "metagenomic assembly bins containing approximately the same genetic diversity commonly accepted as a species" would be more appropriate.
Authors reply: *We appreciate the reviewer's hesitation to name new microbial species without cultivated isolates, as this has been the prevailing standard in microbiology. However, with the advent of massive-scale metagenomics, the community is realizing the need to label and classify uncultivated species to provide a stable nomenclature to potentially important new taxa. Therefore, we have followed recent standards and community proposals of how to potentially name uncultivated archaea, and suggest a naming scheme for abundant uncultivated archaea according to the references indicated below:*

- *Murray, A.E., Freudenstein, J., Gribaldo, S., Hatzenpichler, R., Hugenholtz, P., Kämpfer, P., Konstantinidis, K.T., Lane, C.E., Papke, R.T., Parks, D.H. and Rossello-Mora, R., 2020. Roadmap*

for naming uncultivated Archaea and Bacteria. Nature microbiology, 5(8), pp.987-994.

- *Pallen, M.J., Telatin, A. and Oren, A., 2020. The Next Million Names for Archaea and Bacteria. Trends in Microbiology.*

Please note, that for the proposed new Candidatus taxonomic names, isolates, or additional biological information is already available, e.g. Candidatus Methanobrevibacter intestini, who is represented by isolate WWM1085.

* No Results text is provided at all on the definition of "archaeal" used to select MAGs from the previous studies, or whether steps were required to harmonize their contents. The Methods themselves only state "We selected archaeal genomes..." This is so fundamental to the manuscript's analysis that it requires at least a description, if not also further QC and quantitative analysis (since to my knowledge, none of the previous studies explicitly QCed the archaeal subset of their MAGs).
Authors reply: *We agree this is fundamental, and we extended the statement that archaea were defined and selected using the GTDB.*

Lines 725: "The taxonomic assignment was performed using the Genome Taxonomy Database Toolkit (GTDB-Tk) version 0.3.1 (database release 04-RS89) (Chaumeil et al. 2020) and default parameters which utilizes a set of 122 marker genes to identify archaeal MAGs. Novelty of species and genera was defined when no taxonomic information was assigned for all members of a species cluster and their species representatives based on the GTDB database."

* The thresholds used for putative archaeal MAG inclusion are fairly lenient; 50% completeness and 5% contamination are quite generous when comparing MAGs to high-quality isolate genomes. Even among the previous studies included here, higher thresholds were used for most analyses (e.g. completeness >90% in 30661755). At the least, an investigation of the effects of QC on the final set of selected representatives should be included (since it's possible these thresholds rarely matter if all ~1,100 selected centroids are themselves high-quality).

Authors reply: *We appreciate the reviewer's concern regarding the quality of MAGs. We should clarify that even though our catalog includes MAGs of at least 50% completeness, we did not make any inferences or conclusions based on MAGs that were <75% complete. In fact, . genome dereplication with dRep at both species and strain level was performed using thresholds of 75% completeness. In addition, we even obtained 75 strains and 730 species that were >90% complete.*

Furthermore, in the revised manuscript we now additionally used a more recent tool (GUNC) to further control for potential chimerism in the MAGs. Importantly, this new method did not flag any of our genomes as being potentially chimeric.

We stress the cutoff for the strain and species list in the results in section:

Over 1,000 unique archaeal genomes recovered from human gastrointestinal samples

Lines 112ff: "This procedure yielded 1,167 non-chimeric (clade separation score (CSS) = 0; Orakov et al. 2021; Table S1) and non-redundant archaeal genomes (Mash distance threshold of 0.001, 99.9% average nucleotide identity (ANI); Ondov et al. 2016; Table S1) which were further sub-grouped into individual strains (<99% ANI similarity, >75% genome completeness; Table S1 and Fig. S3; 98 genomes) (Fig. 1), and species (<95% ANI similarity, >75% genome completeness; Table S1; 27 genomes)."

* Similarly - but to a likely greater degree - the clustering criteria for the protein catalog are fairly lenient for some of the purposes that they’re used for. 50% amino acid identity is fine for catalog construction and sample classification based on machine learning, but it is substantially below reliable functional homology (for which 80% is a more reasonable ballpark). This is thus unlikely to affect the covariate analysis, but it makes many of the functional claims beginning on line 531

("Functional and metabolic interaction...") difficult to substantiate (especially since the entire high-throughput annotation catalog is being transferred only via homology in the first place). **Authors reply:** *Due to the fact that we include different families, we clustered the proteins at different percent identities (100, 99, 98, 97, 96, 95, 90, 85, 80 and 50%) which was visualized in Figure S4. We observed a drop at 99% (strain boundary), 95% (species boundary), and at 50%. Therefore, to be able to cluster the proteins of the different families of such a diverse and distantly related dataset, we used our 50% identity threshold.*

The analysis based on the 50% clustered protein catalogue include only the heatmap and PCoA of Figure 2 and the predictive potential for some metadata categories.

Pangenomic using Roary before revision was based on 80% identity and 80% per genus, however during the revision process we used Panaroo in strict mode (see below) which according to Panaroo "To first build the graph, Panaroo runs CD-HIT (v4.8.1) at a high sequence identity threshold (98%)" and "In the 'strict' mode, Panaroo takes a more aggressive approach to contamination and erroneous annotation removal. This is most useful when investigating genomes where rare plasmids are not expected or when phylogenetic parameters such as gene gain and loss rates are of interest. In these cases, erroneous gene clusters can quickly dominate the estimated parameters."

We did not make conclusions or assumptions based on proteins found once, especially in the section of ***Lines 181 ff:*** *The protein catalogue had also predictive potential for some metadata categories...*

*For the protein catalogue we state (**Lines 173 ff**):*

"To overcome biases introduced by potential residual MAGs contamination issues, we focused our analyses on patterns observed in two or more genomes, unless stated otherwise."

* Somewhat relatedly, the quality control statistics provided for the gene catalog are extremely suspect. This is perhaps most visible in Figure 3B, which implies that archaeal species have pan-genomes on the order of several hundred thousand protein families each. Since even Escherichia, known for having a very open pan-genome, has a pan-genome of at most a couple hundred thousand families (31068915), and 30661755 typically found bacterial pan-genomes on the order of tens of thousands, these numbers are not especiallyplausible.
Authors reply: *We should clarify that for Methanobrevibacter_A family, the pangenome size is of 50,000 clusters and not 500,000. Nevertheless, to further improve the quality of the analysis, we have re-generated pan-genomes using the tool Panaroo in strict mode to account for potential contaminations (see reply to reviewer 2 on what has changed or refer to section "The genetic diversity of the gut archaeome still remains undersampled" in the manuscript).*

Some possible reasons for this, which would then also influence other aspects of the study:

-- The generous contamination rate of the MAGs (5%), which could by itself include ~a few hundred incorrect ORFs per genome.
Authors reply: *We ran GUNC, a robust new method for the detection of chimerism and contamination in prokaryotic genomes. All of the genomes had an estimated clade separation score (CSS) of 0. We added*

the method in materials and methods, the output as an additional sheet in Table S1, and a statement about chimerism in section "Over 1,000 unique archaeal genomes recovered from human gastrointestinal samples" ***Line 112****:*

"This procedure yielded 1,167 non-chimeric (clade separation score (CSS) = 0; Orakov et al. 2021; Table S1) and non-redundant archaeal genomes (Mash distance threshold of 0.001,

99.9% average nucleotide identity (ANI); Ondov et al. 2016; Table S1) which were further sub-grouped into individual strains (<99% ANI similarity, >75% genome completeness; Table S1 and Fig. S3; 98 genomes) (Fig. 1), and species (<95% ANI similarity, >75% genome completeness; Table S1; 27 genomes)."

-- Handling of fragmentary, short, or partial ORFs (see 33290720 for a thorough QC pipeline regarding these; alternatively, at scale, they can simply be omitted as a minority pan-genome component). These can *greatly* inflate diversity estimates in communities, since many short, similar proteins can achieve high coverage rates but still not be complete.
Authors reply: The proteins were all annotated with Prokka, which only predicts full-length genes. Prokka calls the ORF-caller prodigal with the "-c" argument (for "closed ends"), which won't let ORFs run over the contig ends (See **Lines 732 ff**).

-- Handling of singletons, which is not clear from the current text. They were explicitly excluded from the 27 representative genomes (line 226), but apparently included for some (but not all) subsequent analyses (e.g. contrast line 236 with line 168). This point overall would benefit from revision for clarity/accuracy.
Authors reply: *We thank the reviewer for pointing this out and accordingly revised the section "The genetic diversity of the gut archaeome still remains undersampled" (mentioned Lines* ***222 ff****): We redid the analysis with the tool Panaroo as it is better in handling annotation errors, fragment assemblies and contaminated genomes. We updated this section based on the new results and excluded Figure 3. We ran micropan to get the alpha values of each matrix output of panaroo and simply wrote in the text the alpha value per genus and family and stated whether the pangenome is open or close (see reply to reviewer 2 on what has changed or refer to section "The genetic diversity of the gut archaeome still remains undersampled" in the manuscript).*

For the mentioned ***L168*** *"A protein catalogue of all 1,167 archaeal genomes was generated by clustering the genes predicted across all genomes and excluding singletons, resulting in 28,581 cluster representatives", we clustered all proteins using MMseqs2 and removed the singletons clusters not counting them as part of the ~29K.*

After reviewers' comments, we only exclude singletons from the protein catalogue in the revised version of the manuscript.

-- Failure to exclude low-level sequence or algorithmic noise during clustering. This is exactly the same problem that plagued early amplicon-based diversity estimates, e.g. contrast PMID 16848637 vs 27190214).
Authors reply: *Having now run the pan-genome analysis with Panaroo in strict mode, we were able to better control for the presence of contamination and inconsistencies in protein annotation. Panaroo uses a graph-based pan-genome clustering approach to account for many possible sources of errors (see further details in the associated publication: https://genomebiology.biomedcentral.com/articles/10.1186/s13059-020-02090-4).*

* The discussion of an archaeal "virome" (e.g. line 388) is extremely confusing, since no explicitly viral sequencing at all is included. Instead, the authors analyze proviruses as identified by CheckV (again, at fairly lenient quality thresholds), and the analysis consists mainly of finding that they are not

substantively homologous to previously identified environmental archaeal phages. Of course, the latter would also arise from the inclusion of noise in either the assemblies or viral classifications. I would strongly suggest this section be rewritten to

strengthen the QC of putative viral sequence, to present the data resource without additional speculation, and to focus on only validatable, positive results rather than those that would be difficult to differentiate from noise without further information.
Authors reply: *We stressed in the manuscript that we exclusively used proviruses flanked by archaeal genomic regions, as well as those classified by CheckV as medium, high quality and complete (see materials and methods). We now additionally added that we predicted the viral sequences first via VirSorter2 then assigned quality estimates via CheckV added the table in Supp. Table 10.*

Originally we hoped to compare to known archaeal viruses infecting related genera/families as we have no similarities in the GVD. However this sentence is now excluded.

* Similarly, the analysis of human-associated archaeal HGTs - and particularly its comparison to those of animal-associated archaea - is extremely speculative. Most notably, almost all animal- associated archaeal genomes analyzed are from isolates; almost all human-associated genomes are from MAGs. The generally-lower quality of the latter can easily lead to HGT-like sequences due to contamination or chimeric assemblies, and almost no description of the HGT analysis was provided in Methods (line 809).
Authors reply: *A more detailed description of the methods used is now added.*

Lines 838ff: "Initial horizontal gene transfer analysis
Representative genomes from isolates and MAGs with 0% contamination according to CheckM results were selected for these analyses (*Methanosphaera*: 8 human, 7 animal; *Methanobrevibacter*: 30 from human, 11 from animal). A list with full details is provided in Table S11. Genomes from animals were obtained from NCBI (ncbi.nlm.nih.go/genome), representing all available high-quality genomes (isolates, MAGs) of the respective genus at the time point of analysis (2020; Table S11). The selected genomes were further characterized as previously mentioned using eggNOG mapper v2.0.0 (Huerta-Cepas et al. 2017) and the previously mentioned databases (see section Genome annotation and protein catalogue). Annotated genes were sorted according to their taxonomic affiliation (eggNOG output information: "best_tax_level"), and the proportion of archaeal and bacterial genes was calculated for all genomes and genera. Data were visualized using Krona (Ondov, Bergman, and Phillippy 2011)."
Please note that we exclusively concentrated on isolates and MAGs with high quality (0% contamination).

Lines 423f: "For this comparison, and to rule out false information from contaminating reads, we used only genomes from isolates and MAGs with 0% contamination according to the CheckM results."

Additional information is available in the Figure legend of Fig. 6 (Lines 459ff):

"Only MAGs with 0% contamination and of high quality (taken from "strain list") and genomes from isolates were analyzed (full details are provided in Table S11) using eggNOG mapper v2.0.0 (Huerta-Cepas et al. 2017). Annotated genes were sorted according to their taxonomic affiliation (eggNOG output information: "best_tax_level"), and the proportion of archaeal and bacterial genes was calculated."

Besides now the wording is toned down further, in order to emphasize that the data are of course preliminary.

As mentioned above, we have also additionally checked that none of our genomes are chimeric using GUNC.

* Relatedly, on lines 437-438, it is not clear how the animal-originating genomes were chosen for the analysis except for the information listed in Table S11. As the selection of animal strain genomes would directly impact the comparison results, details should be included on what criteria are those genomes being involved, and why those animal-originated genomes were of different species/clades (e.g. none of them were type A M. smithii). If the selected archaea are simply phylogenetically distinct from those in MAGs, this is more a comparison of HGT in one part of the archaeal phylogeny vs. another than human vs. non-human archaea (there's also a huge difference between "animal" defined as mammal vs. "animal" defined as tube worm, for example).
Authors reply: *Additional details are now added:*

Lines 839ff: "Representative genomes from isolates and MAGs with 0% contamination according to CheckM results were selected for these analyses (Methanosphaera: 8 human, 7 animal; Methanobrevibacter: 30 from human, 11 from animal). A list with full details is provided in Table S11. Genomes from animals were obtained from NCBI (ncbi.nlm.nih.go/genome), representing all available high-quality genomes (isolates, MAGs) of the respective genus at the time point of analysis (2020; Table S11). The selected genomes were further characterized as previously mentioned using eggNOG mapper v2.0.0 (Huerta-Cepas et al. 2017) and the previously mentioned databases (see section Genome annotation and protein catalogue)."
We included all available high-quality genomes of Methanosphaera and Methanobrevibacter at the time point of analyses (source: NCBI). Methanobrevibacter smithii is solely associated with humans, thus representative genomes from animals are naturally missing. Unfortunately, genome sequences from other animals as those presented were not available, and could not be included. We are aware of the preliminary information on this topic and further toned down our language to emphasize the nature of information.

* The data sharing for such an important resource is somewhat thin. The authors point to a single tarball for all data, which contains only GFF files (zero documentation, as far as I can tell). There is no clear information on how to link these back to 1) the representative assemblies from which they were called, 2) their containing MAG bin clusters, 3) the originating metagenomes, 4) gene family clusters in which individual ORFs are contained, or 5) non-Prodigal annotations used during this study (e.g. putative viral loci). Particularly since the authors centralized many of these resources in a previous publication (32690973), this information should not be difficult to include so that readers can take better advantage of this study's data products.
Authors reply: *Species representatives and pan-genome details from a previous publication (32690973) are hosted on the MGnify database under the MGnify accession numbers (Table S1). The tarball is for the download of all genomes, not just the species representatives. A sentence is added in the text to clarify under "Resource Availability" section. 1, 2 and 3) provided in Table S1 ,4) provided in the protein clustering matrix which was the basis for the gene catalogue heatmap of Fig. 2 D, 5) provided in table S10*

* This would not normally be a major comment, but the figures provided with the manuscript are of consistently very low quality. There are substantially more than would be typical of a well- organized Nature-family paper, they do not always contain critical information, they are often illegibly or inconsistently labeled, and they are not well-linked to corresponding text or analyses. In addition to simply moving less-relevant information to the supplement and focusing main-text figures on the most important information, some suggestions include:

Authors reply: *In general, all figures have been updated according to reviewers' comments (see also Reviewer 1 and 2)*

-- Figure 1. Almost none of the information presented here is actionable as shown. Genome labels in 1A are not meaningful and they are too small to be readable; the 99% ANI strain ring is redundant and can be removed; B and C are QC information that would fit better in supplementary figures; D-F could be visualized better (the alluvial plot does not help the reader visualize the distribution of data, seehttps://doi.org/10.1016/j.cell.2019.01.001 for effective presentation of categorical metadata). A legend for radius size is missing from B&C, and taxon level here has not been established.
Authors reply: *Tree was modified according to the reviewer comments. Figure was divided into two figures now. The scatter plots (former Fig. 1B and Fig. 1C were revised and are now shown as contour plots in the Supplementary (Supplementary Fig. 2) as we could not observe a distinct pattern for specific taxonomic groups and our main message, that our lion's share of the dataset are high quality MAGs is easier visible in contour plots.*

We consider alluvial plots as a well accepted and appealing method to represent different dimensions of categorical (not numerical, where we prefer violin or rain cloud plots) data. Nevertheless, we revised the alluvial plot to improve readability. We focused on three separate plots showing only three levels each. We aligned all plots at the top and sorted all nodes in descending order. This should help the reader to understand important proportions of the main metadata categories. In addition, we used more distinct colors for our age groups.

-- Figure 2. Little of the geographic information in panels A-C is relevant (particularly as shown graphically in A), and these are redundant with 1D's summary. If geography is an important covariate, it should be shown phylogenetically as per previous publications. Legends, labels, and often panel contents are blurry throughout. 2B is clearly a plot rotated 90 degrees, without the axis title fixed or any information about the data size (y axis). D: Unclear why the dimensions of the heatmap don't reflect the size of clustered data: 3,050 proteins (columns) x 1,167 archaeal genomes (rows).
Authors reply: *Due to the redundant information, we exclude Fig 2 A,B and C. We proceeded to keep what was originally Fig2 D,E and F. For comment on Fig2 D, It is the other way around. 3,050 proteins (rows) x 1,167 archaeal genomes (columns). We corrected the sentence in the legend accordingly.*

-- Figure 3. This is a very inefficient representation of the pan-genome diversity information, which could be better-summarized as simple Chao1, ACE, or other richness estimates. However, as described above, its contents are currently somewhat suspect regardless.
Authors reply: *We excluded Fig.3 and simply added the alpha values in the text.*

-- Figure 4. Again, labels and axes are too small, and genome IDs are not meaningful. We appreciate 4A but found the 4B-G were not referenced sufficiently in the text for the reader to understand the point to be taken from this figure. We suggest moving the M. smithii taxa/M. smithii protein ordination from Figure 2 to this figure, thus focusing on M. smithii results here. Some of 4B-G can be removed if they are not needed to directly support any conclusions from the text.
Authors reply: *Fig. 4 we re-organized, reduced and labels were increased. The labels of the tree were reduced but kept, as it includes critical information on genome origin (DSMZ, MAG). The forest plot was moved to the Supplementary Information.*

-- Figure 5 is confusing - and, as above, does not clearly contain accurate data. Labels of each viral cluster are too small to be meaningful. It is not clear what the 255 total number of objects

referred to when none of the numbers mentioned in the manuscript was indicated in the figure (line 389-393: identified 94 viral populations by clustering 175 high or low quality archaeal proviruses, where one to a maximum of two proviruses were identified per host).

Authors reply: *Figure 5 has been updated according to the reviewer comments. The number 255 was a mistake, as it originally included proviruses and viruses. Since we decided to exclude viruses as they potentially could be a source of error and we just proceeded with proviruses which were flanked by archaea genes, the number of detected proviruses was 175. Labels have been excluded as they do not add additional meaningful information.*

-- Figure 6 and Figure 7 are impossible to interpret without quantitative labeling. No information is given as to the percentages or colors referenced or the sizes of bubbles. To a large degree, all Figure 7 conveys is that “MAGs from the current study are different from previously assembled isolates,” which is not surprising by any measure.
Authors reply: *Fig. 6: quantitative information is now more explicitely given in the figure legend. Fig. 7: quantitative information was added.*

For Figure 7, In order to proceed with zooming on selected genes in Fig.8 and specialized adaptations to the gut environment, we wanted to show that they are distantly related to relatives in the environment.

-- Figure 8A, same problem with the labels of genomes and the pathways. Genome labels are not informative here and can be removed. CAS systems are never even mentioned in the manuscript.
Authors reply: *The figures have been completely reworked. Fig. 8A is now part of Fig. 1. CAS systems have been removed.*

Moderate comments:

* Line 252 (Novel members of the human gastrointestinal archaeome, particularly in Asian and Oceanian populations): We appreciate this section but feel the organization and analysis could be strengthened. It’s currently very anecdotal, comprising a laundry-list of disconnected statements made with no follow-up (e.g. M. cuniculi, Methanosphaera, halophilic archaea, fiji islands, Methanobacterium, Mx-06). Many of these could remain combined under the theme of MAGs/genomes not previously observed in the GIT as a clear focus for this paper, and/or given their own richer analyses in a separate follow-up manuscript.
Authors reply: *We are aware that the nature of this publication is largely descriptive. However, we aim to show the broad wealth of information now available, as this publication should be the first of a series of follow-up papers, thus serving as an important basis for later-derived information. We hope the reviewer can agree that we did not completely re-write this chapter.*

We assume the information given in this chapter is highly-informative for the archaea scientific community.

-- As described below for strains of Methanobrevibacter being included here, they might be cleaner to move this into the M. smithii section, which could broadly focus on Methanobrevibacter and M. smithii/A, being the most heavily represented species.
Authors reply:*The Methanobrevibacter information was moved as suggested.*

* Relatedly, in Figure 4 (and the text itself), the different clades of M. smithii are interesting, but have been reported previously. We feel this is a strong starting point for the paper, but not a sufficient conclusion. Similarly to the comments above, if it was to be a unique focus of either this or a separate

paper, we would recommend further work to describe the genes/functions in each subclade and do more analysis on their potential functions (as per previous work, e.g. 31607556).

Authors reply: *We cite the latest Paper from Rinke et al. 2021 (see below) to reply to the comment that it has been reported previously. However we aim to present initial findings in this manuscript and we do plan to expand in future research.*

Rinke, C., Chuvochina, M., Mussig, A.J., Chaumeil, P.A., Davín, A.A., Waite, D.W., Whitman, W.B., Parks, D.H. and Hugenholtz, P., 2021. A standardized archaeal taxonomy for the Genome Taxonomy Database. Nature Microbiology, pp.1-14.

* While the text (particularly toward the beginning) makes strong claims regarding the association of archaeal taxa and gene families with host properties, only geography is analyzed in any depth.

Authors reply: *We do not think that we only analyzed geographic properties in sufficient detail. We included all available metadata categories in our linear mixed effect models, MaAsLin2 and supervised machine learning analysis. We summarized all results with a focus on significant associations, which might have concluded in the assessment that our analysis was solely focused on geographic properties.*

Minor comments:

* Generally define acronyms throughout when introduced (e.g. line 99 MAG, line 127 ANI, line 216 PCoA, line 241 GIT, etc.)

Authors reply: *Corrected.*

We thank all reviewers for the tremendous work and in-depth, and thoughtful comments!

Decision Letter, first revision:

Dear Christine,

Thank you for submitting your revised manuscript "A comprehensive analysis of the global human gut archaeome from a thousand genome catalogue" (NMICROBIOL-21051134A). It has now been seen by the original referees and their comments are below. Unfortunately referee #3 was unresponsive and so we asked referee #2 to comment on your responses to #3's original concerns. They felt that the majority of concerns had been addressed, apart from the need to include the relative abundance data in the results section.

The reviewers find that the paper has improved in revision, and therefore we'll be happy in principle to publish it in Nature Microbiology, pending minor revisions to satisfy the referees' final requests and to comply with our editorial and formatting guidelines.

If the current version of your manuscript is in a PDF format, please email us a copy of the file in an

editable format (Microsoft Word or LaTex)-- we can not proceed with PDFs at this stage.

We are now performing detailed checks on your paper and will send you a checklist detailing our editorial and formatting requirements in about a week. Please do not upload the final materials and make any revisions until you receive this additional information from us.

Thank you again for your interest in Nature Microbiology Please do not hesitate to contact me if you have any questions.

Sincerely,

{redacted}

Reviewer #1 (Remarks to the Author):

The revised manuscript by Moissl-Eichinger and co-workers represent a monumental effort in describing a wealth of information regarding the human gut archaeome; Clearly this work is important for the microbiome research community, not in the last place since archaea often end up neglected or omitted in microbiome studies. The authors have generally made a serious effort to address the comments raised by all three reviewers. The (content and readability of the) figures has improved, and several analyses and results that were unclear or for which details were lacking, have now been improved. Some issues however still could be addressed to further improve the clarity and quality of the manuscript prior to publication:

- The manuscript remains very much descriptive in nature, and I feel that the authors could have done a better job in presenting the results against the background of available knowledge/literature. I agree that literature on archaea in the human microbiome is limited, but some contrasts or parallels with more bacteria-focused studies could surely be made (e.g. regarding diversity, abundance, geographic patterns, virome, etc).

- The authors have made an effort to also incorporate information about abundances of archaea in the GI-tract, but the results of these additional analyses are hardly mentioned/described in the main text (tucked away in Table S15 and methods). A general overview of the distribution and abundance of archaeal lineages would be of interest to present in a figure and discussed in the main text. Which archaea are general constituents, and which more transiently associated (mentioning the potential limitations pointed out by ref 2).

- Line 371-383 and line 816-822: Some archaea (including methanogenic archaea, see here: https://environmentalmicrobiome.biomedcentral.com/articles/10.1186/s40793-016-0183-5) have been shown/are inferred to have multiple origins of replication, and are hence not suitable for analyses with tools such as iRep and GRiD to determine growth rates. The authors should acknowledge this, and tone down (or better, omit) any inferences to estimate growth rates.

Other than this I am happy to see this work published soon; it surely represents an important baseline repository for future human microbiome studies.

Reviewer #2 (Remarks to the Author):

The authors responded to all comments and mention in their rebuttal that they now also provide relative abundance estimates of domain, family and species-level taxa. However, these relative abundance data do not seem to be provided in Table S15 (see below). Furthermore, the authors refer to abundance data in the introduction "… we gain new insights into the abundance, distribution, composition and function of the human archaeome" but do not seem to include the abundances in the results section of the manuscript. Instead, they report the average breadth of coverage values, which are a good addition to the manuscript, but not a measure of relative abundance.

My further detailed comments are:

"Raw reads were obtained from studies of the human gut microbiome out of which 691 (691 of 1,167) medium- or high-quality archaeal MAGs were assembled. The rest was not made public by their original submitters (Table S1)."
Please add the number of the raw read datasets (651?) used for read mapping.

"We mapped raw reads to the 27 reference archaeal species representatives using Bowtie2-align (Langmead and Salzberg 2012) and post-processed using samtools (Li et al. 2009). The generated sorted mapping files are used to calculate the breadth of coverage (Table S15)."
The reader will appreciate more details here, e.g. how was the breadth of reference genome coverage calculated?

"Results were processed using Bracken v2.5.3 (Lu et al. 2017) and a read length of 100 and 250, which did not lead to differences, to estimate the relative abundance of domain, family and species-level taxa (Table S15)."
This sentence seems incomplete.

"Table S15. Prevalence and abundance of archaeal lineages in the human GIT. Read-based community profiler output based on UHGG catalog and the 27 archaeal species representatives of the human gut. The relative abundance of archaea was estimated at the domain, family and species-level taxa."
Table S15 does not seem to provide relative abundances of domain, family and species-level taxa, but rather shows only the fraction of overall reads mapping to 15 species, titled "27SpeciesResp", and a second tab lists the genome coverage percentages across all data sets.

Fig. 1
When proposing novel taxa it is important to consider the stability (e.g. bootstrap value) of the node decorated with the taxon label. The authors should provide this information in the tree, or at least state that all internal nodes decorated with new taxa have a minimum bootstrap support >90%, or similar.
Some entries in the "Genus" category are actually families, e.g. Methanomethylophilaceae - did the authors intend the show the genus Methanomethylophilus?
I am still not too excited about the way the taxa are visualised in this figure since it means going back and forth between the figure and the legend trying to decipher rather similar colours. However, if the authors opt to keep this many colours, they should at least show the taxa in the figure and the legend in the same order, e.g. start the order legend with Methanobacteriales. I also recommend checking if the colour scheme is colour-blind proof.
Furthermore, I assume the "Genome length" is the genome size which has been calculated as the average in cases where multiple genomes were recovered per species? Providing a standard deviation would be helpful. Also, the genome size of MAGs should be considered in regard to the estimated completeness.

naturereresearch

Decision Letter, final checks:

Dear Christine,

Thank you for your patience as we've prepared the guidelines for final submission of your Nature Microbiology manuscript, "A comprehensive analysis of the global human gut archaeome from a thousand genome catalogue" (NMICROBIOL-21051134A). Please carefully follow the step-by-step instructions provided in the attached file, and add a response in each row of the table to indicate the changes that you have made. Please also check and comment on any additional marked-up edits we have proposed within the text. Ensuring that each point is addressed will help to ensure that your revised manuscript can be swiftly handed over to our production team.

We would like to start working on your revised paper, with all of the requested files and forms, as soon as possible (preferably within two weeks). Please get in contact with us if you anticipate delays.

When you upload your final materials, please include a point-by-point response to any remaining reviewer comments.

If you have not done so already, please alert us to any related manuscripts from your group that are under consideration or in press at other journals, or are being written up for submission to other journals (see: https://www.nature.com/nature-research/editorial-policies/plagiarism#policy-on-duplicate-publication for details).

In recognition of the time and expertise our reviewers provide to Nature Microbiology's editorial process, we would like to formally acknowledge their contribution to the external peer review of your manuscript entitled "A comprehensive analysis of the global human gut archaeome from a thousand genome catalogue". For those reviewers who give their assent, we will be publishing their names alongside the published article.

Nature Microbiology offers a Transparent Peer Review option for new original research manuscripts submitted after December 1st, 2019. As part of this initiative, we encourage our authors to support increased transparency into the peer review process by agreeing to have the reviewer comments, author rebuttal letters, and editorial decision letters published as a Supplementary item. When you submit your final files please clearly state in your cover letter whether or not you would like to participate in this initiative. Please note that failure to state your preference will result in delays in accepting your manuscript for publication.

Cover suggestions

As you prepare your final files we encourage you to consider whether you have any images or illustrations that may be appropriate for use on the cover of Nature Microbiology.

Covers should be both aesthetically appealing and scientifically relevant, and should be supplied at the best quality available. Due to the prominence of these images, we do not generally select images featuring faces, children, text, graphs, schematic drawings, or collages on our covers.

We accept TIFF, JPEG, PNG or PSD file formats (a layered PSD file would be ideal), and the image

should be at least 300ppi resolution (preferably 600-1200 ppi), in CMYK colour mode.

If your image is selected, we may also use it on the journal website as a banner image, and may need to make artistic alterations to fit our journal style.

Please submit your suggestions, clearly labeled, along with your final files. We’ll be in touch if more information is needed.

Nature Microbiology has now transitioned to a unified Rights Collection system which will allow our Author Services team to quickly and easily collect the rights and permissions required to publish your work. Approximately 10 days after your paper is formally accepted, you will receive an email in providing you with a link to complete the grant of rights. If your paper is eligible for Open Access, our Author Services team will also be in touch regarding any additional information that may be required to arrange payment for your article.

Please note that you will not receive your proofs until the publishing agreement has been received through our system.

Please note that <i>Nature Microbiology</i> is a Transformative Journal (TJ). Authors may publish their research with us through the traditional subscription access route or make their paper immediately open access through payment of an article-processing charge (APC). Authors will not be required to make a final decision about access to their article until it has been accepted. Find out more about Transformative Journals

<B>Authors may need to take specific actions to achieve compliance with funder and institutional open access mandates. For submissions from January 2021, if your research is supported by a funder that requires immediate open access (e.g. according to Plan S principles) then you should select the gold OA route, and we will direct you to the compliant route where possible. For authors selecting the subscription publication route our standard licensing terms will need to be accepted, including our self-archiving policies. Those standard licensing terms will supersede any other terms that the author or any third party may assert apply to any version of the manuscript.

For information regarding our different publishing models please see our Transformative Journals page. If you have any questions about costs, Open Access requirements, or our legal forms, please contact ASJournals@springernature.com.

Please use the following link for uploading these materials:
{redacted}
If you have any further questions, please feel free to contact me.

With best wishes,
{ redacted}

Reviewer #1:
Remarks to the Author:
The revised manuscript by Moissl-Eichinger and co-workers represent a monumental effort in describing a wealth of information regarding the human gut archaeome; Clearly this work is important for the microbiome research community, not in the last place since archaea often end up neglected or omitted in microbiome studies. The authors have generally made a serious effort to address the comments raised by all three reviewers. The (content and readability of the) figures has improved, and several analyses and results that were unclear or for which details were lacking, have now been improved. Some issues however still could be addressed to further improve the clarity and quality of the manuscript prior to publication:

- The manuscript remains very much descriptive in nature, and I feel that the authors could have done a better job in presenting the results against the background of available knowledge/literature. I agree that literature on archaea in the human microbiome is limited, but some contrasts or parallels with more bacteria-focused studies could surely be made (e.g. regarding diversity, abundance, geographic patterns, virome, etc).

- The authors have made an effort to also incorporate information about abundances of archaea in the GI-tract, but the results of these additional analyses are hardly mentioned/described in the main text (tucked away in Table S15 and methods). A general overview of the distribution and abundance of archaeal lineages would be of interest to present in a figure and discussed in the main text. Which archaea are general constituents, and which more transiently associated (mentioning the potential limitations pointed out by ref 2).

- Line 371-383 and line 816-822: Some archaea (including methanogenic archaea, see here: https://environmentalmicrobiome.biomedcentral.com/articles/10.1186/s40793-016-0183-5) have been shown/are inferred to have multiple origins of replication, and are hence not suitable for analyses with tools such as iRep and GRiD to determine growth rates. The authors should acknowledge this, and tone down (or better, omit) any inferences to estimate growth rates.

Other than this I am happy to see this work published soon; it surely represents an important baseline repository for future human microbiome studies.

Reviewer #2:
Remarks to the Author:
The authors responded to all comments and mention in their rebuttal that they now also provide relative abundance estimates of domain, family and species-level taxa. However, these relative abundance data do not seem to be provided in Table S15 (see below). Furthermore, the authors refer to abundance data in the introduction "… we gain new insights into the abundance, distribution, composition and function of the human archaeome" but do not seem to include the abundances in the results section of the manuscript. Instead, they report the average breadth of coverage values, which are a good addition to the manuscript, but not a measure of relative abundance.

My further detailed comments are:

“Raw reads were obtained from studies of the human gut microbiome out of which 691 (691 of 1,167) medium- or high-quality archaeal MAGs were assembled. The rest was not made public by their original submitters (Table S1).”
Please add the number of the raw read datasets (651?) used for read mapping.

“We mapped raw reads to the 27 reference archaeal species representatives using Bowtie2-align (Langmead and Salzberg 2012) and post-processed using samtools (Li et al. 2009). The generated sorted mapping files are used to calculate the breadth of coverage (Table S15).”
The reader will appreciate more details here, e.g. how was the breadth of reference genome coverage calculated?

“Results were processed using Bracken v2.5.3 (Lu et al. 2017) and a read length of 100 and 250, which did not lead to differences, to estimate the relative abundance of domain, family and species-level taxa (Table S15).”
This sentence seems incomplete.

“Table S15. Prevalence and abundance of archaeal lineages in the human GIT. Read-based community profiler output based on UHGG catalog and the 27 archaeal species representatives of the human gut. The relative abundance of archaea was estimated at the domain, family and species-level taxa.”
Table S15 does not seem to provide relative abundances of domain, family and species-level taxa, but rather shows only the fraction of overall reads mapping to 15 species, titled “27SpeciesResp”, and a second tab lists the genome coverage percentages across all data sets.

Fig. 1
When proposing novel taxa it is important to consider the stability (e.g. bootstrap value) of the node decorated with the taxon label. The authors should provide this information in the tree, or at least state that all internal nodes decorated with new taxa have a minimum bootstrap support >90%, or similar.
Some entries in the “Genus” category are actually families, e.g. Methanomethylophilaceae - did the authors intend the show the genus Methanomethylophilus?
I am still not too excited about the way the taxa are visualised in this figure since it means going back and forth between the figure and the legend trying to decipher rather similar colours. However, if the authors opt to keep this many colours, they should at least show the taxa in the figure and the legend in the same order, e.g. start the order legend with Methanobacteriales. I also recommend checking if the colour scheme is colour-blind proof.
Furthermore, I assume the “Genome length” is the genome size which has been calculated as the average in cases where multiple genomes were recovered per species? Providing a standard deviation would be helpful. Also, the genome size of MAGs should be considered in regard to the estimated completeness.

Final Decision Letter:

Dear Christine,

I am pleased to accept your Article "A catalogue of 1,167 genomes from the human gut archaeome" for publication in Nature Microbiology. Thank you for having chosen to submit your work to us and many congratulations.

Before your manuscript is typeset, we will edit the text to ensure it is intelligible to our wide readership and conforms to house style. We look particularly carefully at the titles of all papers to

ensure that they are relatively brief and understandable.

Once your manuscript is typeset and you have completed the appropriate grant of rights, you will receive a link to your electronic proof via email with a request to make any corrections within 48 hours. If, when you receive your proof, you cannot meet this deadline, please inform us at rjsproduction@springernature.com immediately. Once your paper has been scheduled for online publication, the Nature press office will be in touch to confirm the details.

Acceptance of your manuscript is conditional on all authors' agreement with our publication policies (see https://www.nature.com/nmicrobiol/editorial-policies). In particular your manuscript must not be published elsewhere and there must be no announcement of the work to any media outlet until the publication date (the day on which it is uploaded onto our website).

Please note that <i>Nature Microbiology</i> is a Transformative Journal (TJ). Authors may publish their research with us through the traditional subscription access route or make their paper immediately open access through payment of an article-processing charge (APC). Authors will not be required to make a final decision about access to their article until it has been accepted. Find out more about Transformative Journals

<B>Authors may need to take specific actions to achieve compliance with funder and institutional open access mandates. For submissions from January 2021, if your research is supported by a funder that requires immediate open access (e.g. according to Plan S principles) then you should select the gold OA route, and we will direct you to the compliant route where possible. For authors selecting the subscription publication route our standard licensing terms will need to be accepted, including our self-archiving policies. Those standard licensing terms will supersede any other terms that the author or any third party may assert apply to any version of the manuscript.

In approximately 10 business days you will receive an email with a link to choose the appropriate publishing options for your paper and our Author Services team will be in touch regarding any additional information that may be required.

You will not receive your proofs until the publishing agreement has been received through our system.

If you have any questions about our publishing options, costs, Open Access requirements, or our legal forms, please contact ASJournals@springernature.com

An online order form for reprints of your paper is available at https://www.nature.com/reprints/author-reprints.html. All co-authors, authors' institutions and authors' funding agencies can order reprints using the form appropriate to their geographical region.

We welcome the submission of potential cover material (including a short caption of around 40 words) related to your manuscript; suggestions should be sent to Nature Microbiology as electronic files (the image should be 300 dpi at 210 x 297 mm in either TIFF or JPEG format). Please note that such pictures should be selected more for their aesthetic appeal than for their scientific content, and that

colour images work better than black and white or grayscale images. Please do not try to design a cover with the Nature Microbiology logo etc., and please do not submit composites of images related to your work. I am sure you will understand that we cannot make any promise as to whether any of your suggestions might be selected for the cover of the journal.

You can now use a single sign-on for all your accounts, view the status of all your manuscript submissions and reviews, access usage statistics for your published articles and download a record of your refereeing activity for the Nature journals.

To assist our authors in disseminating their research to the broader community, our SharedIt initiative provides you with a unique shareable link that will allow anyone (with or without a subscription) to read the published article. Recipients of the link with a subscription will also be able to download and print the PDF.

As soon as your article is published, you will receive an automated email with your shareable link.
